# Toward unsupervised outbreak detection through visual perception of new patterns

**DOI:** 10.1186/1471-2458-9-179

**Published:** 2009-06-10

**Authors:** Pierre P Lévy, Alain-Jacques Valleron

**Affiliations:** 1 Assistance Publique-Hôpitaux de Paris, Hôpital Tenon, Département de Santé Publique, F-75020, Paris, France; 2 INSERM, U707, Paris, France; 3 Université Paris 6-Pierre et Marie Curie, UMR-S 707, F-75012, Paris, France; 4 Assistance Publique -Hôpitaux de Paris, Hôpital Saint-Antoine, Unité de Santé Publique, Paris, F-75012, France

## Abstract

**Background:**

Statistical algorithms are routinely used to detect outbreaks of well-defined syndromes, such as influenza-like illness. These methods cannot be applied to the detection of emerging diseases for which no preexisting information is available.

This paper presents a method aimed at facilitating the detection of outbreaks, when there is no a priori knowledge of the clinical presentation of cases.

**Methods:**

The method uses a visual representation of the symptoms and diseases coded during a patient consultation according to the International Classification of Primary Care 2^nd ^version (ICPC-2). The surveillance data are transformed into color-coded cells, ranging from white to red, reflecting the increasing frequency of observed signs. They are placed in a graphic reference frame mimicking body anatomy. Simple visual observation of color-change patterns over time, concerning a single code or a combination of codes, enables detection in the setting of interest.

**Results:**

The method is demonstrated through retrospective analyses of two data sets: description of the patients referred to the hospital by their general practitioners (GPs) participating in the French Sentinel Network and description of patients directly consulting at a hospital emergency department (HED).

Informative image color-change alert patterns emerged in both cases: the health consequences of the August 2003 heat wave were visualized with GPs' data (but passed unnoticed with conventional surveillance systems), and the flu epidemics, which are routinely detected by standard statistical techniques, were recognized visually with HED data.

**Conclusion:**

Using human visual pattern-recognition capacities to detect the onset of unexpected health events implies a convenient image representation of epidemiological surveillance and well-trained "epidemiology watchers". Once these two conditions are met, one could imagine that the epidemiology watchers could signal epidemiological alerts, based on "image walls" presenting the local, regional and/or national surveillance patterns, with specialized field epidemiologists assigned to validate the signals detected.

## Background

Interest in syndromic surveillance was fueled in the recent years by the 9/11 attack on the US that revived fears of bioterrorism and by the threat of emerging diseases. Syndromic surveillance is defined as "an investigational approach where health department staff, assisted by automated data acquisition and generation of statistical alerts, monitor disease indicators in real-time or near real-time to detect outbreaks of disease earlier than would otherwise be possible with traditional public health methods"[1]. To achieve this goal, the places to observe and the databases to analyze must meet certain prerequisites: observations must be made where patients first seek immediate care, i.e., at general practitioners (GPs), hospital emergency departments (HED) or pharmacies. Data must be accessible in real time and collected on a routine basis. Good examples of such data are over-the-counter drug sales, visits to emergency care units, which, in most hospitals, are recorded in real time, or consultations with GPs in private practice, when a system of real-time data collection is available, as is the case for the French Sentinel Network [2,3].

Once the databases are available in appropriate settings, outbreak-analysis algorithms are needed. An outbreak is an unexpected cluster of cases of a certain category, given the past experience in the same place and under the same conditions. Thus, outbreak detection can be considered a problem of pattern recognition.

Since the advent of artificial neural network methods of pattern recognition [4], it has become classical to separate "supervised" and "unsupervised" methods. This distinction is particularly relevant in the context of outbreak detection: supervised methods are used when the pattern to recognize has been defined previously, e.g., the detection of seasonal influenza outbreaks. In this situation, data collection must rely on a well-defined set of symptoms. Then, a statistical algorithm is used to qualify when an excess of observed cases indicates an outbreak. Numerous statistical techniques belonging to this class of supervised methods are routinely used in surveillance (e.g., periodic regression models, which are now available on the Web [5]), as long as the patterns to recognize have been defined a priori. For example, the Centers for Disease Control and Prevention (CDC) defined 11 classes of syndromes associated with bioterrorism [6], and several syndromic surveillance algorithms were devised to optimally assign each new case to one of those classes [2,7-17].

While the supervised approach is straightforward, by definition, it only identifies those events that have been defined a priori. A second class of pattern-recognition techniques is the class of "unsupervised methods". In this class, the patterns to separate have not been defined previously. The challenge is to distinguish them – when they exist – from background "noise". In epidemiological terms, unsupervised approaches are suited to the detection of outbreaks of emerging diseases, for which no prior description is available, or bioterrorist attacks using unconventional biological weapons, i.e., modified biological agents causing novel unknown symptoms. Supervised methods are inapplicable in this critical role of epidemiological surveillance.

Herein, we describe an unsupervised outbreak-detection method that relies on the human visual capacity to detect new patterns [18]. This strategy is based on two components: the first is an adequate visual representation of the clinical encounters during outpatient consultations to a GP or a HED; the second is human, in that we suppose that "epidemiology watchers" could be trained to identify the novel patterns on their air-controller-like monitors, which correspond to the new epidemiological events of potential interest.

## Methods

### Outline of the method

The principle of the method relies on the translation of medical linguistic information collected during the consultation into a visual signal. To do so, the first step is to encode that information using standardized medical terminology. For this study, we chose the International Classification of Primary Care, 2^nd ^version (ICPC-2), which was specifically developed to code the clinical consultations patients in general practice. Then, these code counts, corresponding to number of consultations with those symptoms, are presented within a graphic reference frame, which contains all the codes of the terminology ordered in such a way as to mimic body anatomy. This ordering facilitates the epidemiology watcher's interpretation of the images, that we call ICPCviews.

### Coding

Each patient-practitioner encounter is described with a chain of linguistic information describing the chief purpose of the consultation. That information is then translated into ICPC-2 code [19]. It can be coded automatically [19,20] or by an expert.

### ICPC-2

In ICPC-2, the codes are ranged according to three axes: symptoms, diagnoses and processes. Within these axes, 17 social-nosological categories (social, psychiatry, neurology...) are defined. A total of 745 different codes comprise ICPC-2, compared to the 10,795 codes making up the 10^th ^International Classification of Diseases (ICD-10). In this study, we used the symptoms and diagnoses corresponding to 685 codes.

### Visualization

The aim of the method is to provide an instantaneous visualization of the whole set of codes. Herein, we applied an approach that we previously used for other medical classifications [21,22]: each ICPC-2 code is assigned to a cell located in the graphic reference frame defined below. A patient population is then represented as an image, in which each cell corresponds to one ICPC-2 code [23] and the number of affected patients is materialized by the color of this cell.

**Figure 1:**
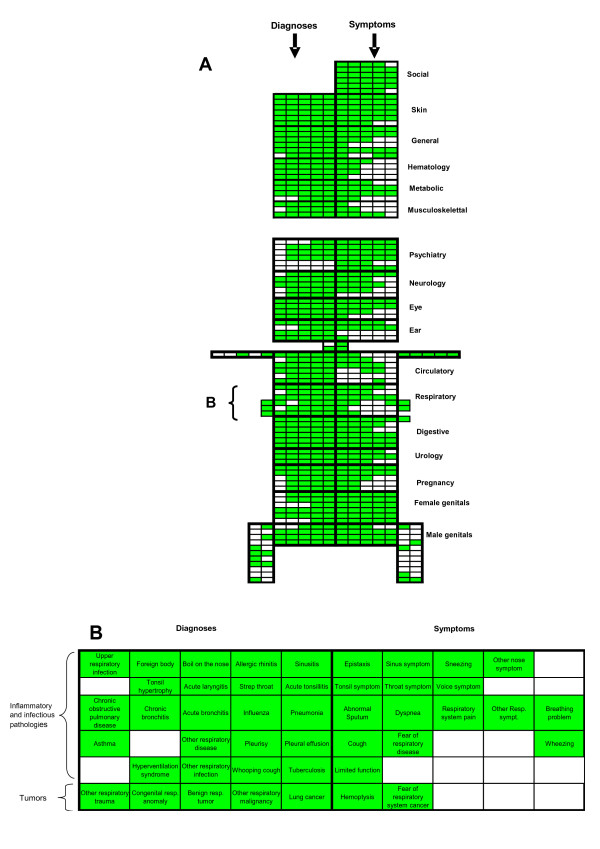
**A. Graphic reference frame of ICPCview**. Each green cell corresponds to an ICPC-2 code. The upper part corresponds to general systems (social, skin, general, hematology, metabolic, musculoskeletal). The lower part, mimicking body anatomy, corresponds to systems: the head, on the top, contains in the top-down order: psychiatry, neurology, eye pathology, ear pathology; the trunk, under the head, contains in top-down order, the rectangles corresponding to the different systems successively: cardiovascular, respiratory, digestive, urology, pregnancy, female and male genitals. The limb-like structures group the ICPC-2 codes respectively corresponding to localized pathologies of the musculoskeletal system. See Additional file 1. **B. Enlarged version of the diagnoses and symptoms of the respiratory system **Globally, there are two sets of rows: one set of four rows corresponding to "inflammatory and infectious diseases" and another set of one row, corresponding to tumors. However, some pathologies inside these sets are neither inflammatory-infectious nor tumors (for example "other respiratory trauma"). The ordinal criterion of arrangement of codes may be fuzzy: for example asthma, which is far from the medial line, has various manifestations, some severe or others not. It was placed here to be close to "Chronic obstructive pulmonary disease", which is above it. The pathologies outside the rectangle, which belong to the musculoskeletal system, have been removed.

The graphic reference frame (Figure 1A and Additional file 1) was built using three criteria. First, a binary criterion splits the reference frame into two symmetrical parts separated by a medial dividing line: the diagnoses are placed on the left and the symptoms on the right. Second, the different ICPC-2 headings are arranged vertically in successive rectangles representing a particular system, in which the codes are organized in rows corresponding to tumor pathologies, degenerative pathologies, traumas, inflammatory and infectious pathologies, pathologies specific to the system. Third, individual ICPC-2 codes are placed horizontally. The codes the easiest to recognize, because they are the most serious or because they correspond to well-defined nosological entities, are placed closest to the medial vertical line. For example, the code R06, epistaxis, is adjacent to the medial line while the code R09, sinus symptom, has a more distant position. An enlargement of the respiratory system codes is shown in Figure 1B.

Each color is coded by a number reflecting the frequency of the diagnosis/symptom, from white (absent) to bright red (= 255). Let's assume that, in a given cell, a frequency x was observed. The color code N(x) assigned to x is defined by N(x) = integer part of [(x/(Max - Min)) × 255], where Max and Min are the respective maximum and minimum numbers of medical consultations associated with the symptom codes corresponding to the image cells for this population of patients.

### Settings

We used two databases to illustrate our method: one from GPs in private practice and the other from an HED.

Data from GPs were obtained through the French Sentinel Network which has monitored online a series of common communicable diseases since 1984 [2] and, all patient referrals to hospitals by Sentinel GPs since August 1997. A program then converts, when possible, these referrals, which are expressed in free text, into ICPC-2 codes [20]. A total of 17,896 consultations were notified between 1997 and 2004: half were automatically coded by the software, the other half were coded by a medical resident.

Data on outpatients consulting at an HED were obtained during 2006 (n = 45,055) in a major university hospital in Paris. The chief complaints of every consulting patient during 2006 were recorded in free text by the triage nurse. To determine whether characteristic patterns of influenza were visible before the 2006 outbreak, we selected 4 random subsets of 200 consultations each, corresponding respectively to: the week just before the flu period (week 3/2006), the week of the flu outbreak peak (week 6/2006), the week after the flu period (week 14/2006) and the rest of the year. The time of the epidemic peak and its duration were provided by the routine periodic regression software used for real-time data from the Sentinel Network [24]. The chief complaints of these 800 patients were coded by an experienced ICPC-2-coding medical secretary.

## Results

**Figure 2:**
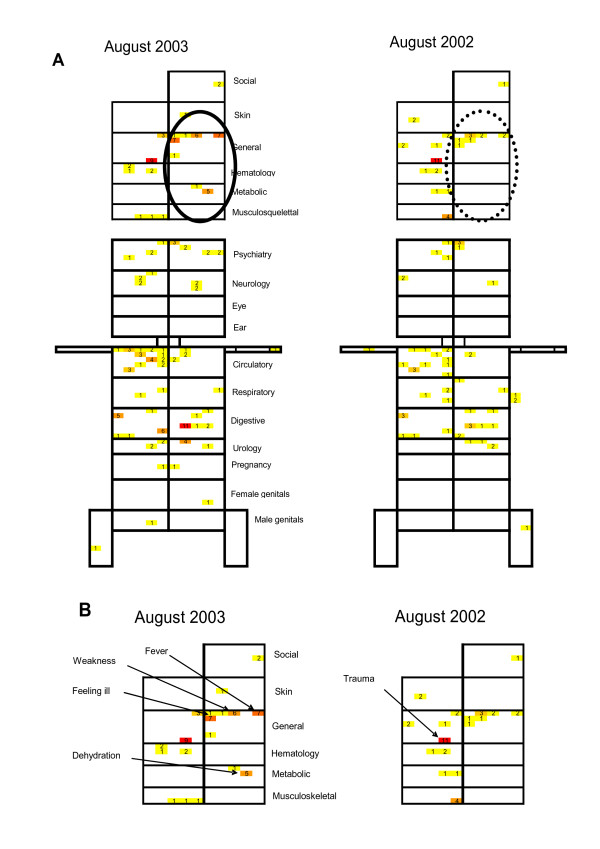
**A. ICPCview of the Sentinel Network referral data: comparing the deadly August 2003 heat wave to August 2002**. Each cell contains the number of patients seen by the GP members of the French Sentinel Network for the specified ICPC-2 code and referred for hospitalization. Note that color range from white (no hospital referrals) to red (highest number of referrals) facilitates visual appreciation of the intensity of diagnoses and symptoms. **B. Enlarged views of patterns generated in August 2003 (heat wave) and August 2002**.

The first example of epidemiological detection of events of interest obtained with the GPs' data, shown in Figure 2A, focused on the summer of 2003, when France was hit by a heat wave that killed more than 15,000 persons [25]. A specific pattern (Figures 2A and 2B) corresponding to general and metabolic symptoms (fever, weakness, impaired general condition and dehydration) can be easily recognized.

We also analyzed the GPs' data concerning influenza-like surveillance over a 7-year period (see Additional file 2, which contains a slide show composed of 82 successive images). The visual changes of disease-associated color patterns generated by the successively entered codes that paralleled flu epidemics are apparent [26]. Note that this example is not provided to proclaim that the visual method should replace in this instance the classical supervised statistical outbreak-detection methods, which are used routinely. It is given as proof-of-concept of the proposed unsupervised method, as it enables the recognition of the influenza outbreaks that are objectively defined with the supervised techniques.

**Figure 3:**
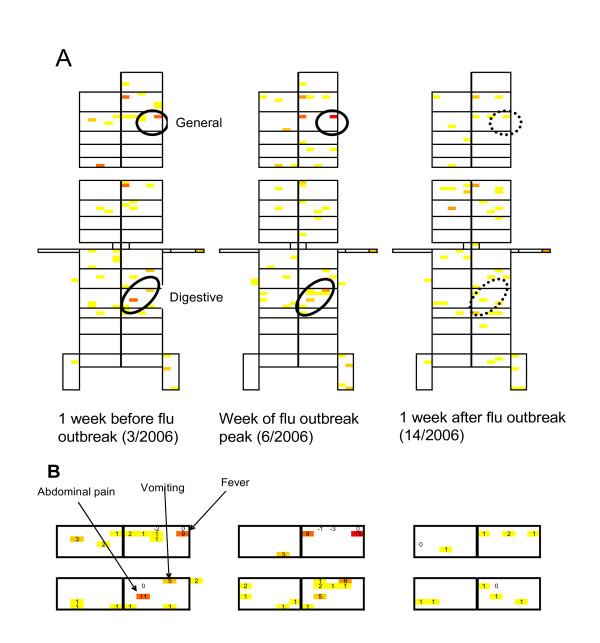
**A. These ICPCviews correspond to 3 specific weeks surrounding the 2006 flu outbreak**. The patient numbers were subtracted from the control ICPCview of 200 codes randomly chosen from the rest of the year; only the positive differences have been retained. Color coding is as described in the legend to Figure 2. The outbreak period was defined using the Serfling method [27]. **B. Enlarged views of the General and Digestive patterns generated during the 3 weeks of interest**.

In the second example, we used data from an HED to evaluate whether the technique was able to detect an outbreak of flu-like disease in a timely manner in another setting, with different patients, coded differently. This example also shows that standard methods of image analysis (such as subtraction of images, smoothing, etc) can be used in the present application. We examined the ICPCviews corresponding, as above, to: the week just before the outbreak (week 3/2006), the week at the outbreak peak (week 6/2006), the week after the outbreak (week 14/2006), and the rest of the year taken as a control period. We then subtracted the control ICPCview from the three ICPCviews being considered. The pattern for the week preceding the outbreak ICPCview clearly foresees that seen during the week of the outbreak peak (Figures 3A and 3B) [27]. This pattern combines general symptoms (fever) and digestive symptoms (abdominal pain and vomiting), which are what is to be expected during an influenza-like or gastroenteritis outbreak. At this point, field epidemiologists and reference laboratories are needed to finalize the characterization of the epidemic detected.

## Discussion

The usual syndromic surveillance methods are supervised and based on statistical tools. Herein, we described a novel method that could be used when the supervised approach is not applicable. That situation occurs when we are faced with the detection of "unexpected" events, which, by definition, are of major interest for epidemiological alert. Indeed, our primary goal is to help recognize, as early as possible, totally unexpected epidemiological patterns. The detection-triggering signal can be the mere increase of an isolated diagnosis code. Pertinently, in this case, a regression method with a threshold would have performed better only under the very restrictive condition that this code would have been identified in advance. However, the signal can also be an unusual association of different color patches on the monitor, which appear to be novel to the observer, and trigger an in depth epidemiological investigation.

Our proposed model is similar to what already happens in an air-traffic-control room: most of the routine tasks are now automated and the attention of the human observers is now focused on "unexpected" events. Likewise, we propose relying on the classical supervised methods for the usual situations that happen regularly (e.g., seasonal flu epidemics), and we seek to improve our detection of the unexpected epidemiological events that are extremely critical from a public health perspective, precisely because they are unexpected.

An important technical problem is the choice of the time resolution of the display on the monitor. For the French Sentinel Network, resolution time was the month: that timeframe is clearly irrelevant for prospective surveillance and was only used to show the potential of our method to recognize a very special event (i.e., the health impact of the 2003 heat wave). Similarly, for the HED, the choice of weekly resolution was only illustrative and was imposed by the numbers of data available per day, keeping in mind that several hundred cases are needed to create an informative image. In a real-world application, the choice of the temporal resolution would depend on the nature of the class of events to be identified: hourly resolution or, at worst, daily resolution would be desirable to recognize a terrorist attack-associated disease. The temporal resolution chosen also reflects the spatial resolution, with the number of cases observed indeed being a decreasing function of both spatial and temporal resolutions.

For example, the HED data we used in our example was collected in real time. The hospital that provided those data has ~150 consultations per day. Using a surveillance system based on the network of all Paris region public hospitals (Assistance Publique-Hôpitaux de Paris), which collects real-time data on 4000 patients per day (i.e., ~150/patients/hour) would, in contrast, empower a much shorter timeframe, of the order of a few hours.

Furthermore, for the method we propose, we chose to code diagnoses and symptoms with the ICPC-2 system, because it was developed precisely for primary care patients, who are the best target for surveillance of emerging diseases or bioterrorist attacks. However, the same paradigm developed herein could be used with other classification methods.

In a first test example, we showed that visual inspection of the ICPCviews obtained based on Sentinel Network GPs' transmissions during the 2003 heat wave in France would have likely raised suspicion that something unusual was occurring at that time. Indeed, in light of the public health and political scandal that ensued, it is highly rewarding that the images generated with our model heralded the high morbidity and mortality (later documented) that passed unnoticed. At the time of the event, the only public health warnings came from newspapers and funeral parlors, not from the health information systems, which were therefore far from the ideal real-time systems we described above. Imagine that the wall of monitors would have generated patterns similar to those seen in Figure 2, derived from data collected throughout the country. We are convinced that the trained "epidemiology watchers" would have detected the unexpected patterns and would have triggered the investigations that were so sorely lacking.

The second example we used was the detection of a flu-like outbreak. Flu-like symptoms are observed at the onset of many diseases, during bioterrorist attacks (e.g., smallpox, plague, anthrax), and for emerging diseases (e.g., severe acute respiratory syndrome, Chikungunya, flu pandemic,...). Numerous supervised techniques proved successful at recognizing seasonal influenza outbreaks, and the goal of our technique is not to compete with those methods in this situation.

Now, imagine an outbreak of influenza-like syndromes occurring in August, or the onset of a new disease heralded by symptoms, like epistaxis or purpura; the supervised methods would be, by definition, unsuited to detect them, while an unsupervised technique, like the one we proposed here, could work. Finally, the method is designed to have the highest sensitivity possible, in order to detect rare, unusual and unexpected signals. To achieve good positive-predictive value would require, in addition, a "back room", where human experts would validate the signals based on appropriate field epidemiological investigations.

One caveat of the method is that it relies, by definition, on human observers. Hence, its effectiveness will depend upon the quality of these observers and their training. The system's quality and that of the epidemiology watcher could be measured with a research protocol based on simulated datasets. This approach has been successfully used in epidemiological surveillance to test new algorithms [28]. Simulated data sets could be generated by adding a given number of codes of interest (e.g., those compatible with an anthrax attack) to an existing database (e.g., the present HED database). Epidemiology watchers would then be shown the successive monitors displaying the evolution of the images within the graphic reference frame, and asked to indicate whether and when they could identify an outbreak. Such a design would allow easy computation the sensitivity and specificity of the system (as a function of the number of simulated codes added to the database). Standard statistical techniques would also allow assessment of intra- and interobserver variabilities.

## Conclusion

If one accepts that an epidemiological alert system must be able to detect unexpected events, then huge efforts must be made to develop unsupervised methods precisely designed with this effect in mind. Herein, we described an attempt in this direction. The use of visual perception that we advocate here is not the only possible solution. Unsupervised pattern recognition is a prolific field of research that takes advantage of the ever-increasing power of computers and the new methods of machine learning. Those will be new avenues for epidemiological research into efficient warning systems.

## Competing interests

The authors declare that they have no competing interests.

## Authors' contributions

A-JV proposed using the present visual method for outbreak detection. PL defined the visual graphic reference frame and selected the databases used to exemplify the method. A-JV and PL wrote the paper jointly.

## Pre-publication history

The pre-publication history for this paper can be accessed here:



## Supplementary Material

Additional file 1 **ICPC-body**. ICPC classification with our visual reference frame Click here for file

Additional file 2 **Movie of flu epidemics**. This slide show is composed of 82 successive images of the graphic reference frame corresponding to the ~7-year period and illustrates the visual changes of the color patterns of the disease-associated ICPC-2 codes generated by the successively entered codes that paralleled flu epidemics. Click here for file
