# Supplementary material for: Toward unsupervised outbreak detection through visual perception of new patterns
Source: BMC Public Health. 2009 Jun 10;9:179. doi: 10.1186/1471-2458-9-179 (PMC2706246; doi:10.1186/1471-2458-9-179)
Supplement: Additional file 2 — Movie of flu epidemics. This slide show is composed of 82 successive images of the graphic reference frame corresponding to the ~7-year period and illustrates the visual changes of the color patterns of the disease-associated ICPC-2 codes generated by the successively entered codes that paralleled flu epidemics. [file 1471-2458-9-179-S2.ppt]

## Slide 1
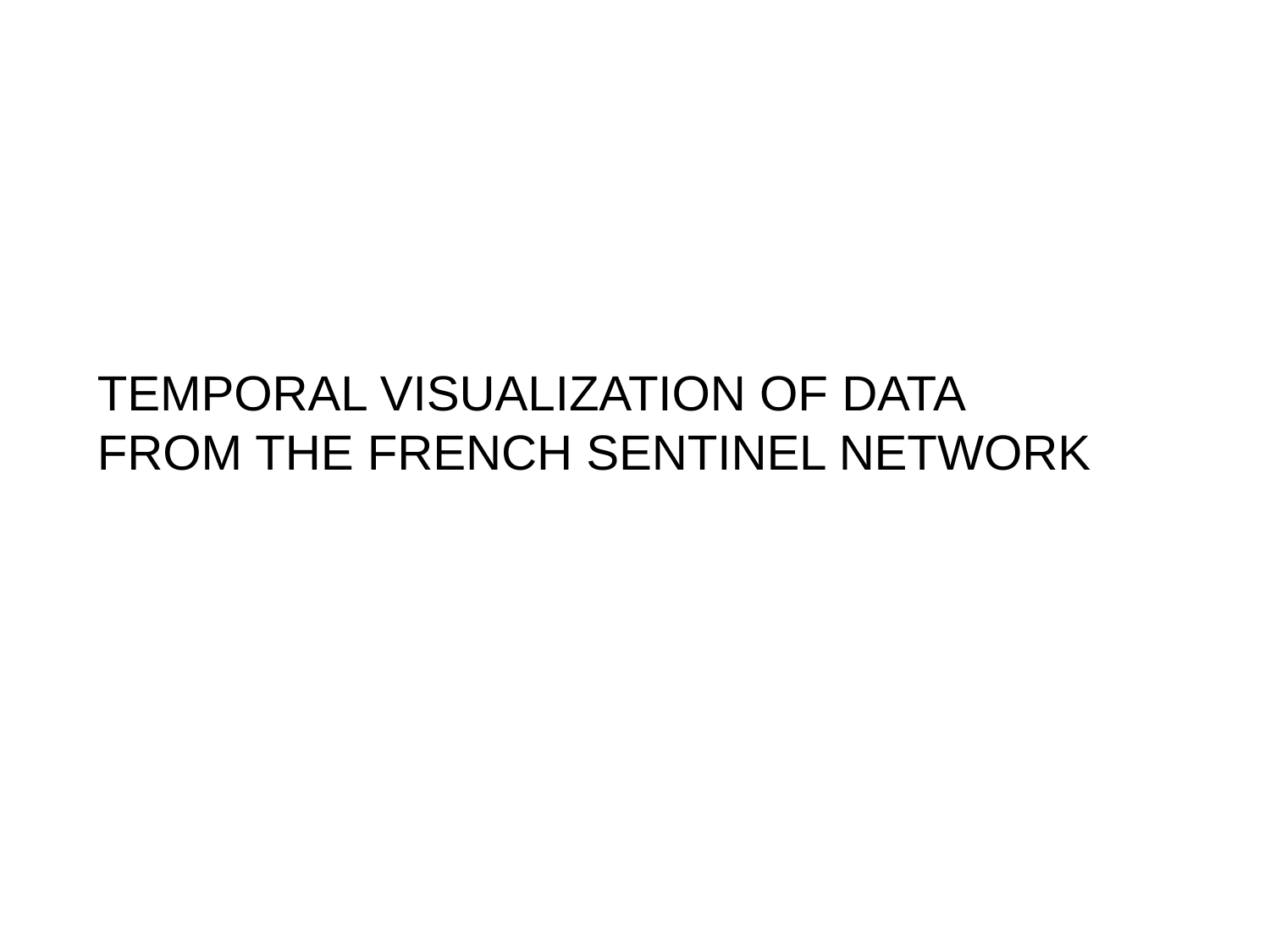

# TEMPORAL VISUALIZATION OF DATA FROM THE FRENCH SENTINEL NETWORK

## Slide 2
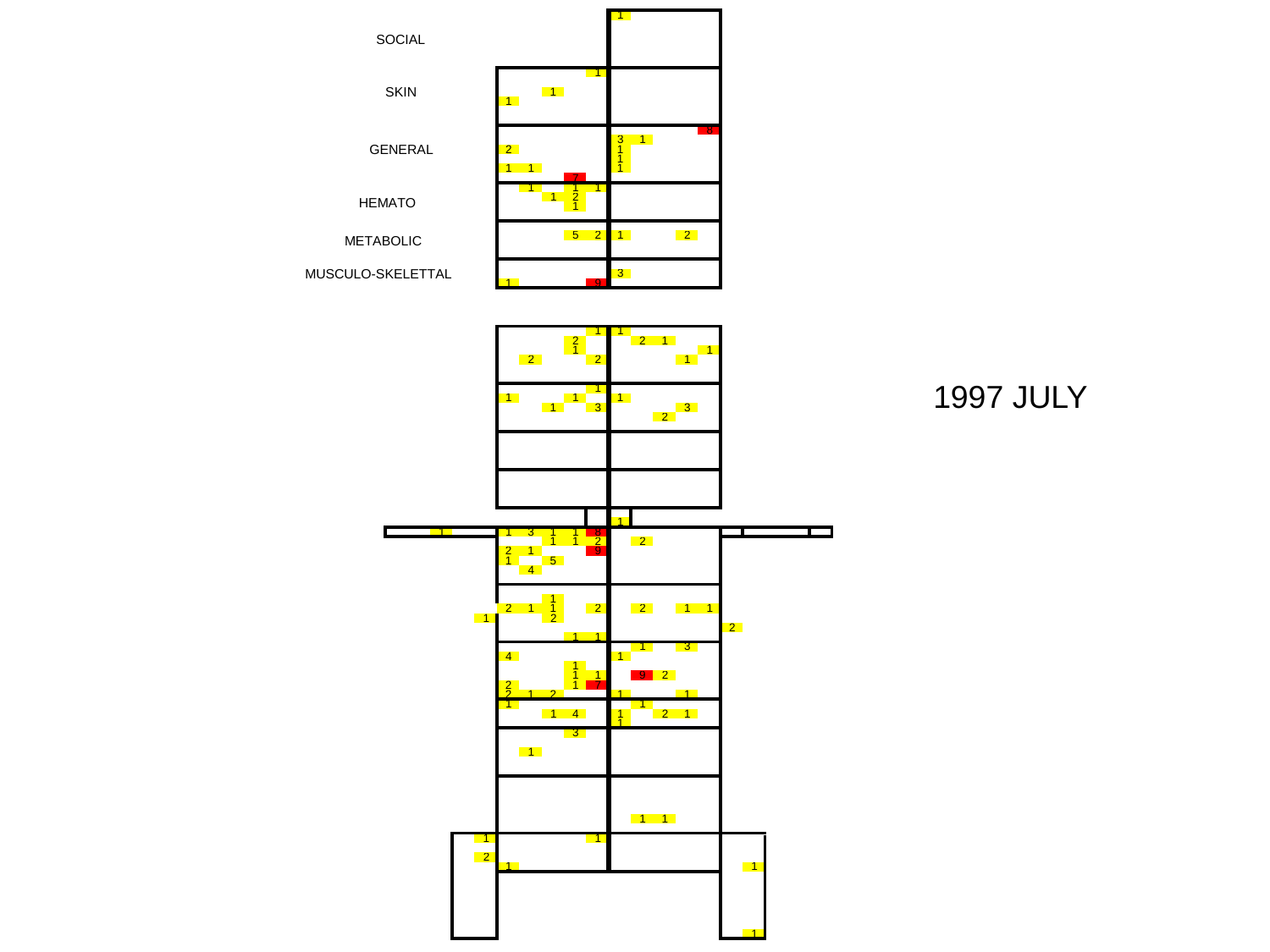

1997 JULY

## Slide 3
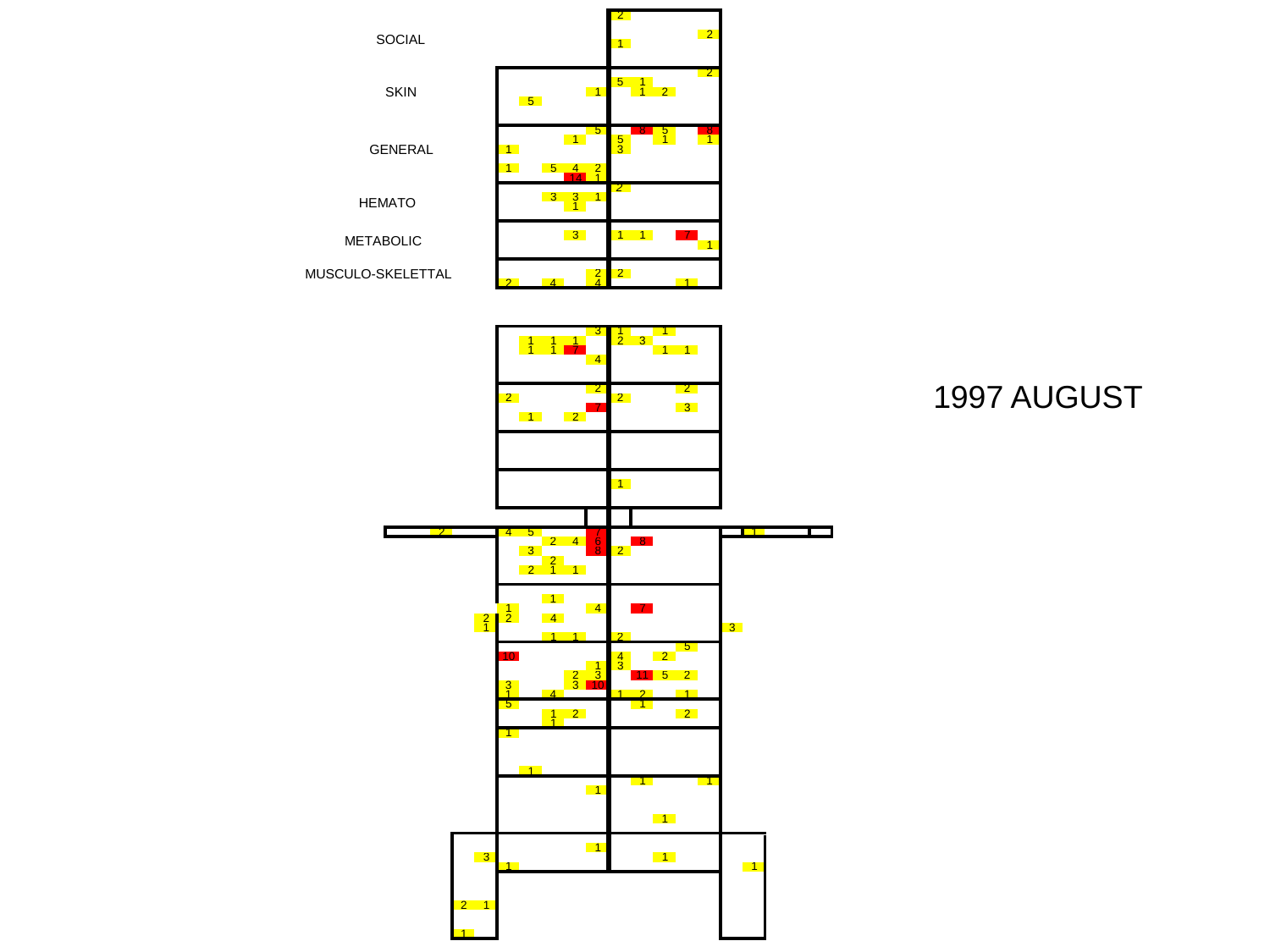

1997 AUGUST

## Slide 4
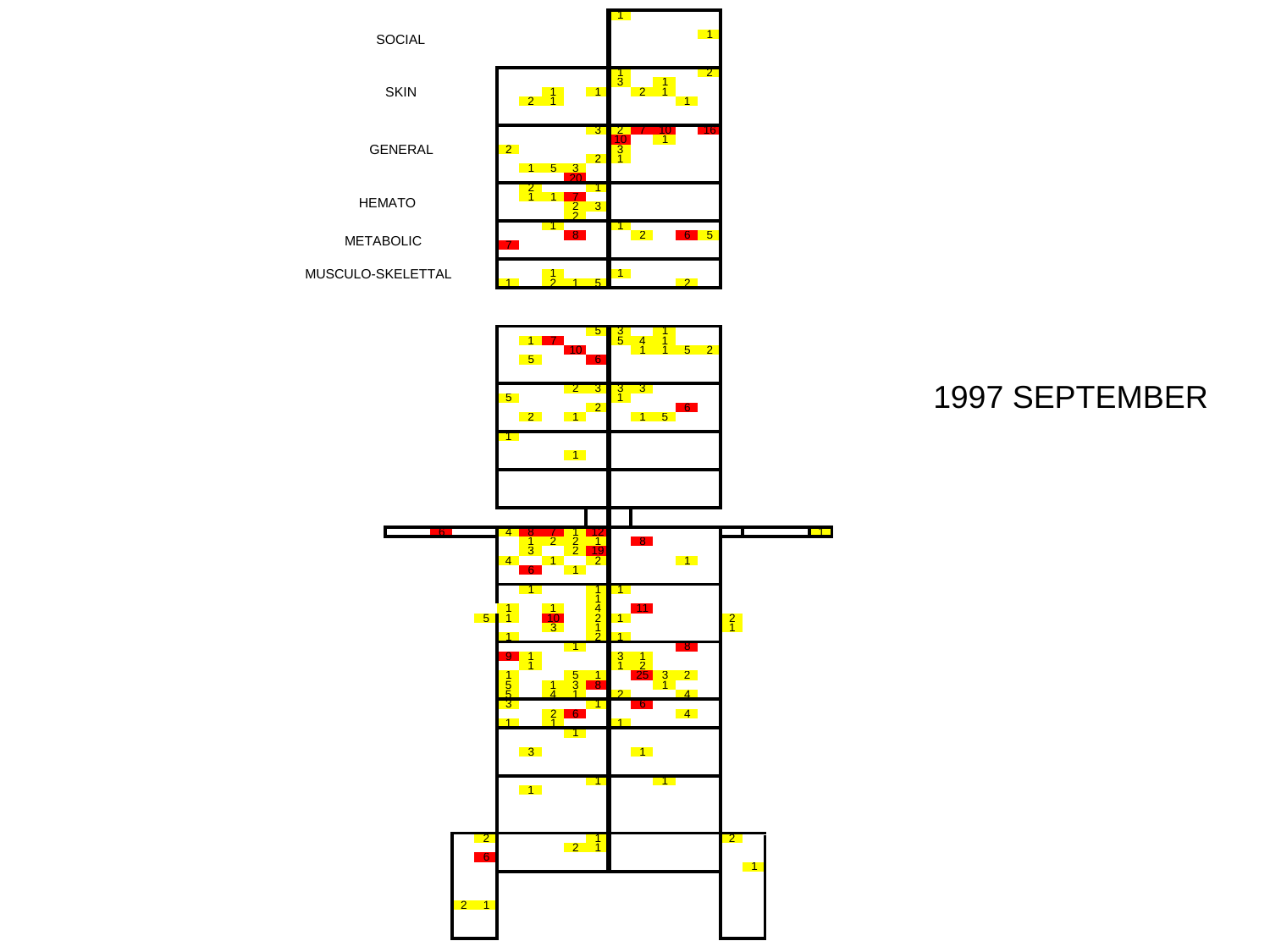

1997 SEPTEMBER

## Slide 5
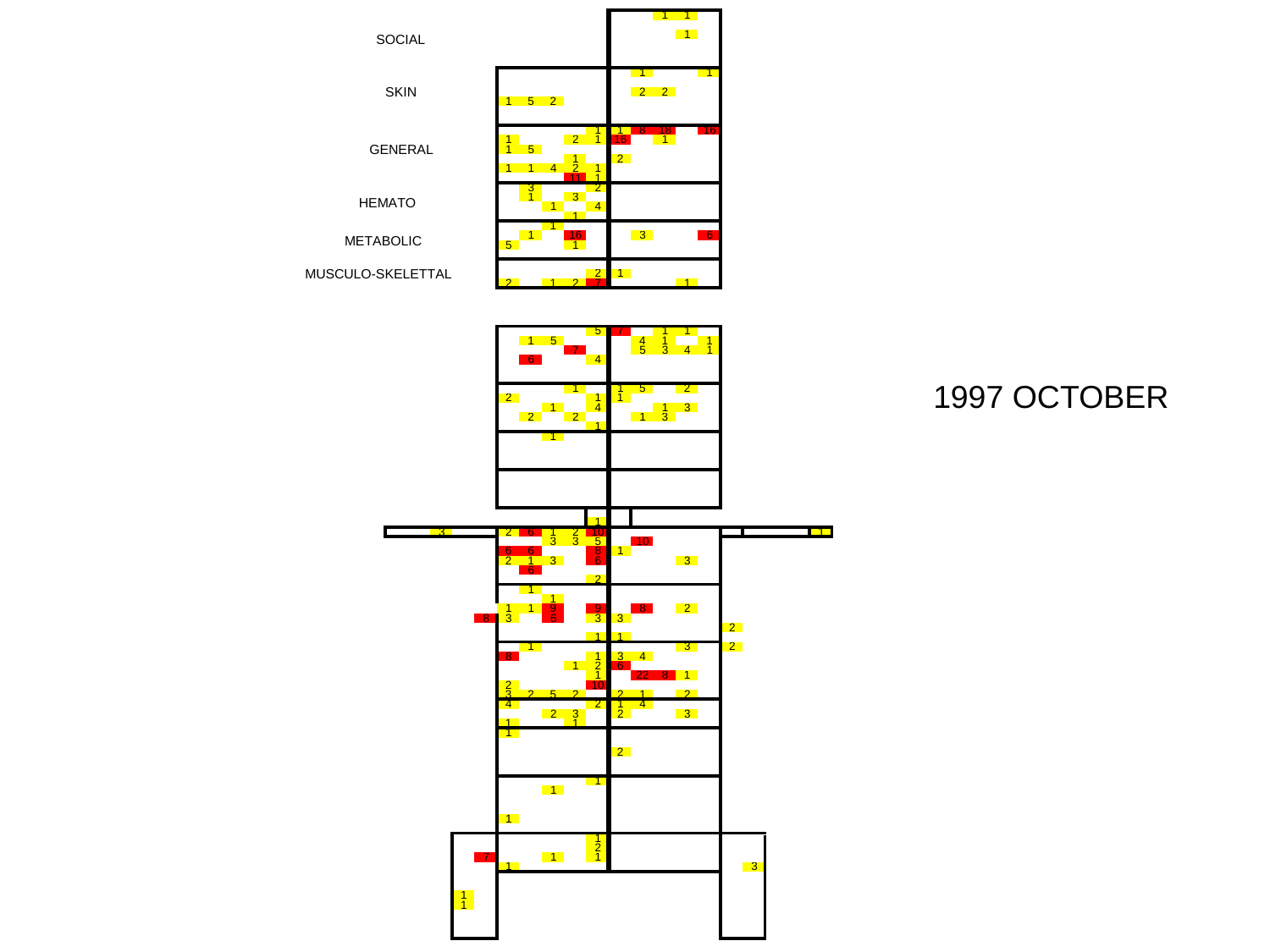

1997 OCTOBER

## Slide 6
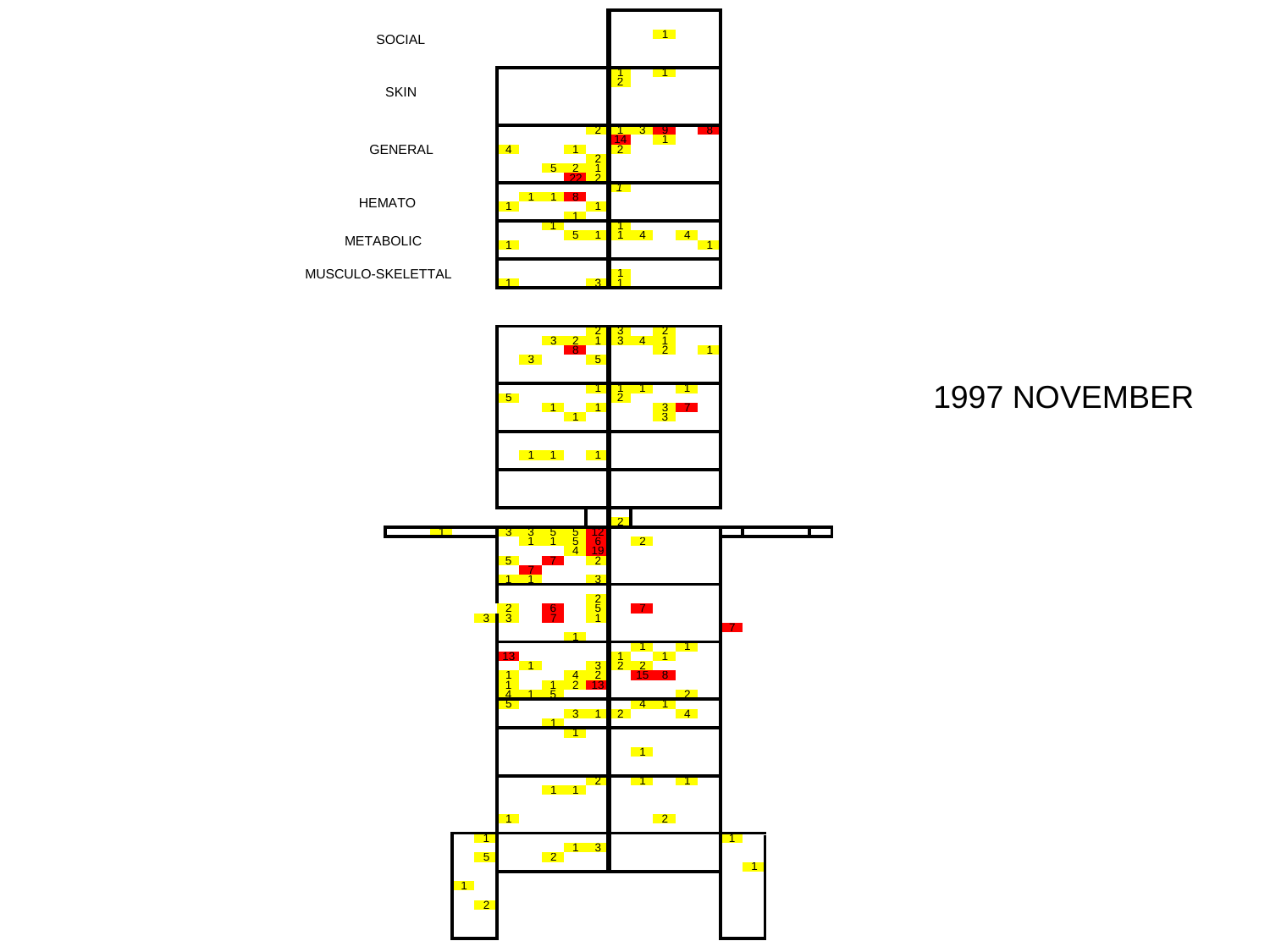

1997 NOVEMBER

## Slide 7
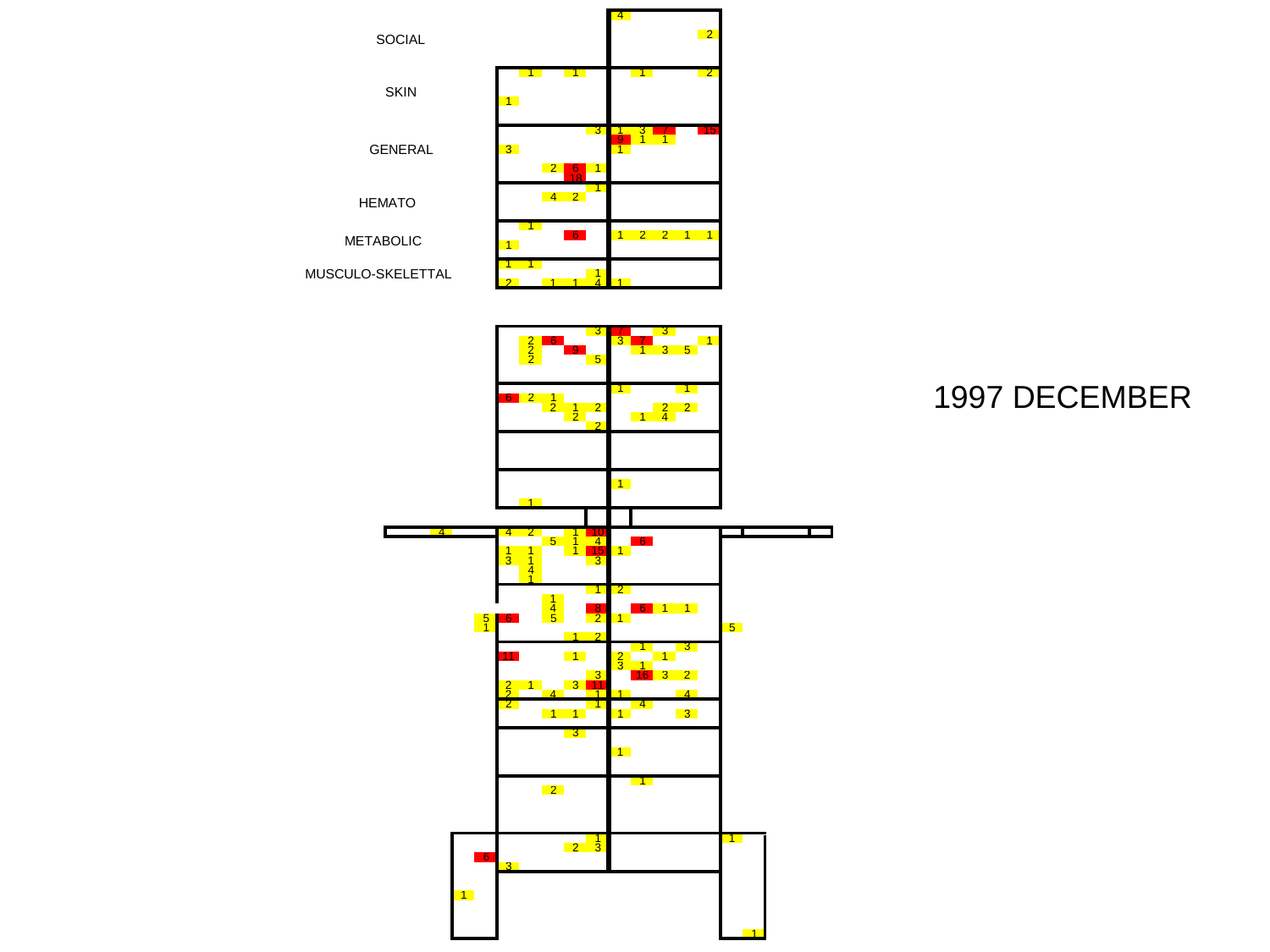

1997 DECEMBER

## Slide 8
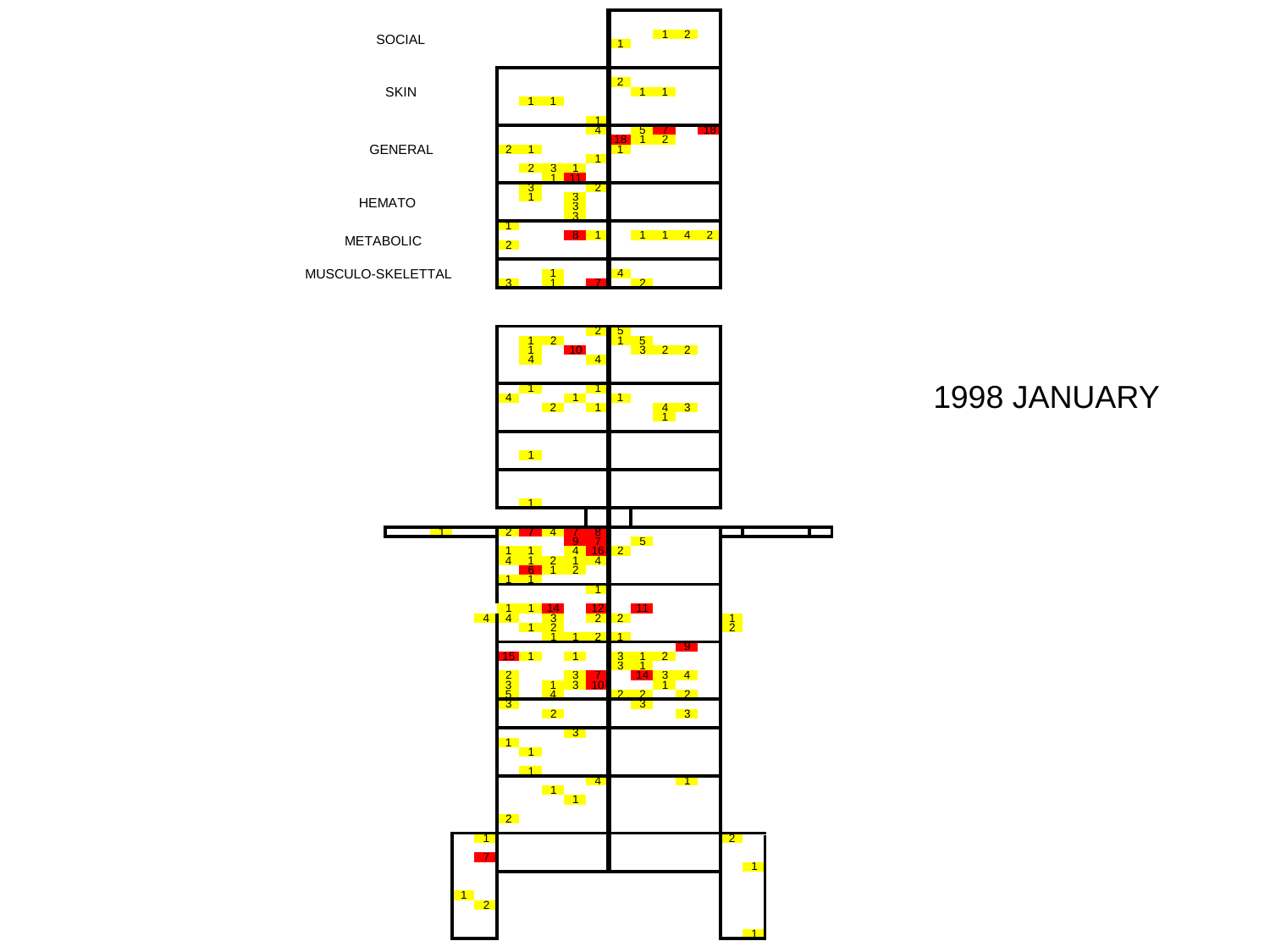

1998 JANUARY

## Slide 9
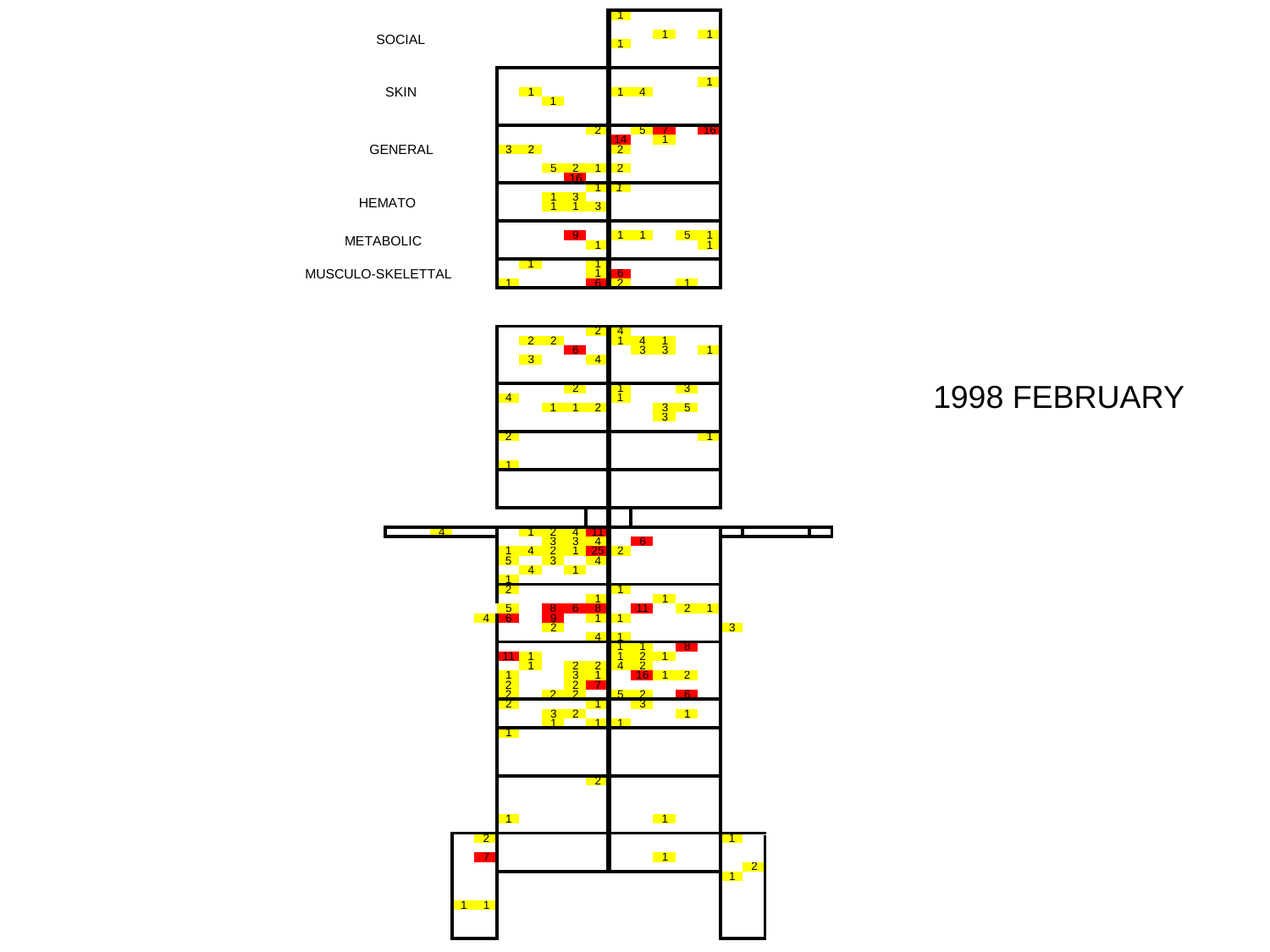

1998 FEBRUARY

## Slide 10
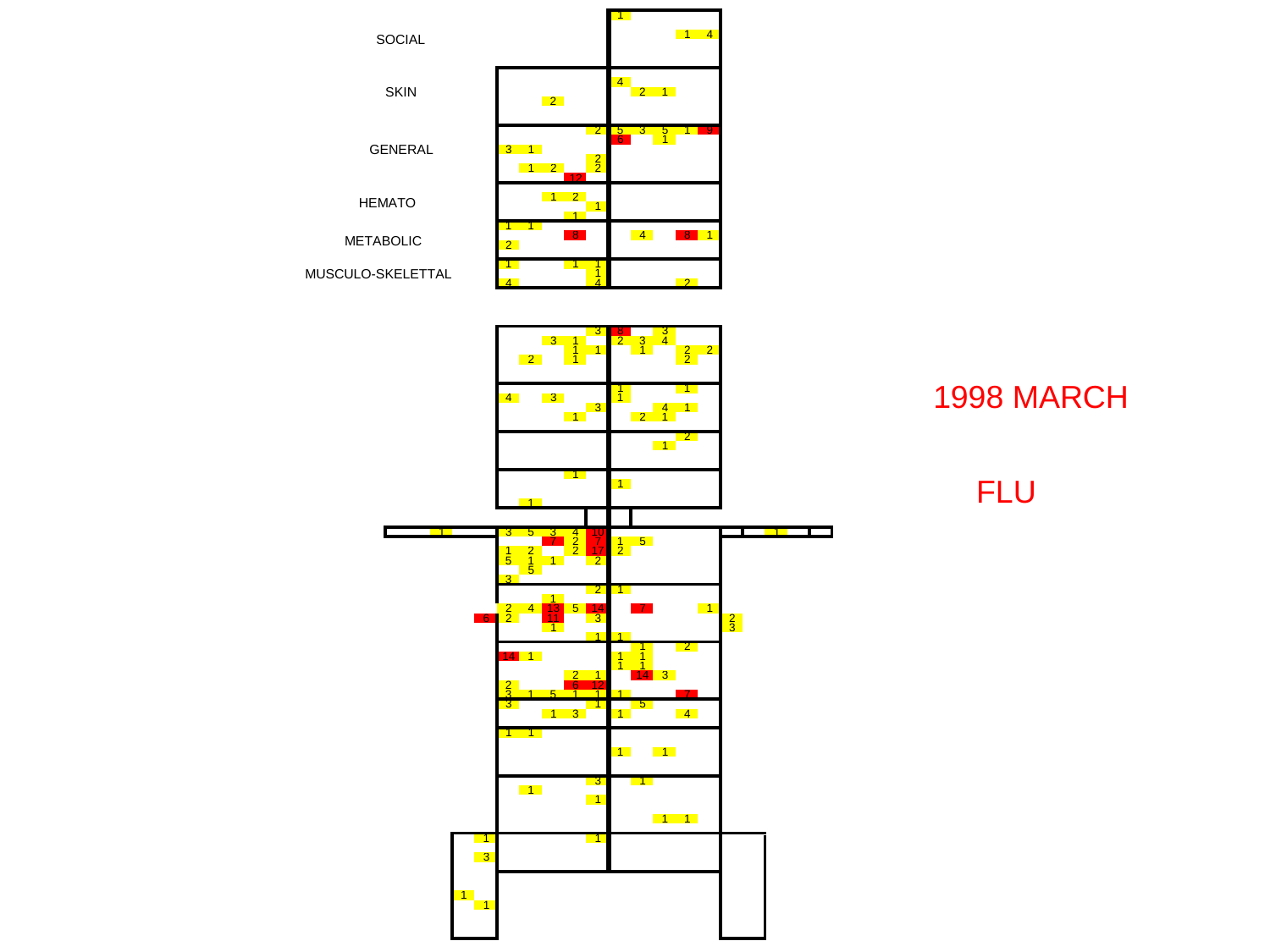

1998 MARCH
FLU

## Slide 11
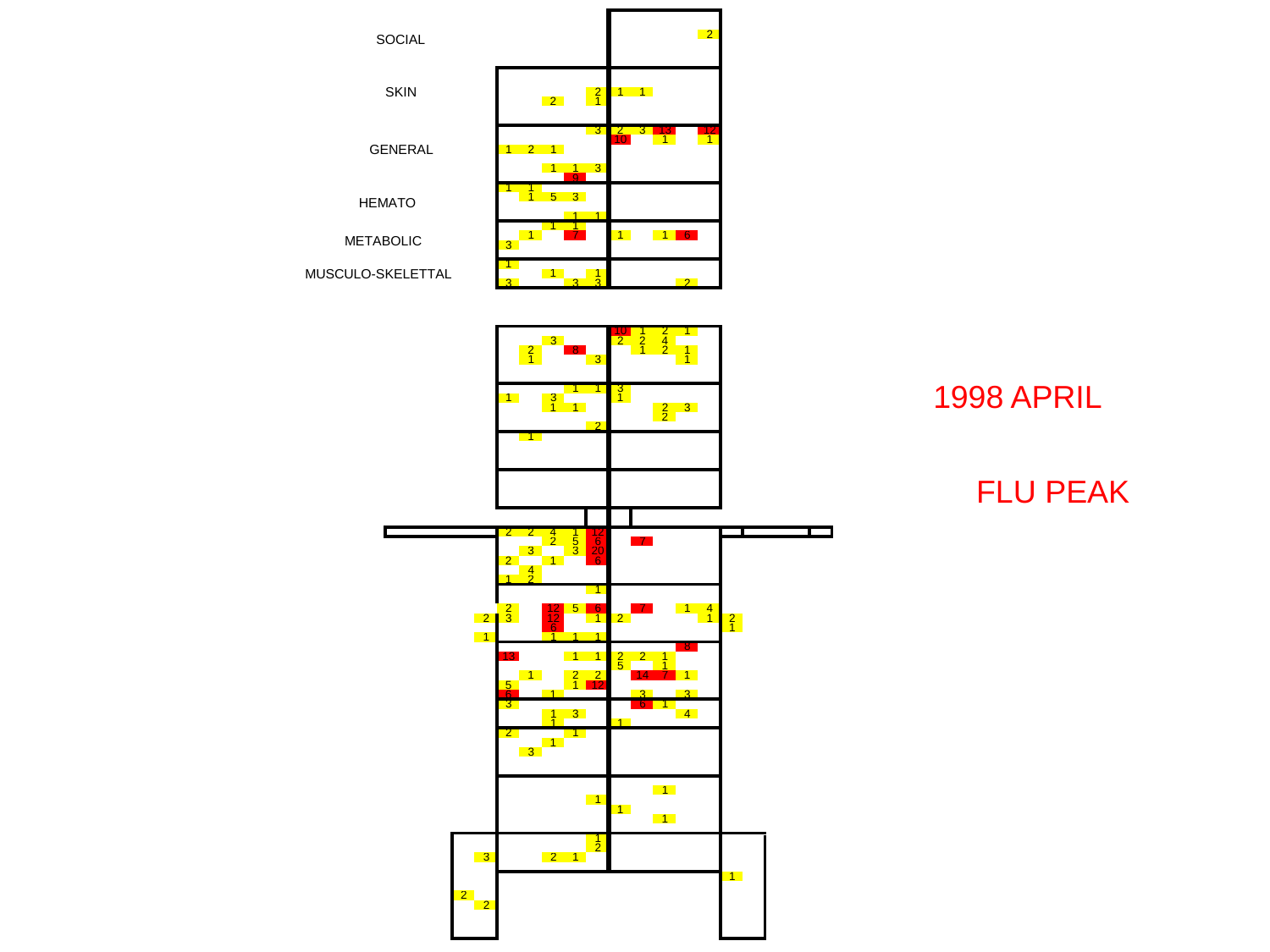

1998 APRIL
FLU PEAK

## Slide 12
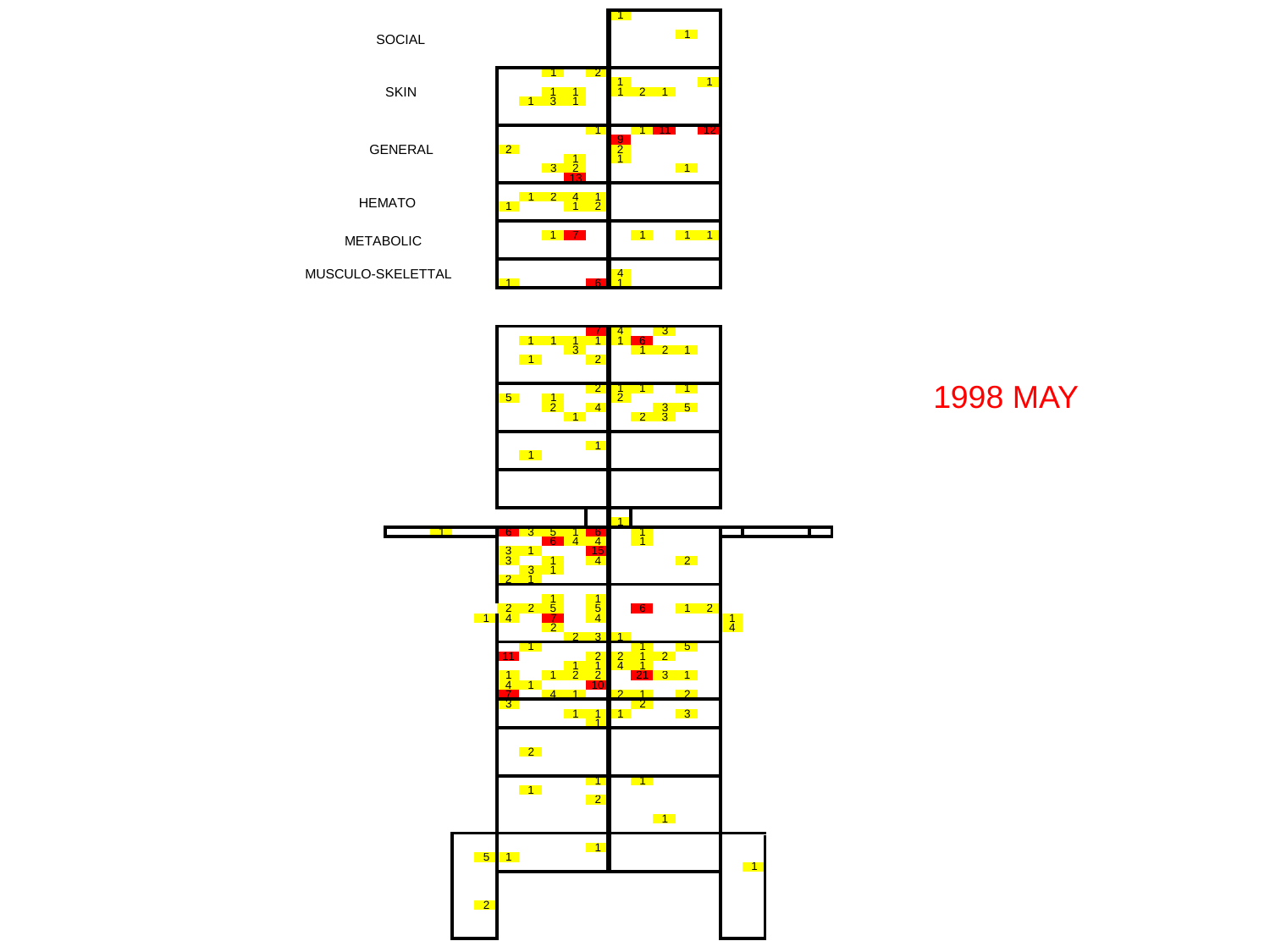

1998 MAY

## Slide 13
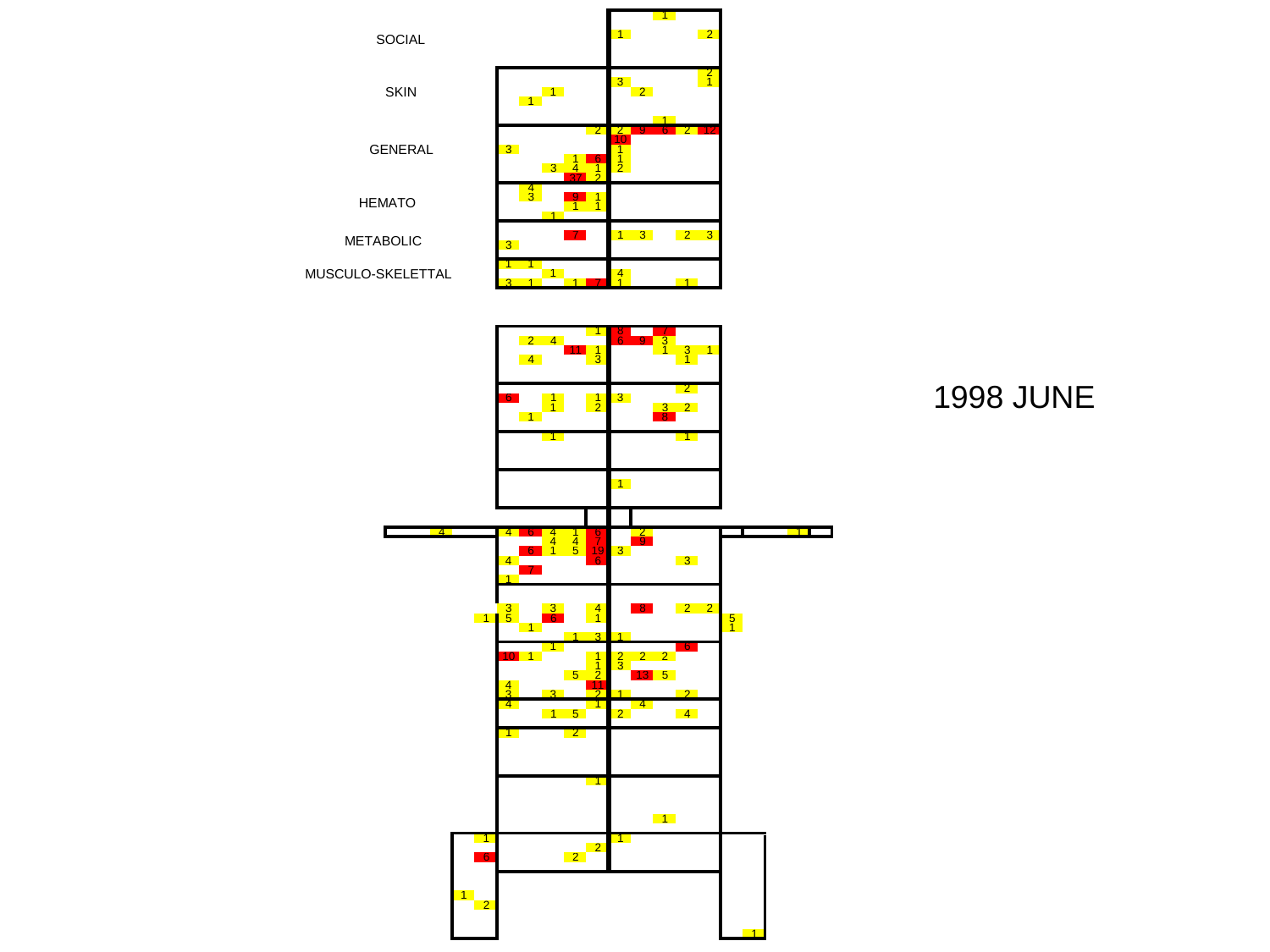

1998 JUNE

## Slide 14
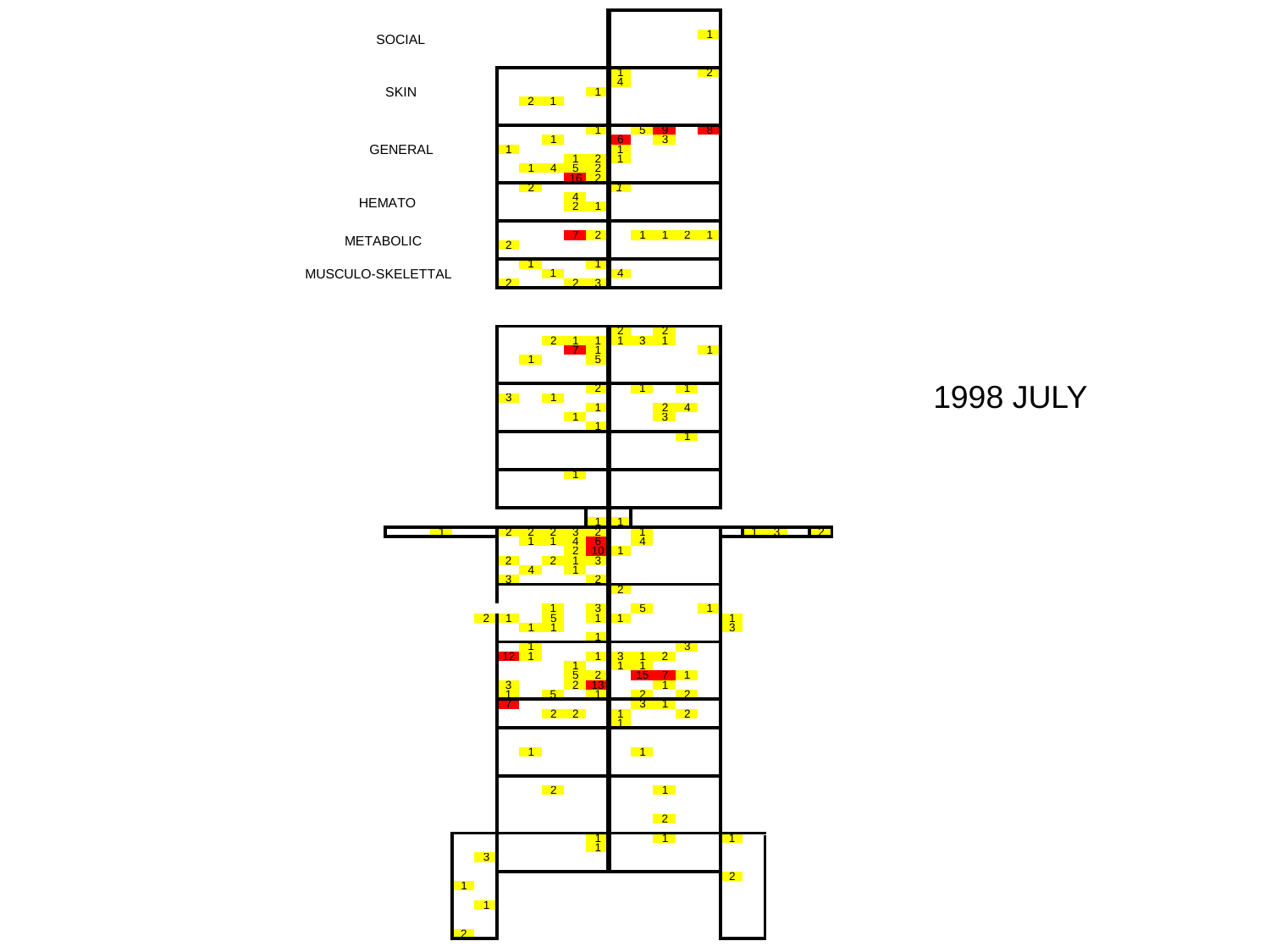

1998 JULY

## Slide 15
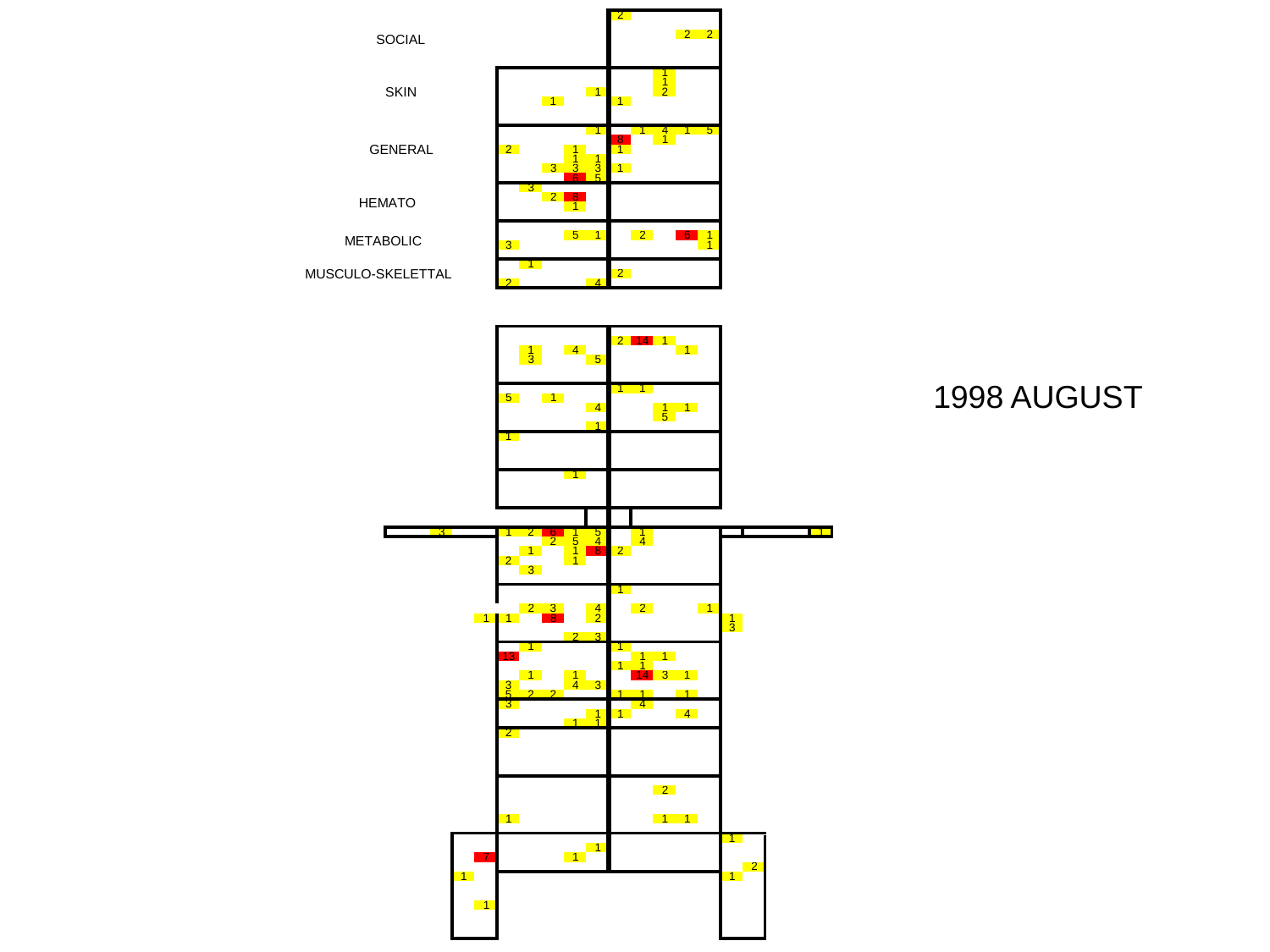

1998 AUGUST

## Slide 16
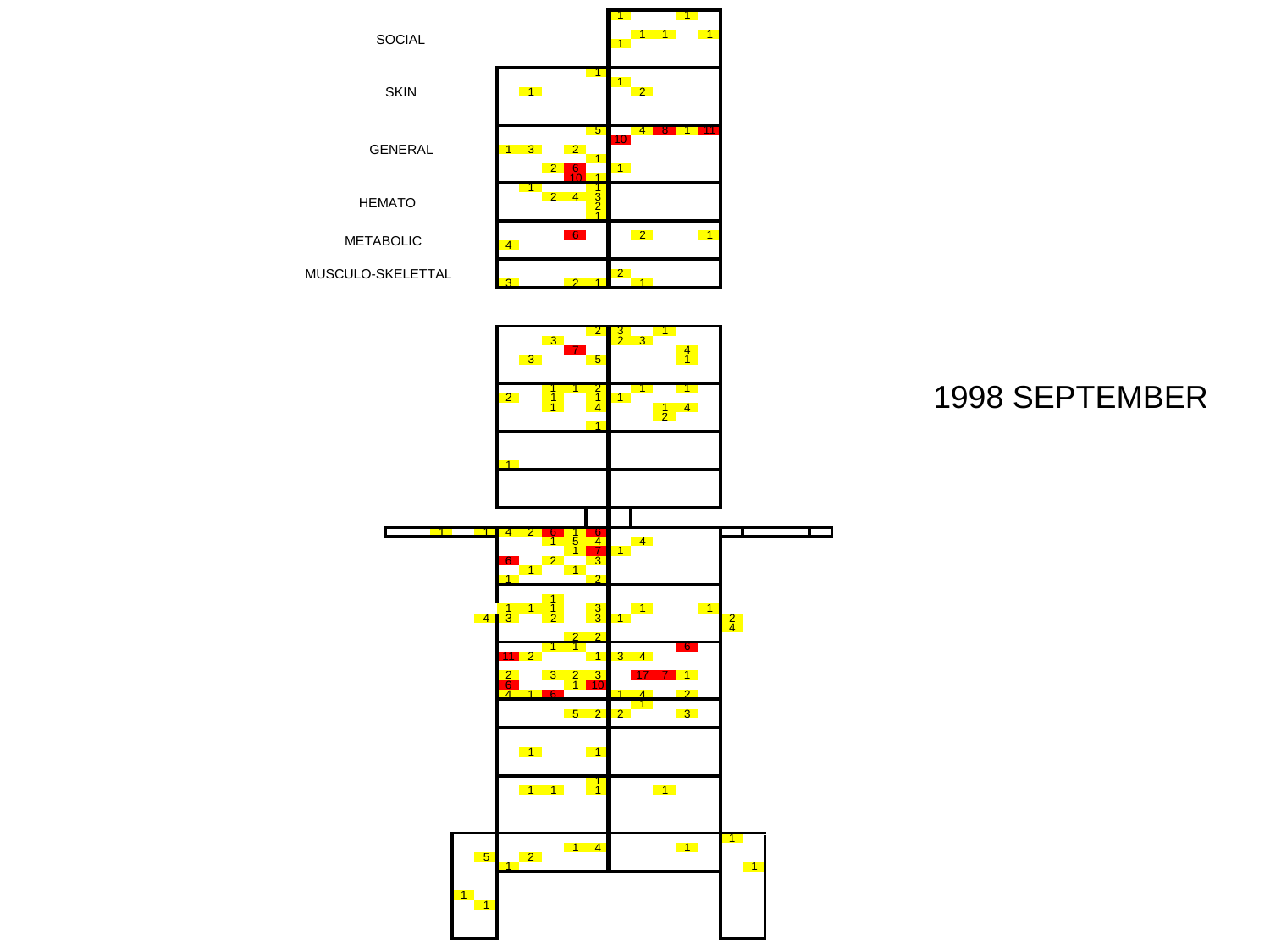

1998 SEPTEMBER

## Slide 17
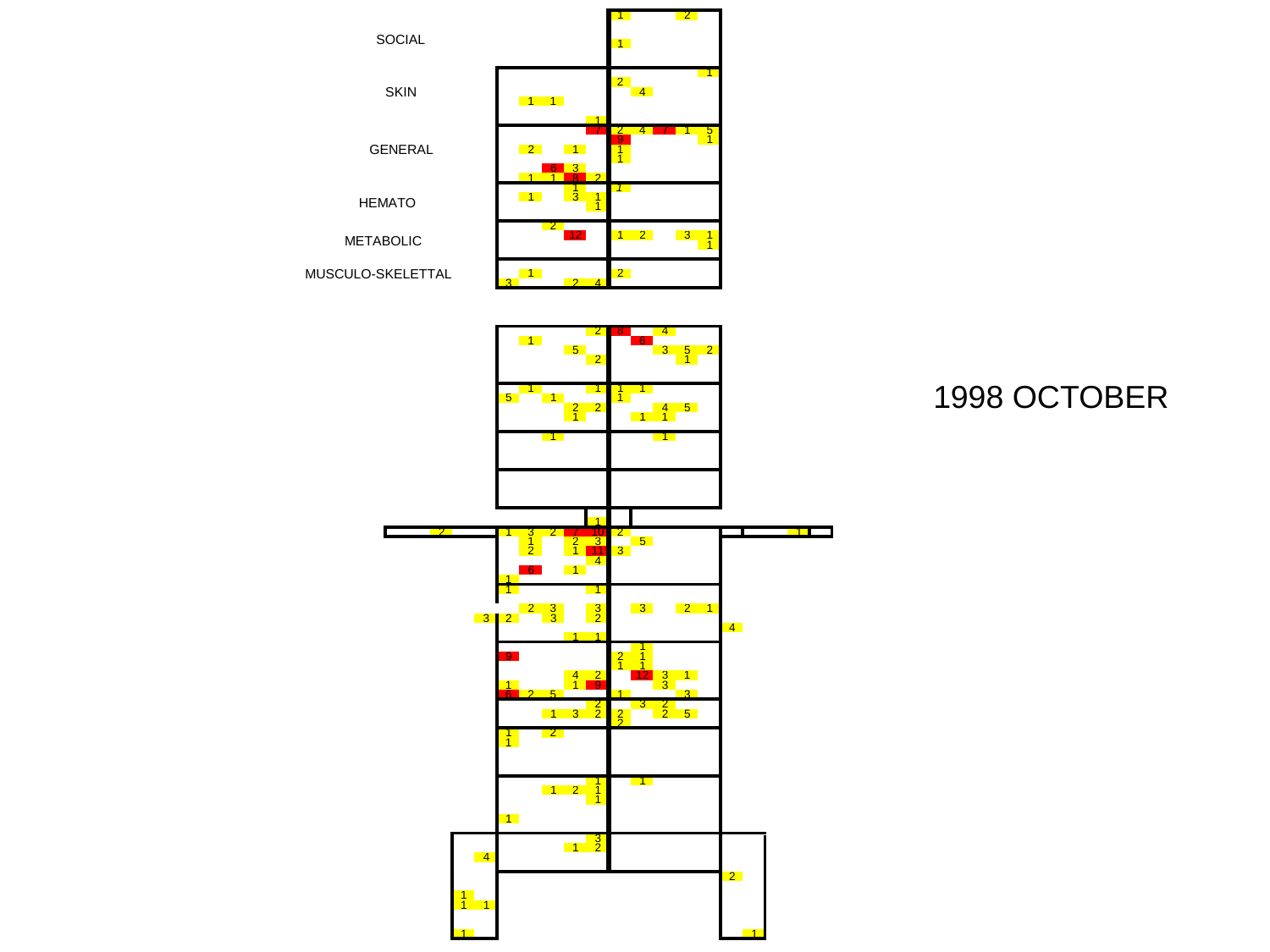

1998 OCTOBER
1998 NOVEMBER

## Slide 18
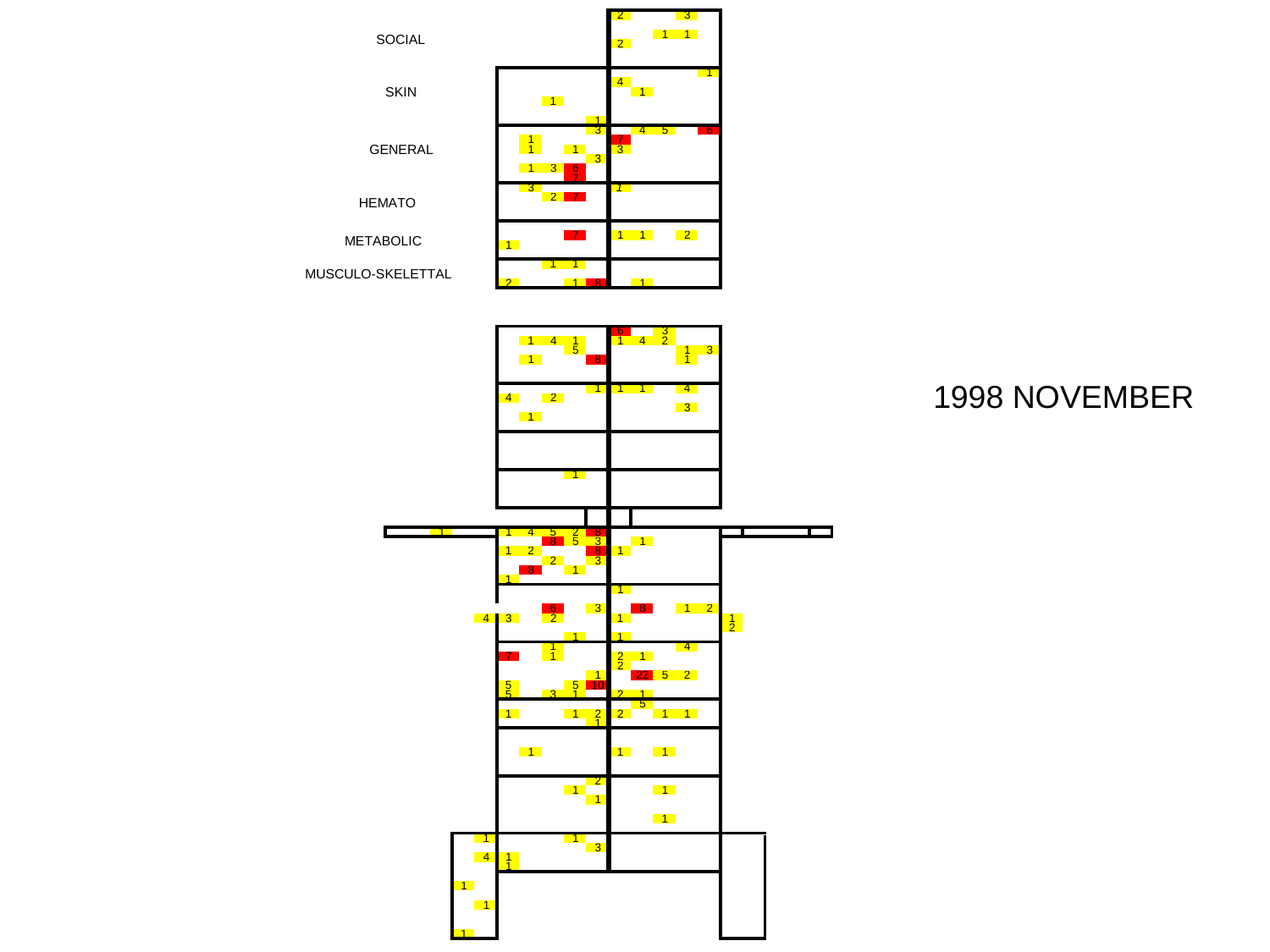

1998 NOVEMBER

## Slide 19
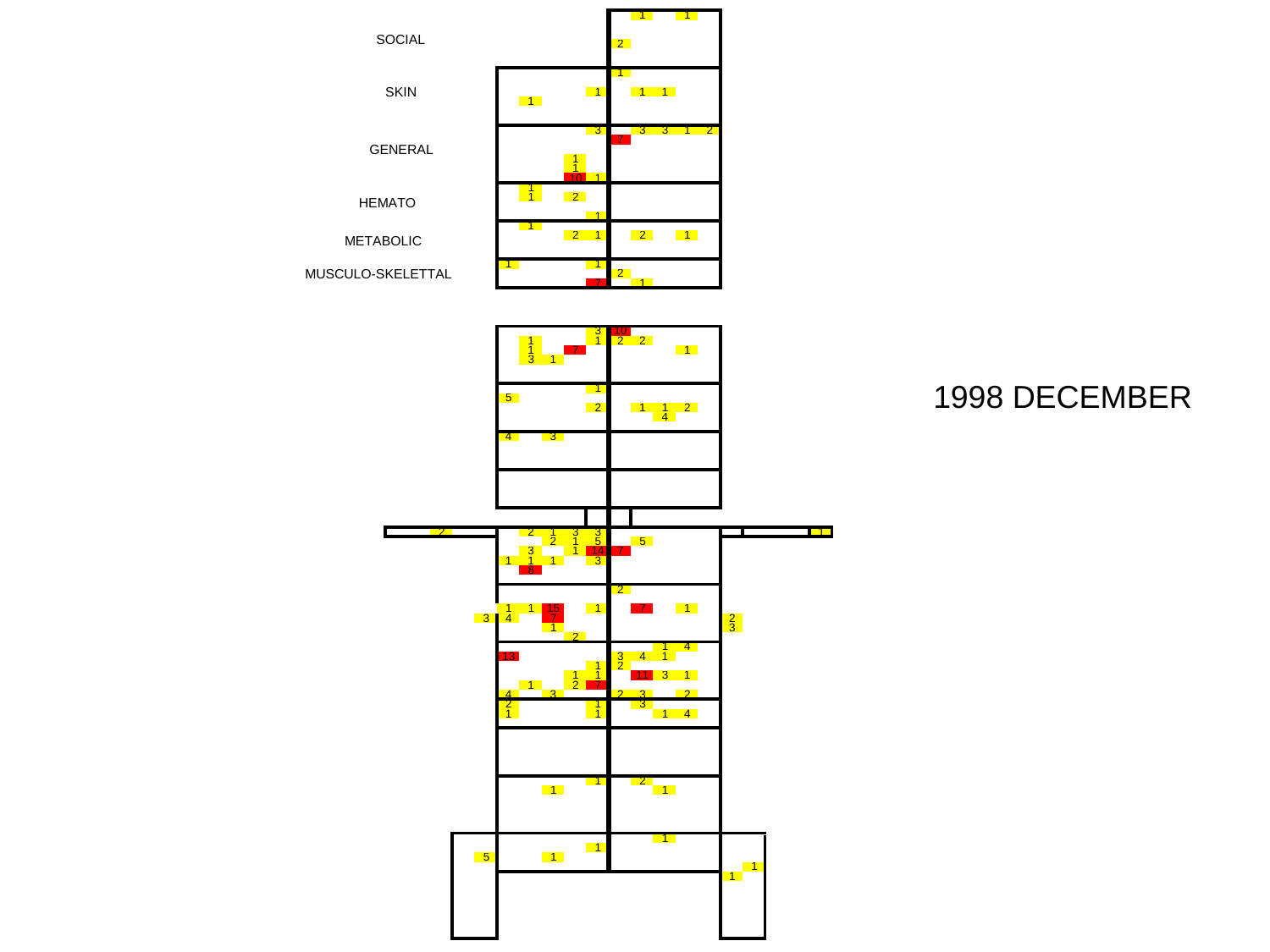

1998 DECEMBER

## Slide 20
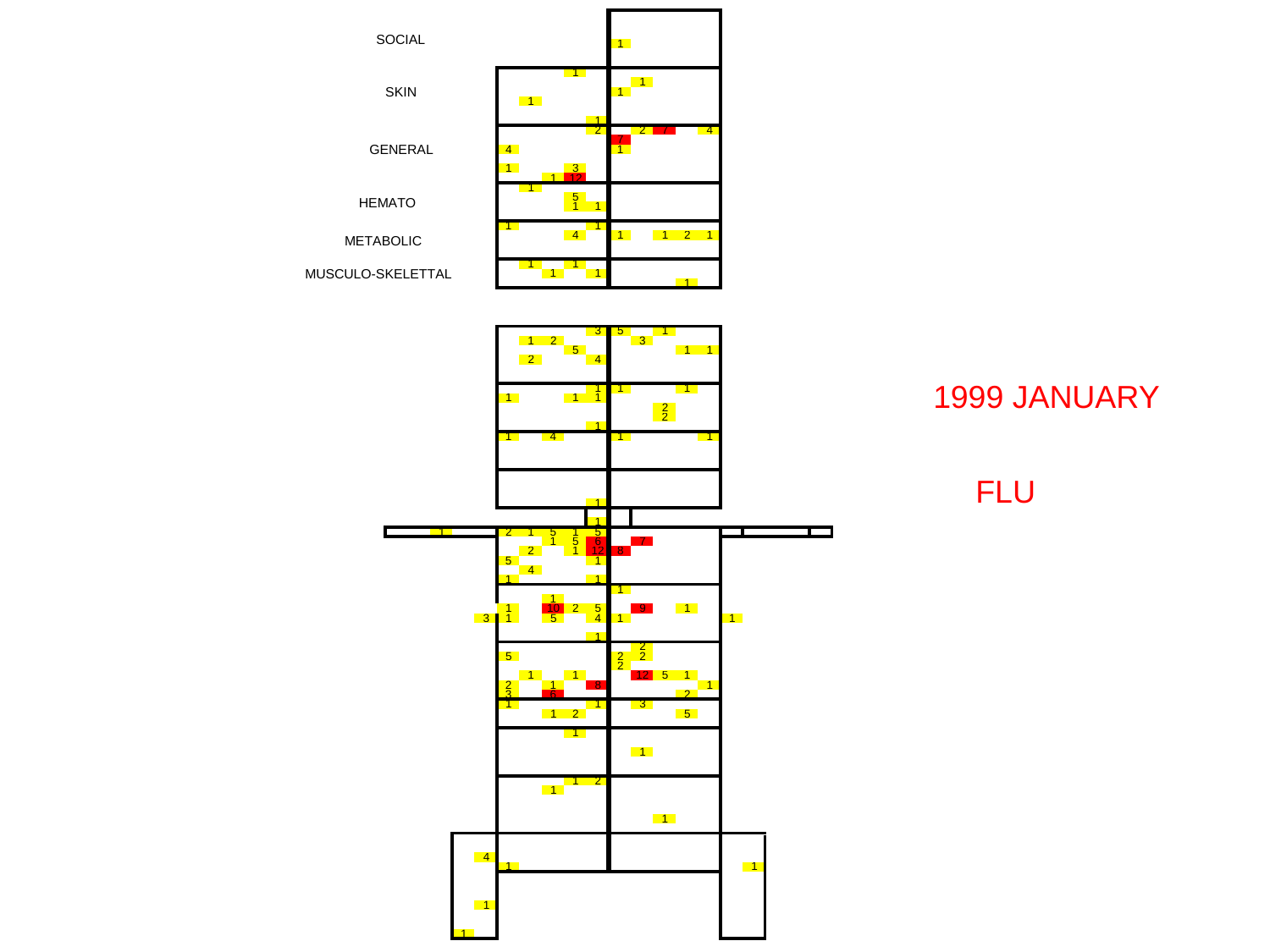

1999 JANUARY
FLU

## Slide 21
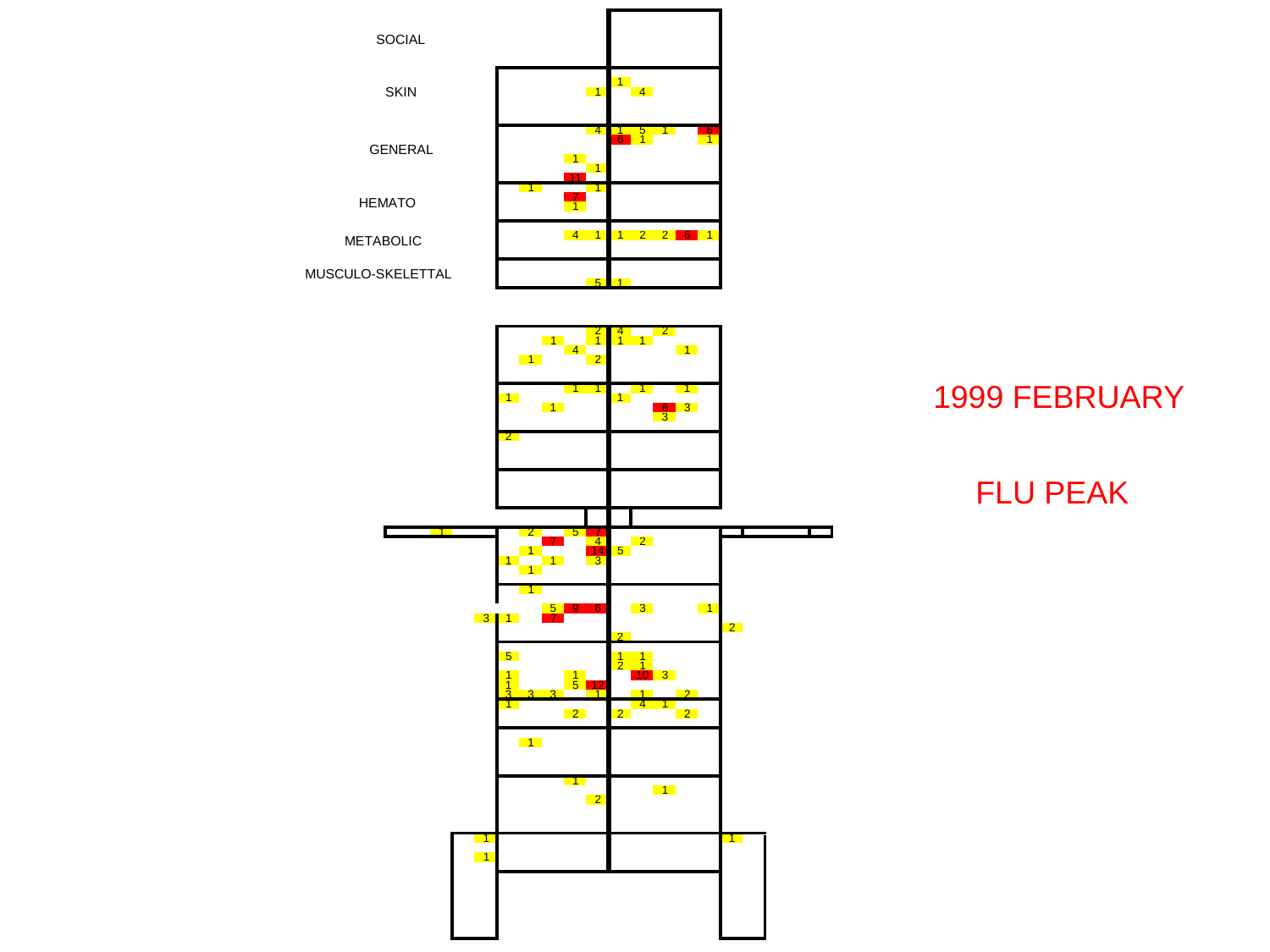

1999 FEBRUARY
FLU PEAK

## Slide 22
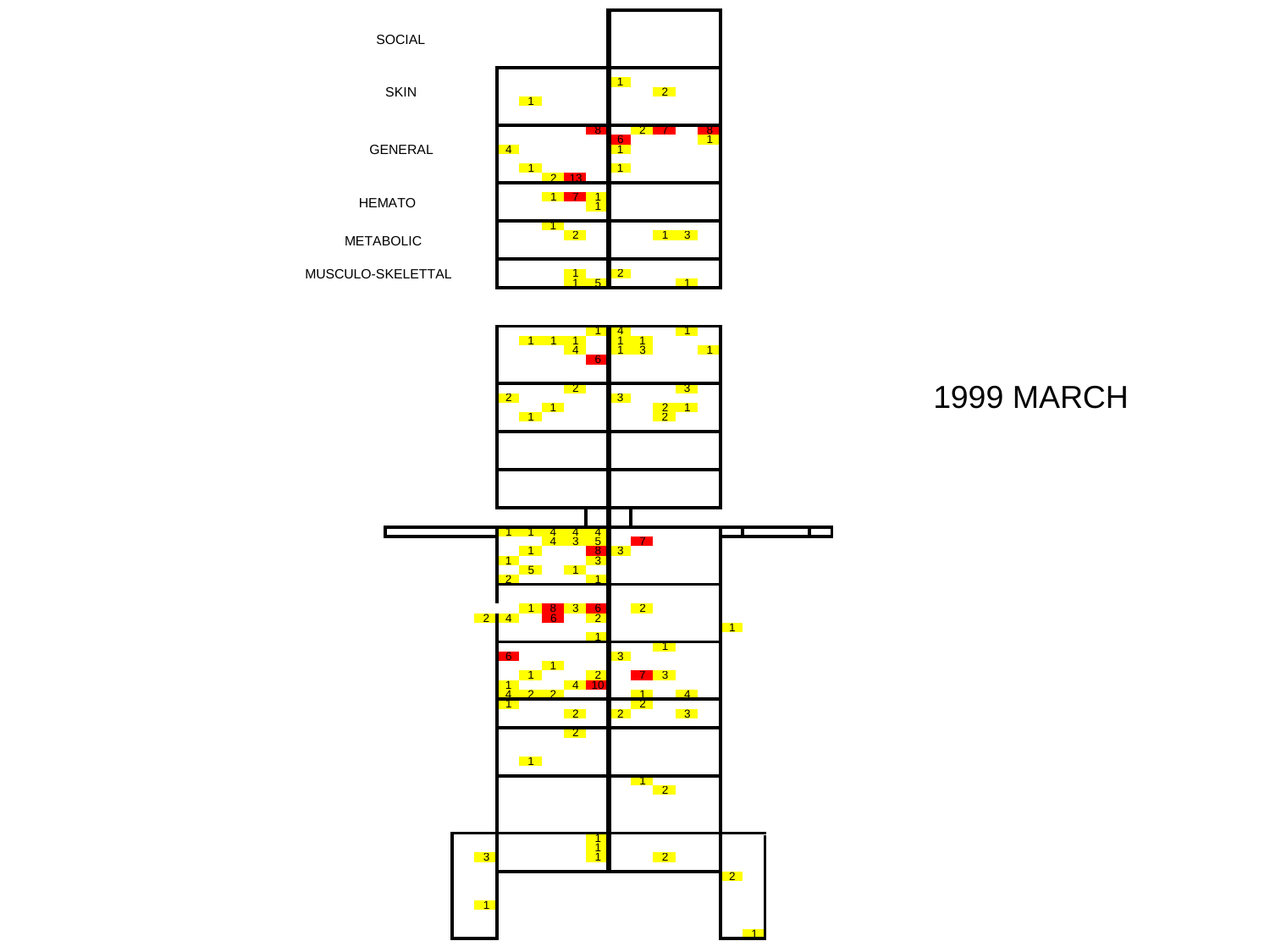

1999 MARCH

## Slide 23
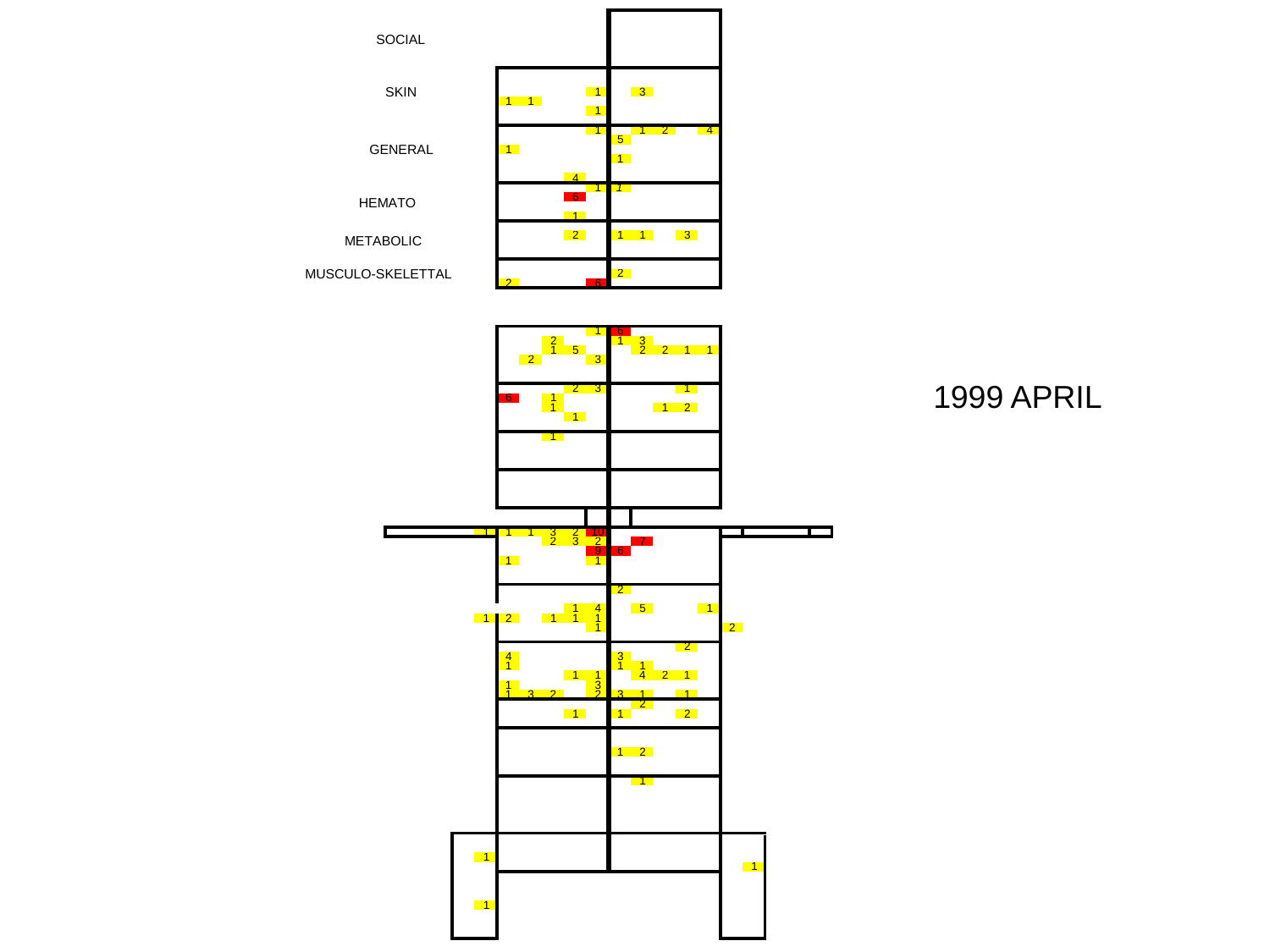

1999 APRIL

## Slide 24
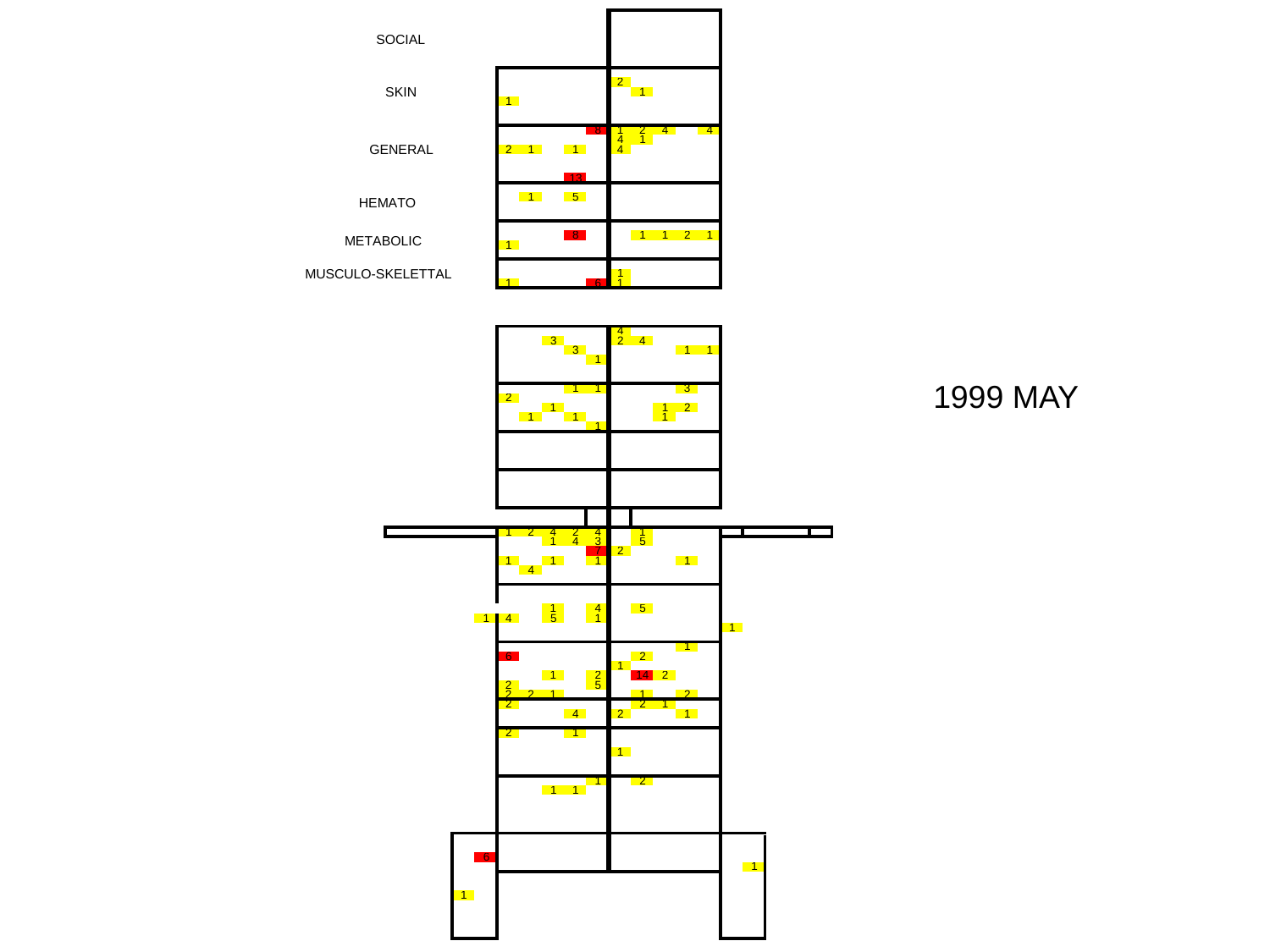

1999 MAY

## Slide 25
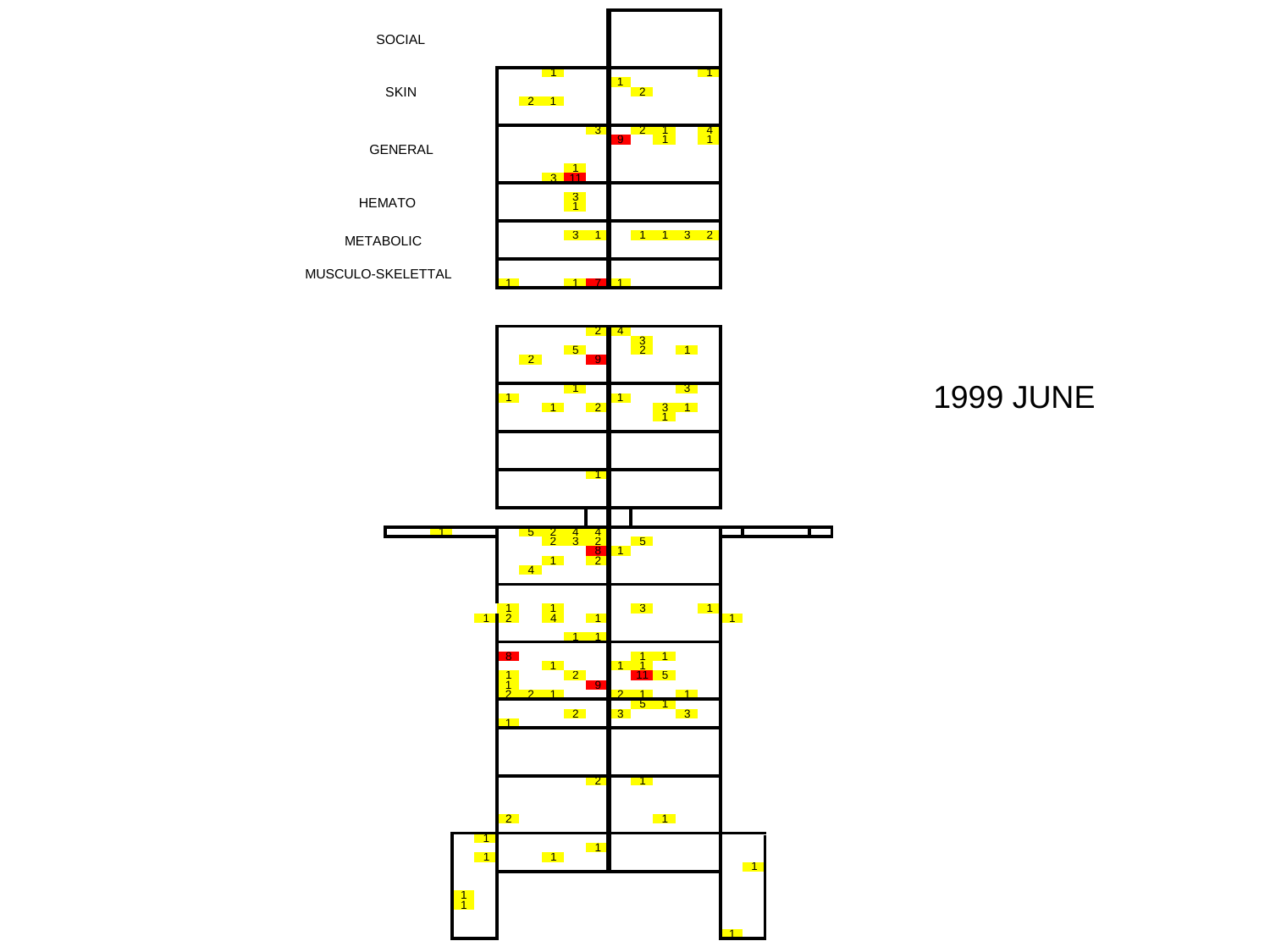

1999 JUNE

## Slide 26
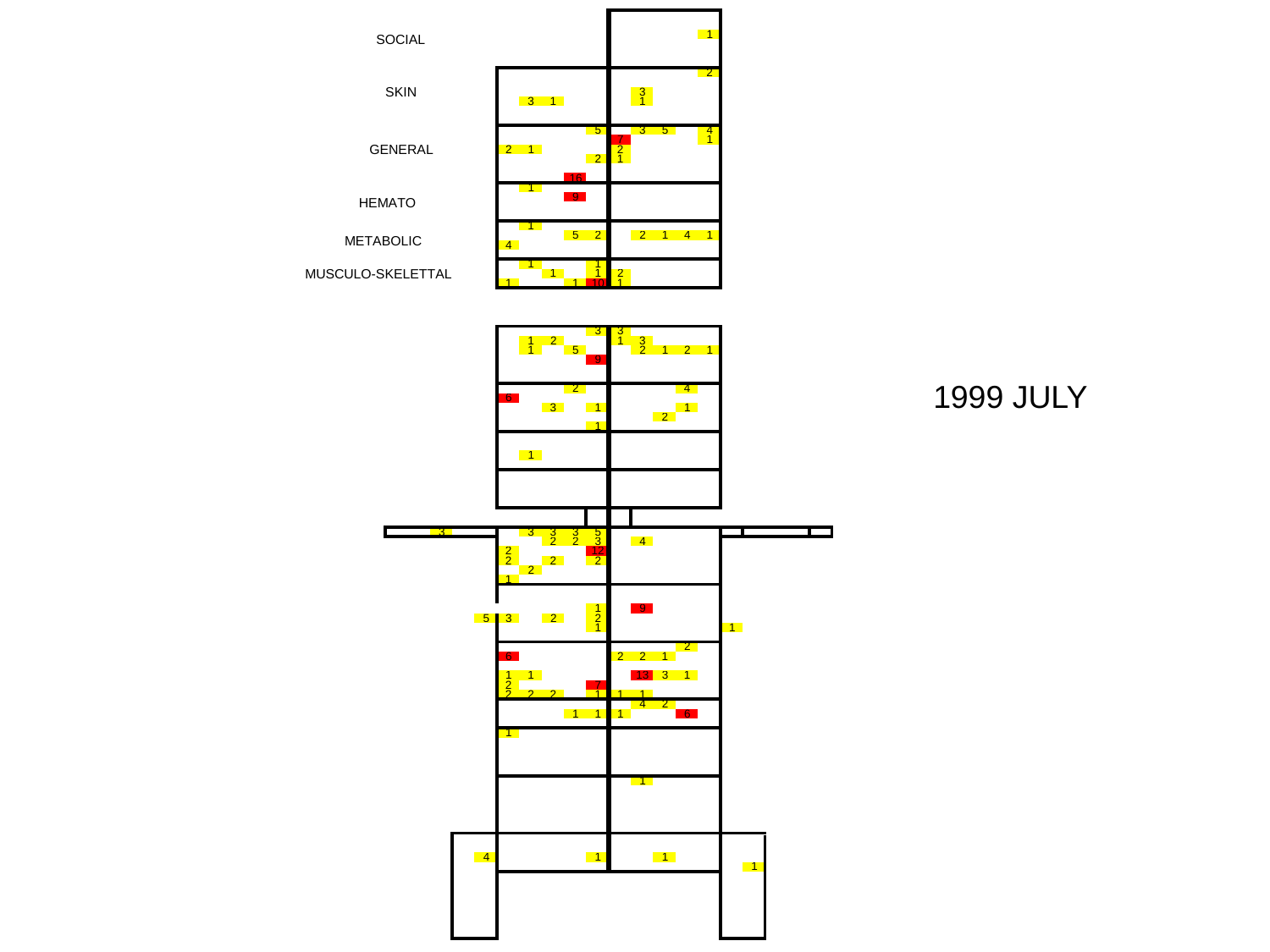

1999 JULY

## Slide 27
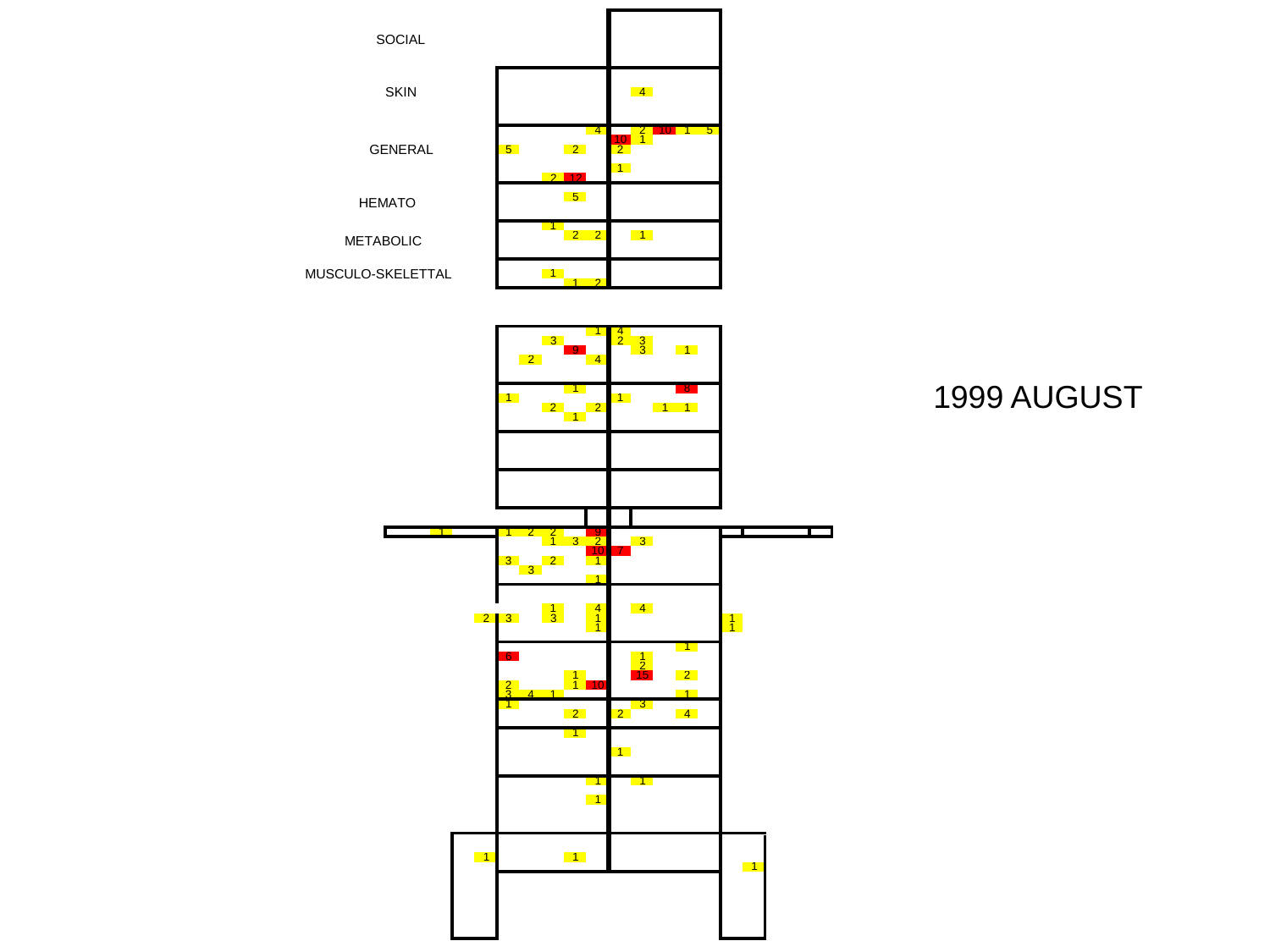

1999 AUGUST

## Slide 28
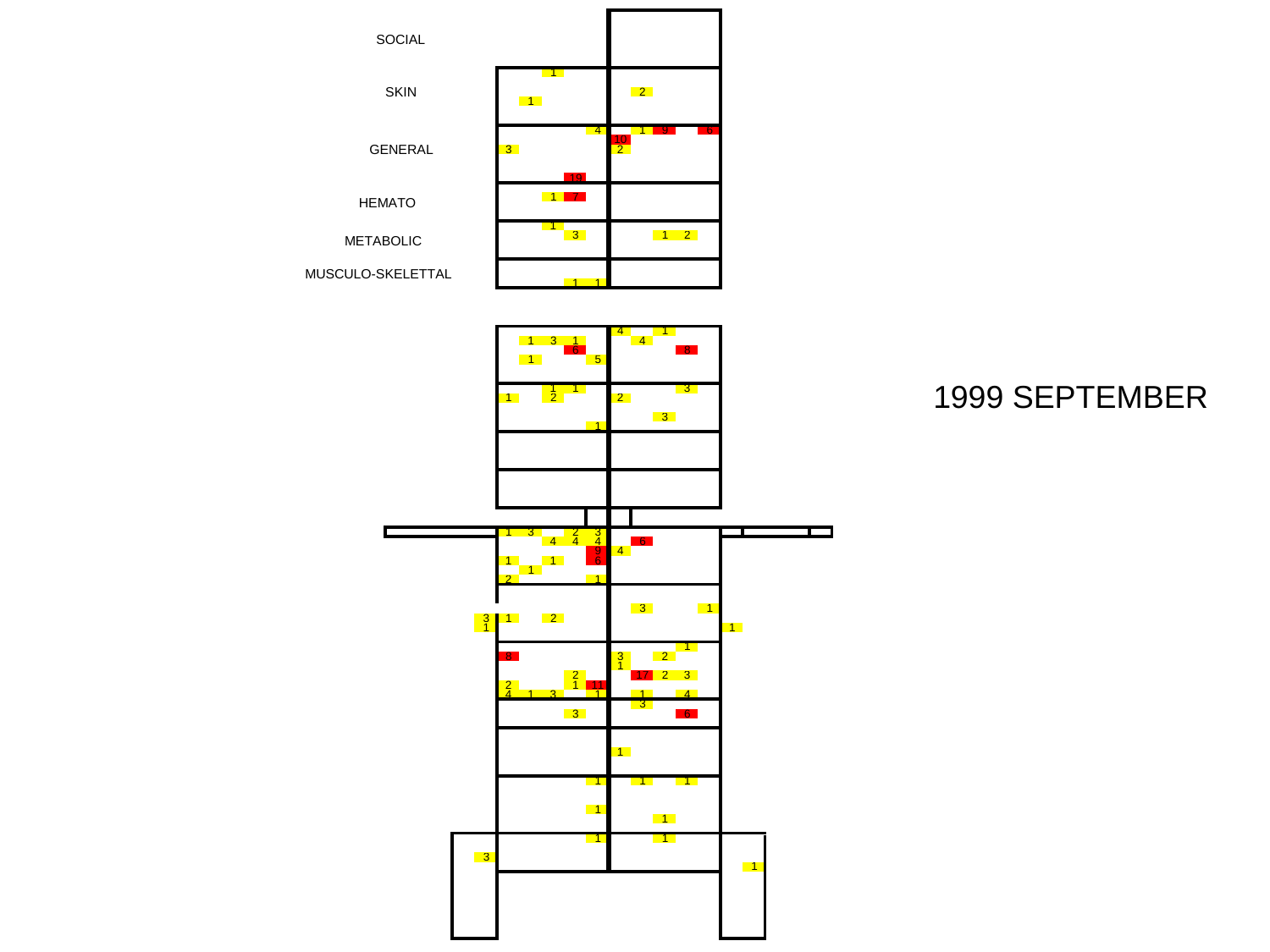

1999 SEPTEMBER

## Slide 29
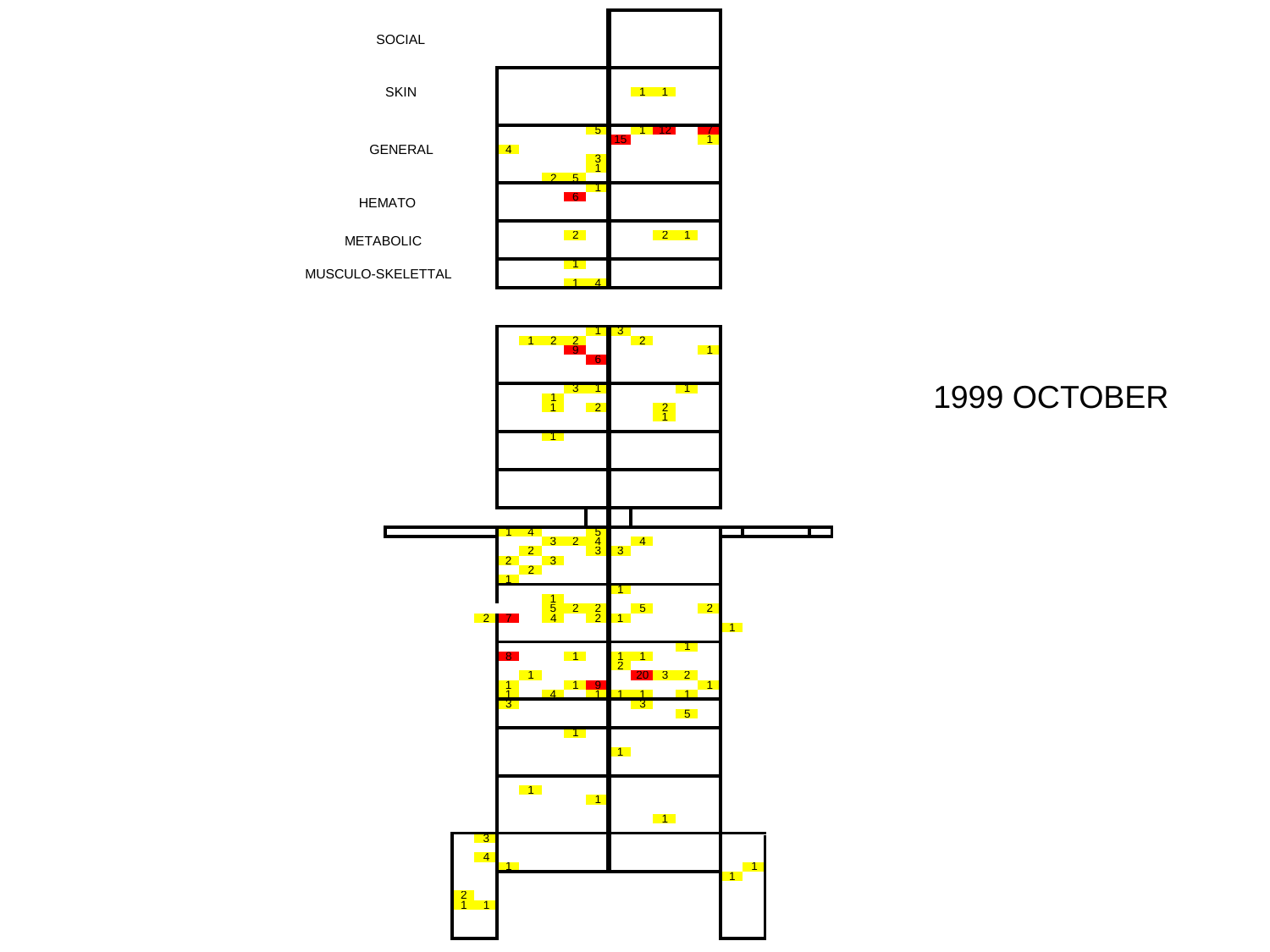

1999 OCTOBER

## Slide 30
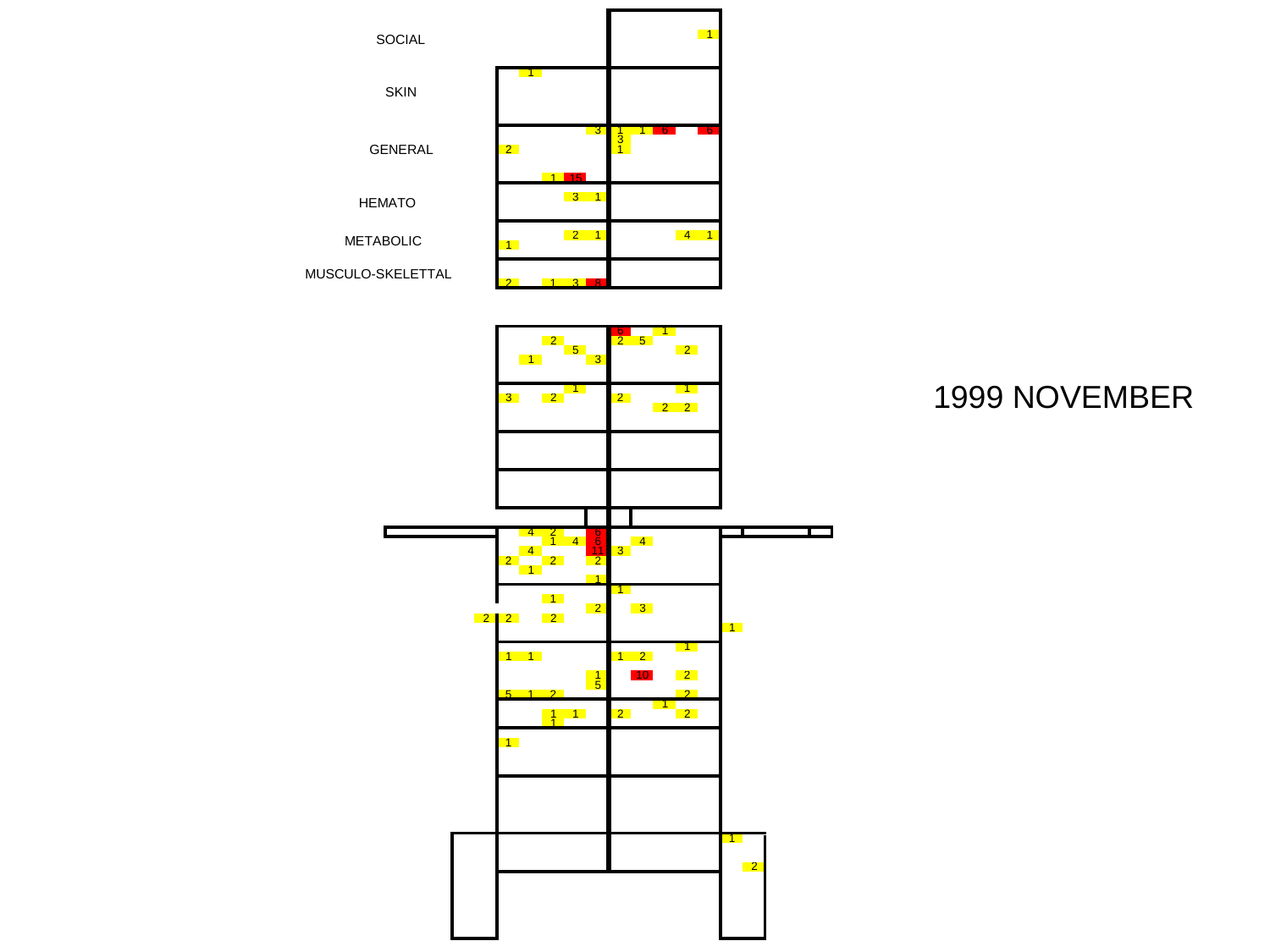

1999 NOVEMBER

## Slide 31
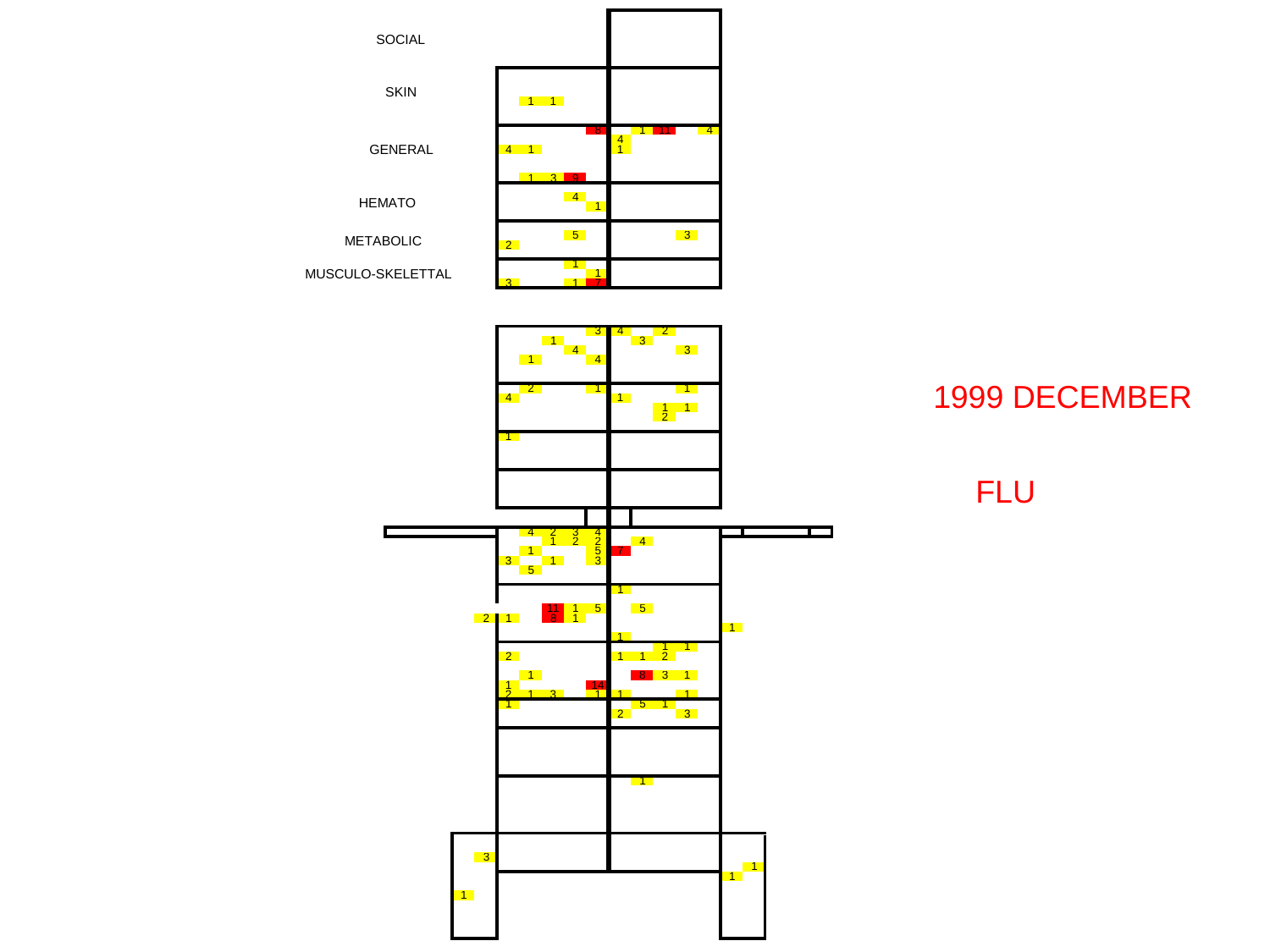

1999 DECEMBER
FLU

## Slide 32
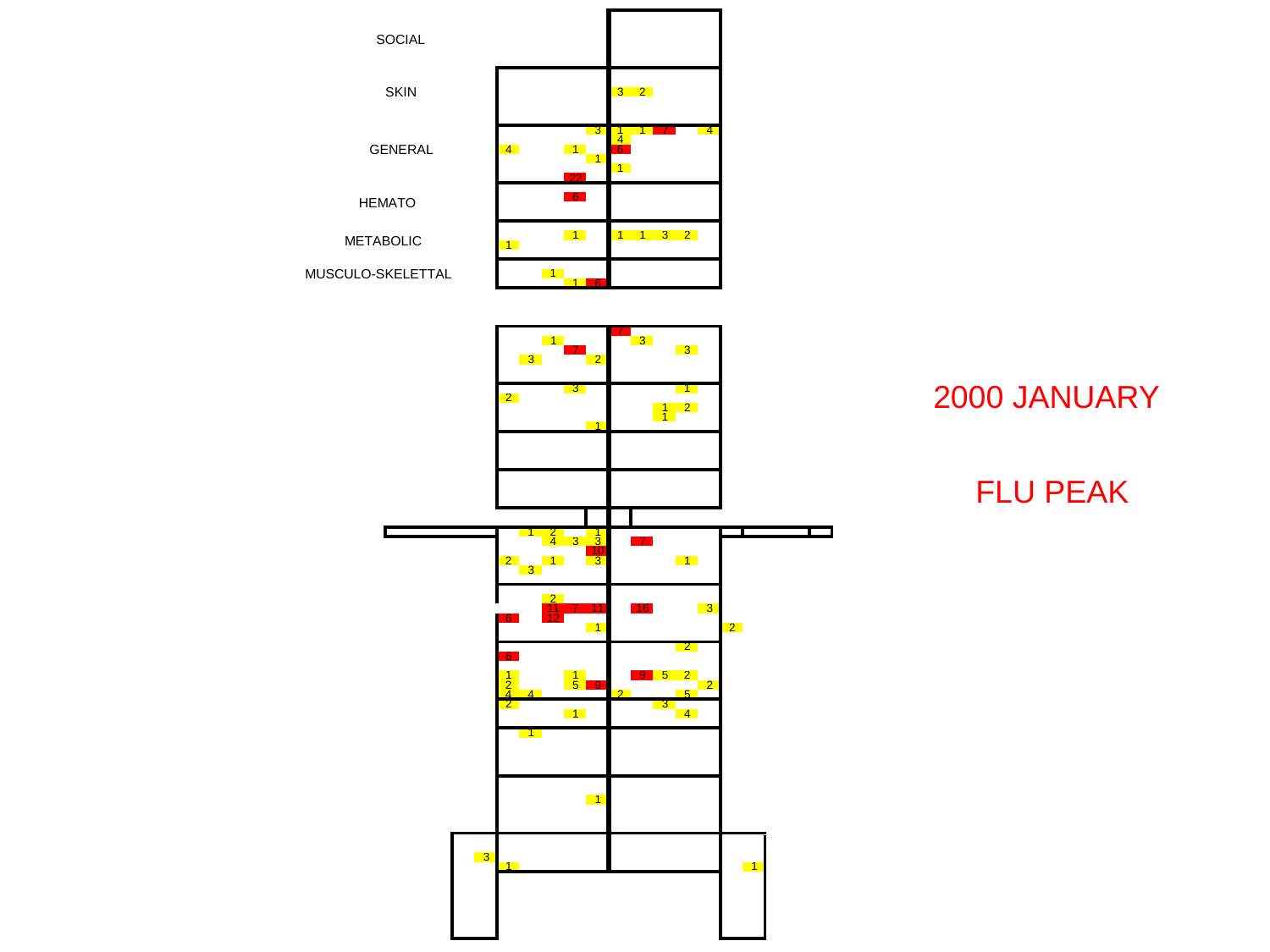

2000 JANUARY
FLU PEAK

## Slide 33
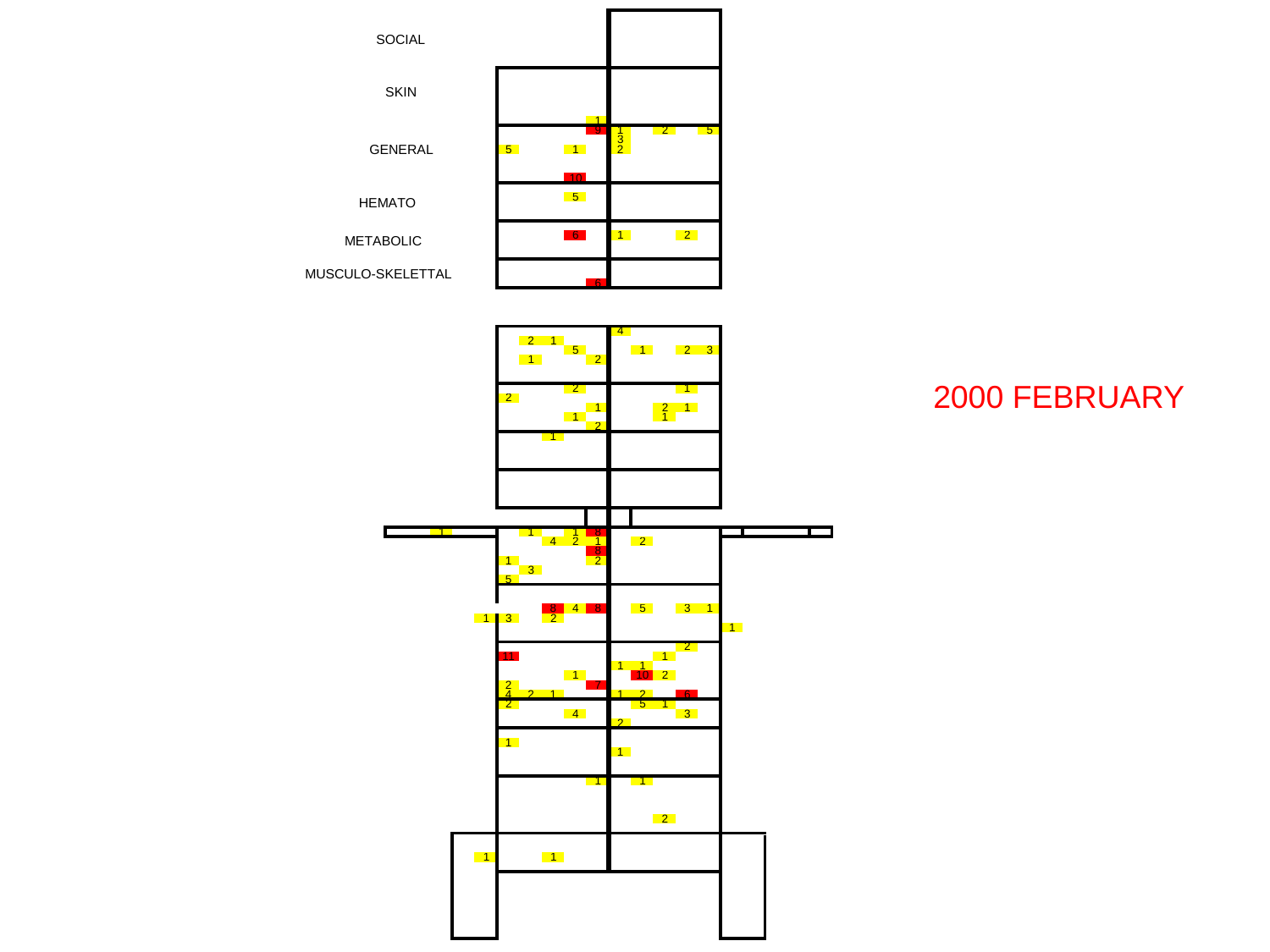

2000 FEBRUARY

## Slide 34
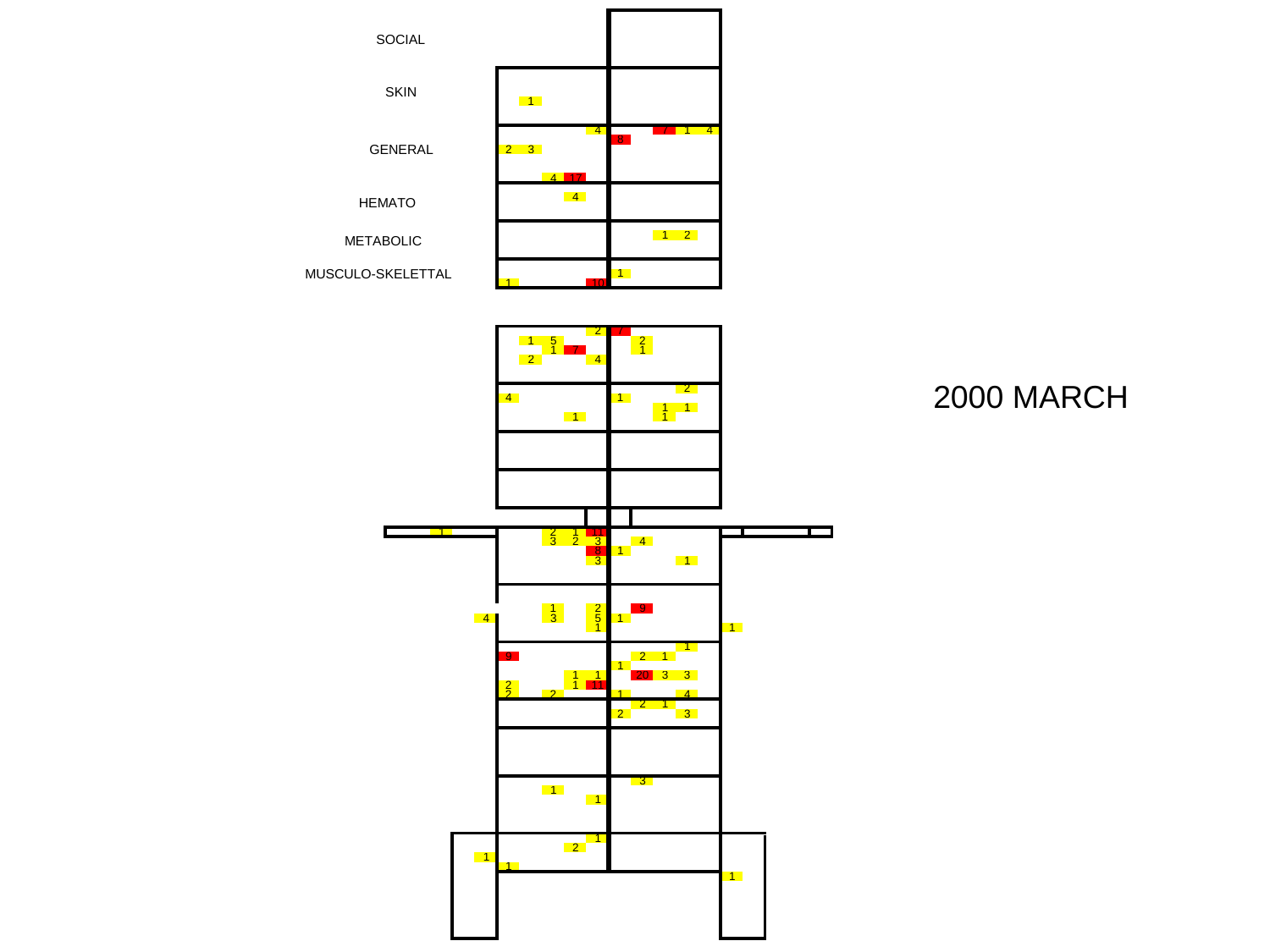

2000 MARCH

## Slide 35
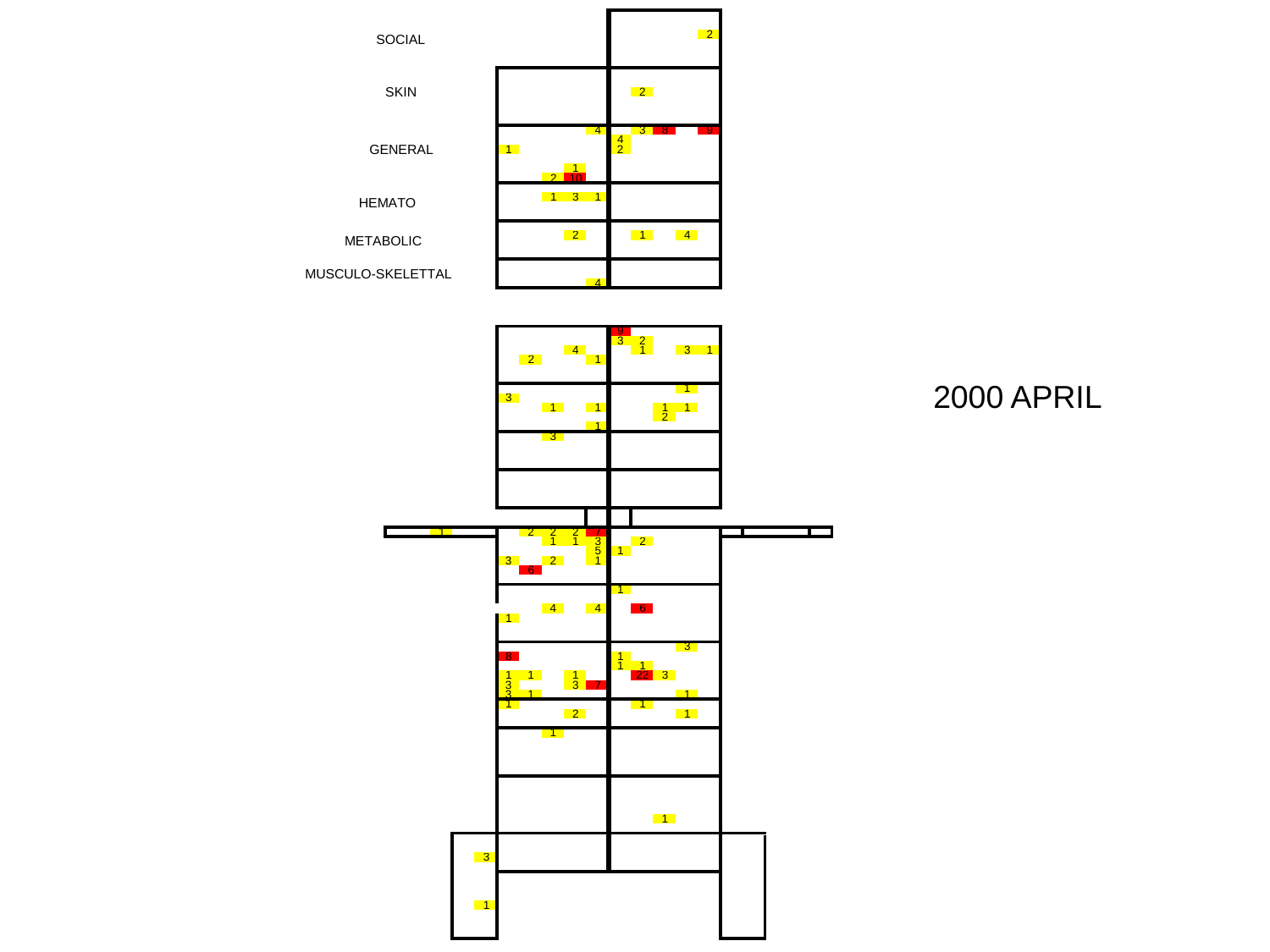

2000 APRIL

## Slide 36
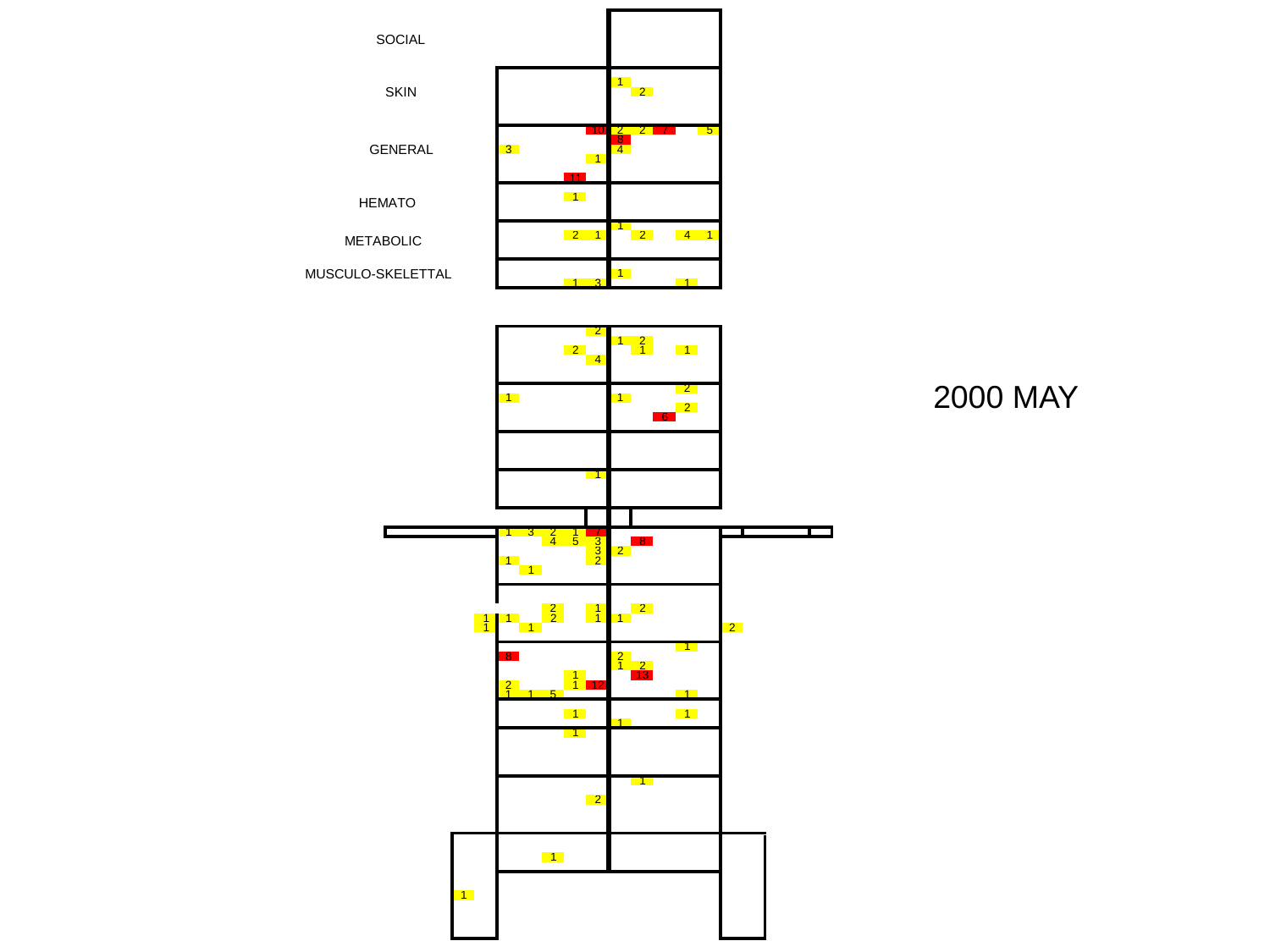

2000 MAY

## Slide 37
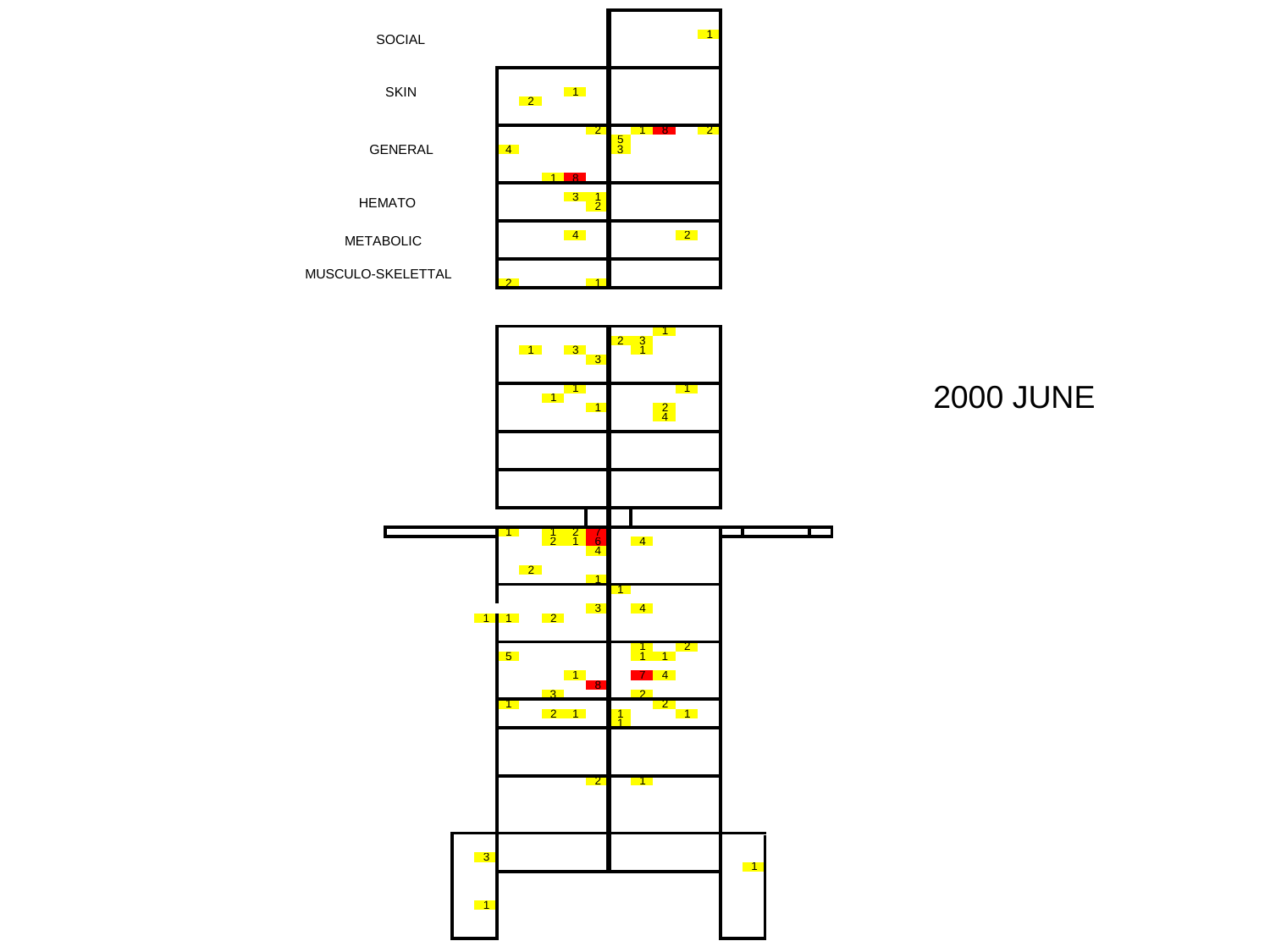

2000 JUNE

## Slide 38
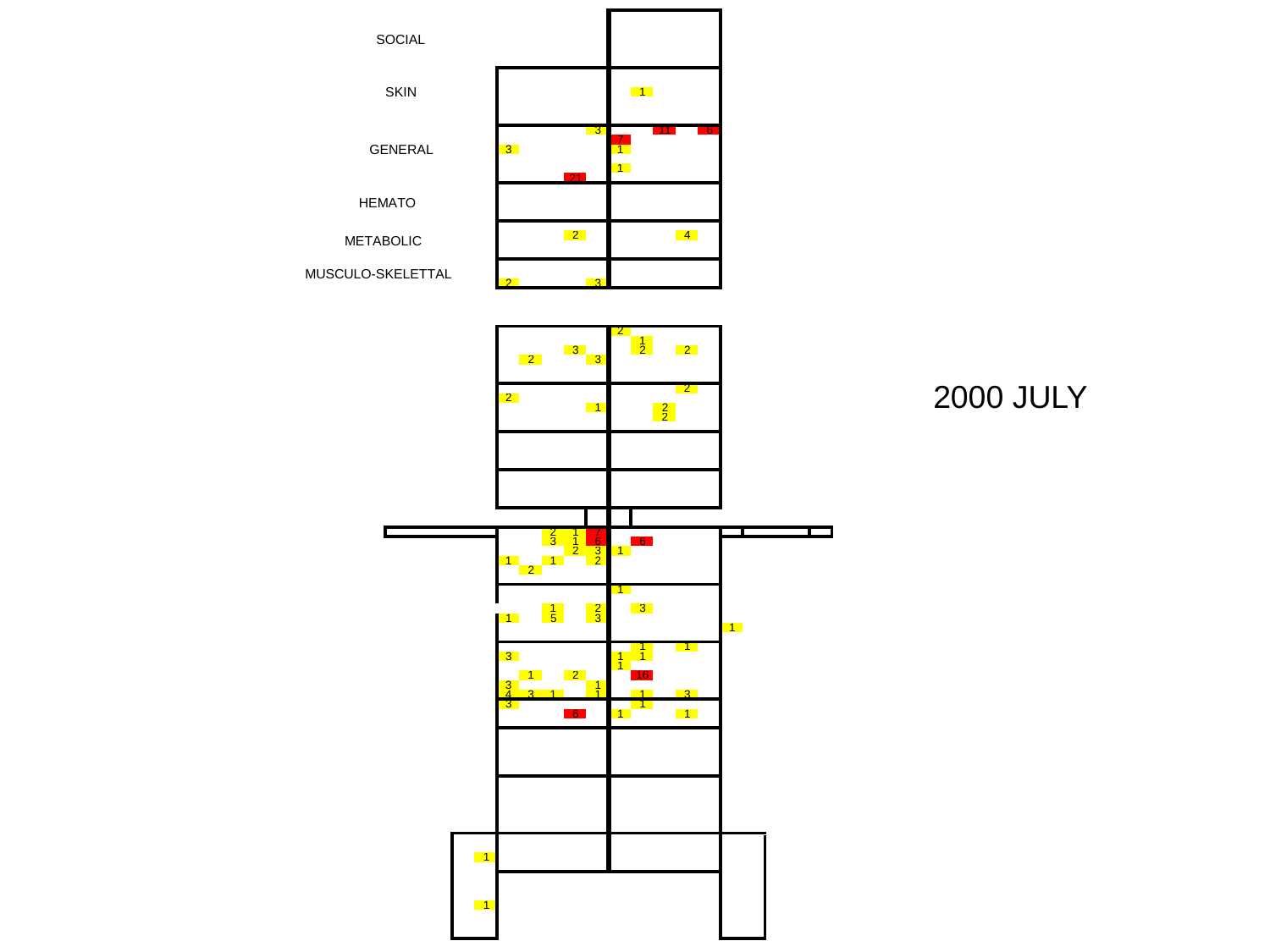

2000 JULY

## Slide 39
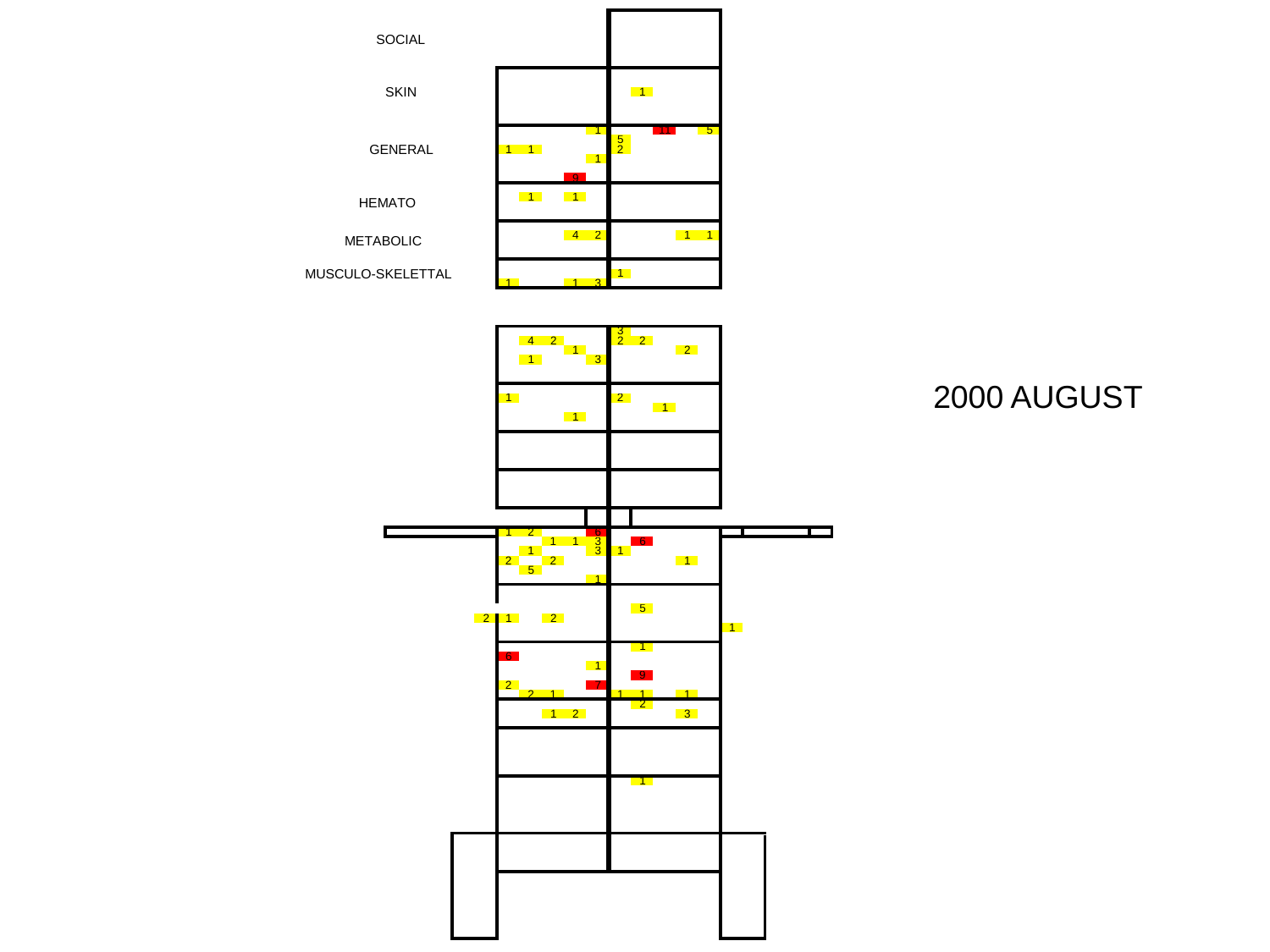

2000 AUGUST

## Slide 40
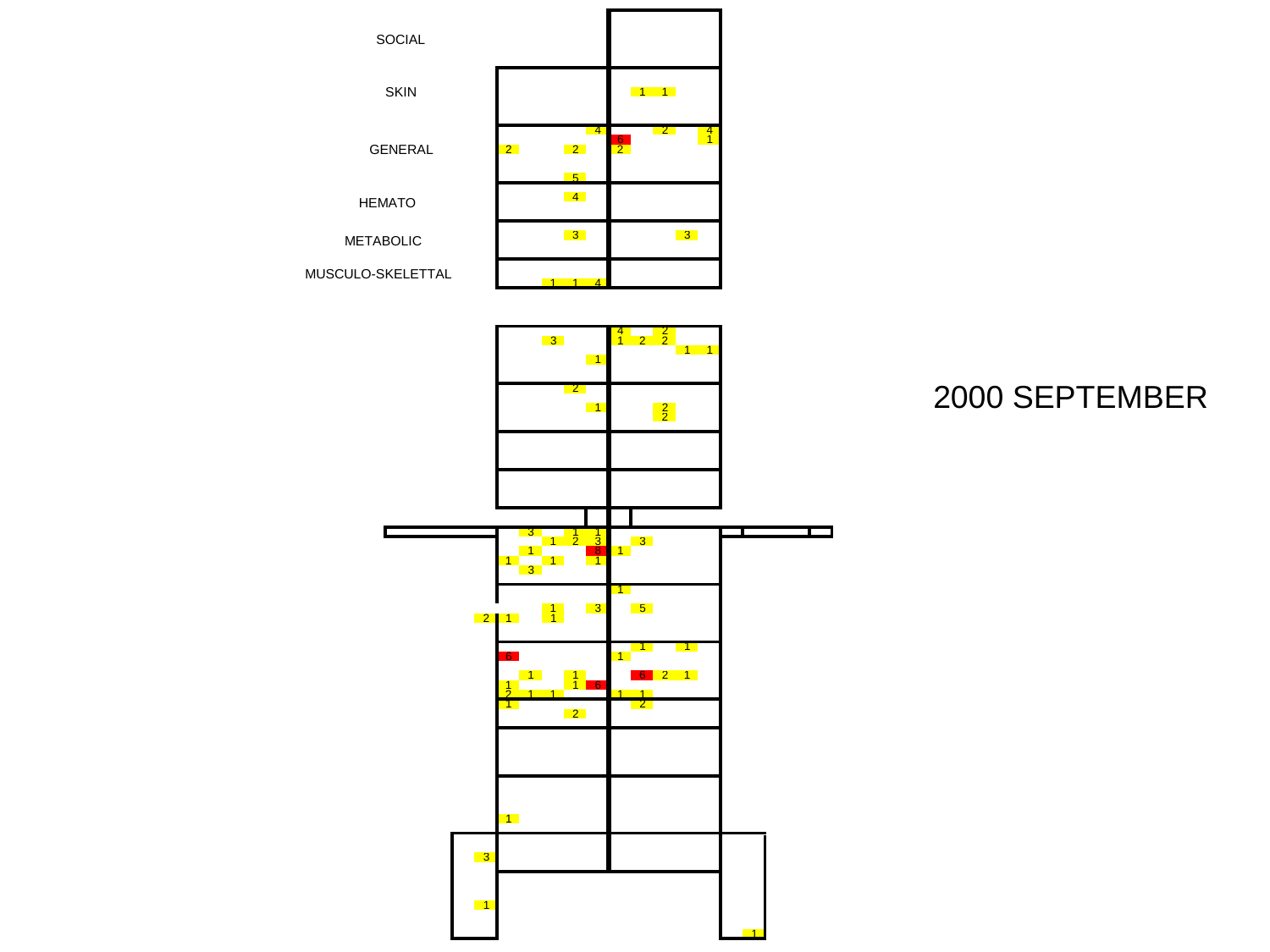

2000 SEPTEMBER

## Slide 41
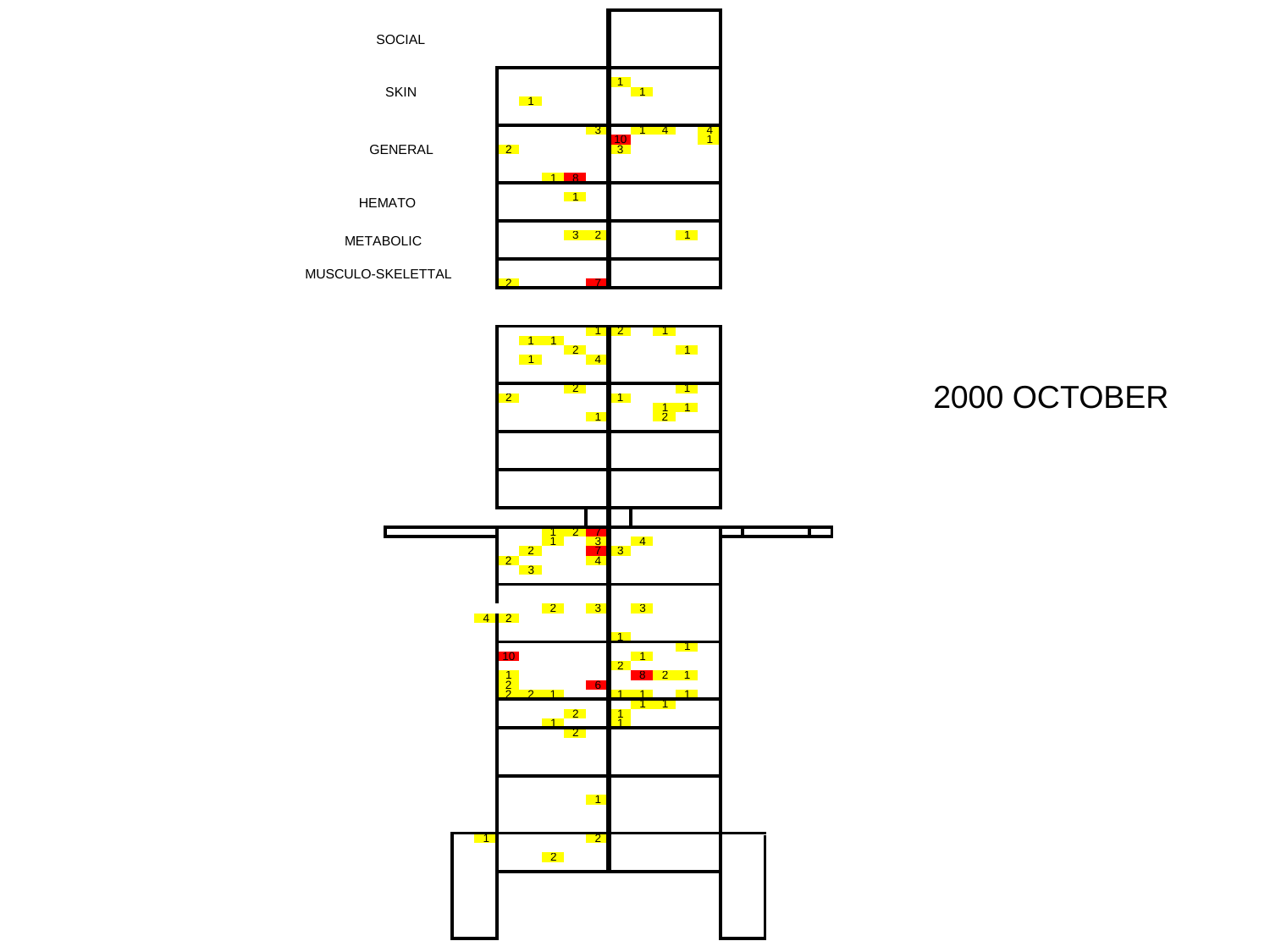

2000 OCTOBER

## Slide 42
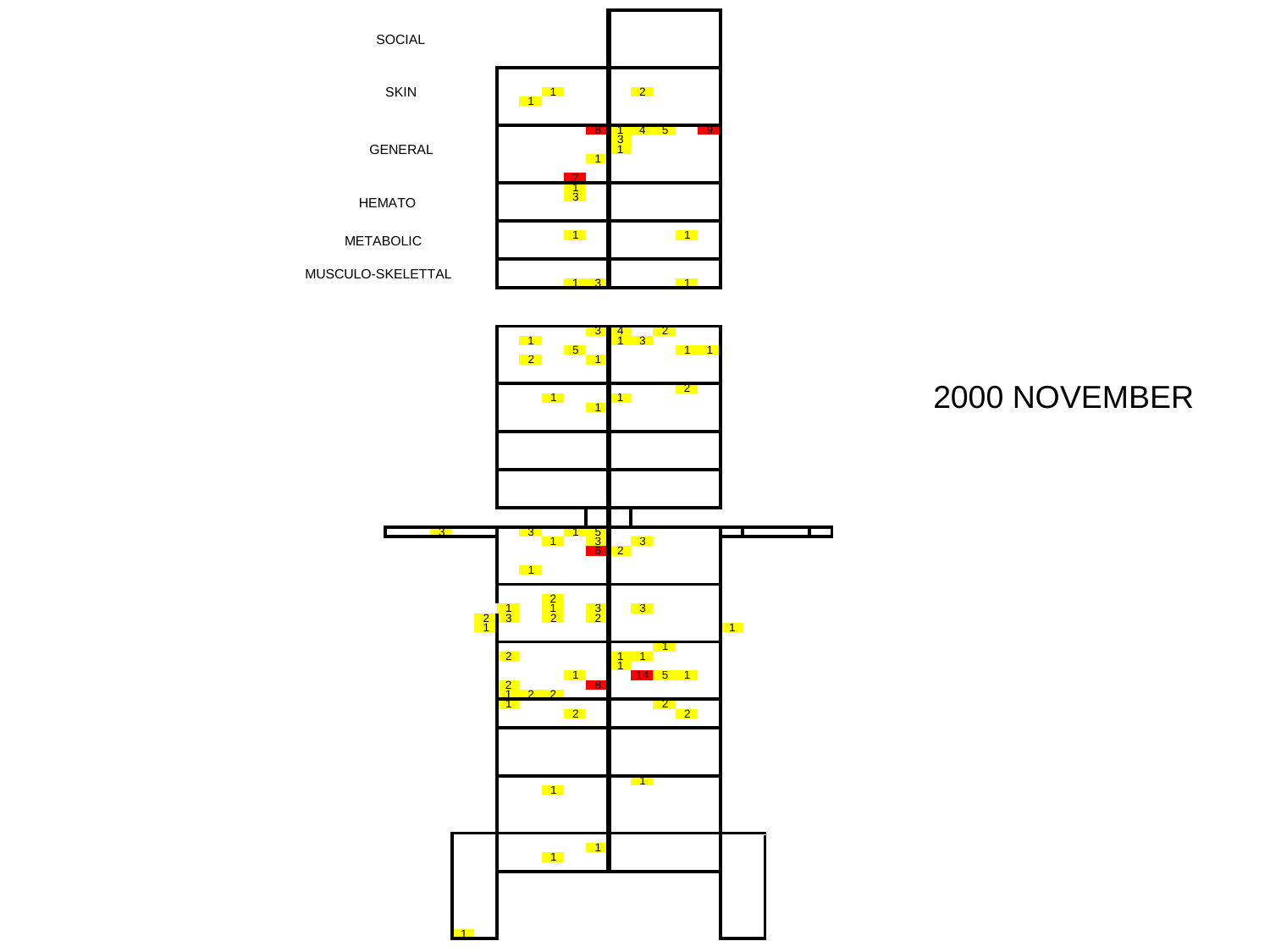

2000 NOVEMBER

## Slide 43
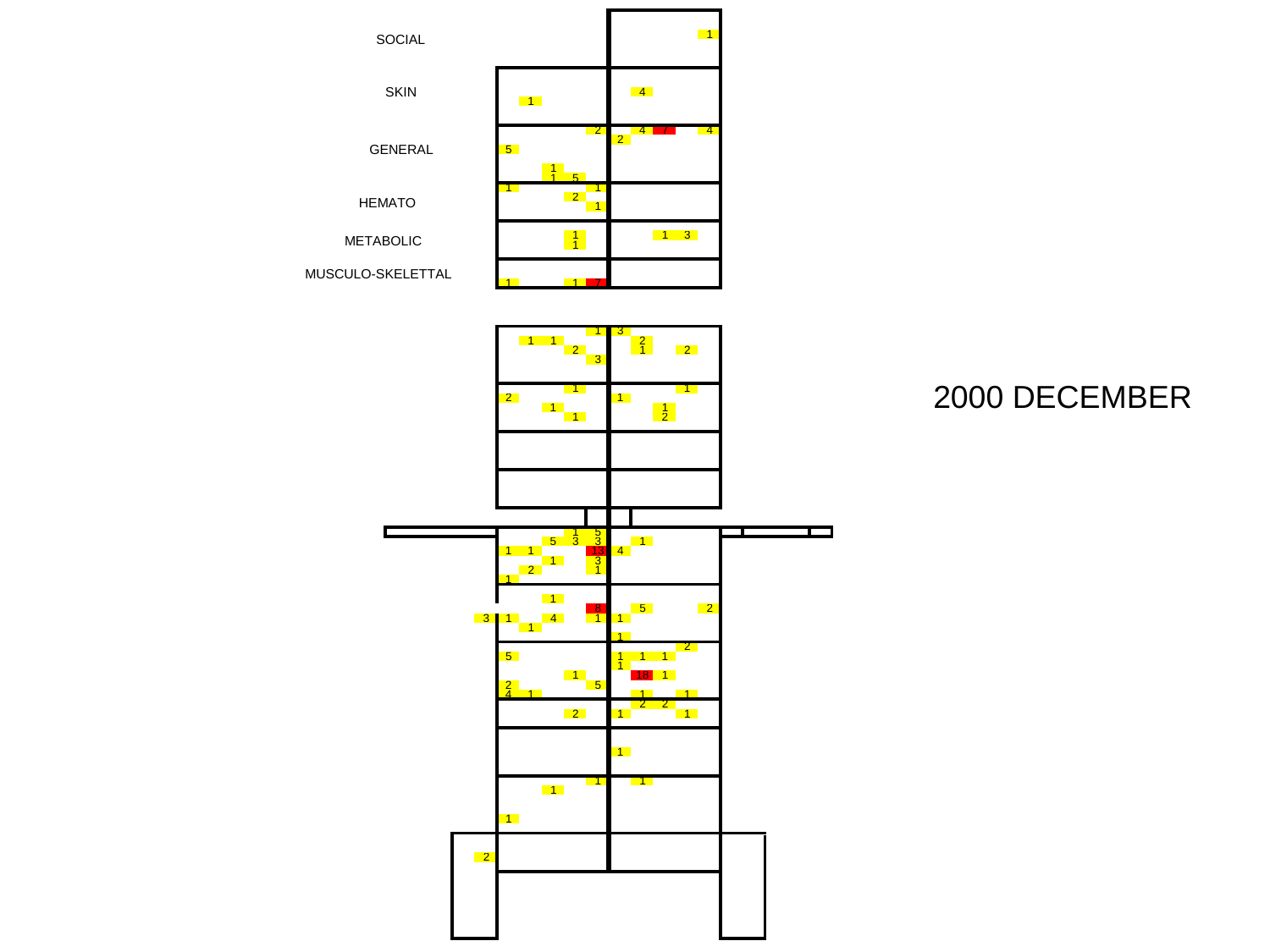

2000 DECEMBER

## Slide 44
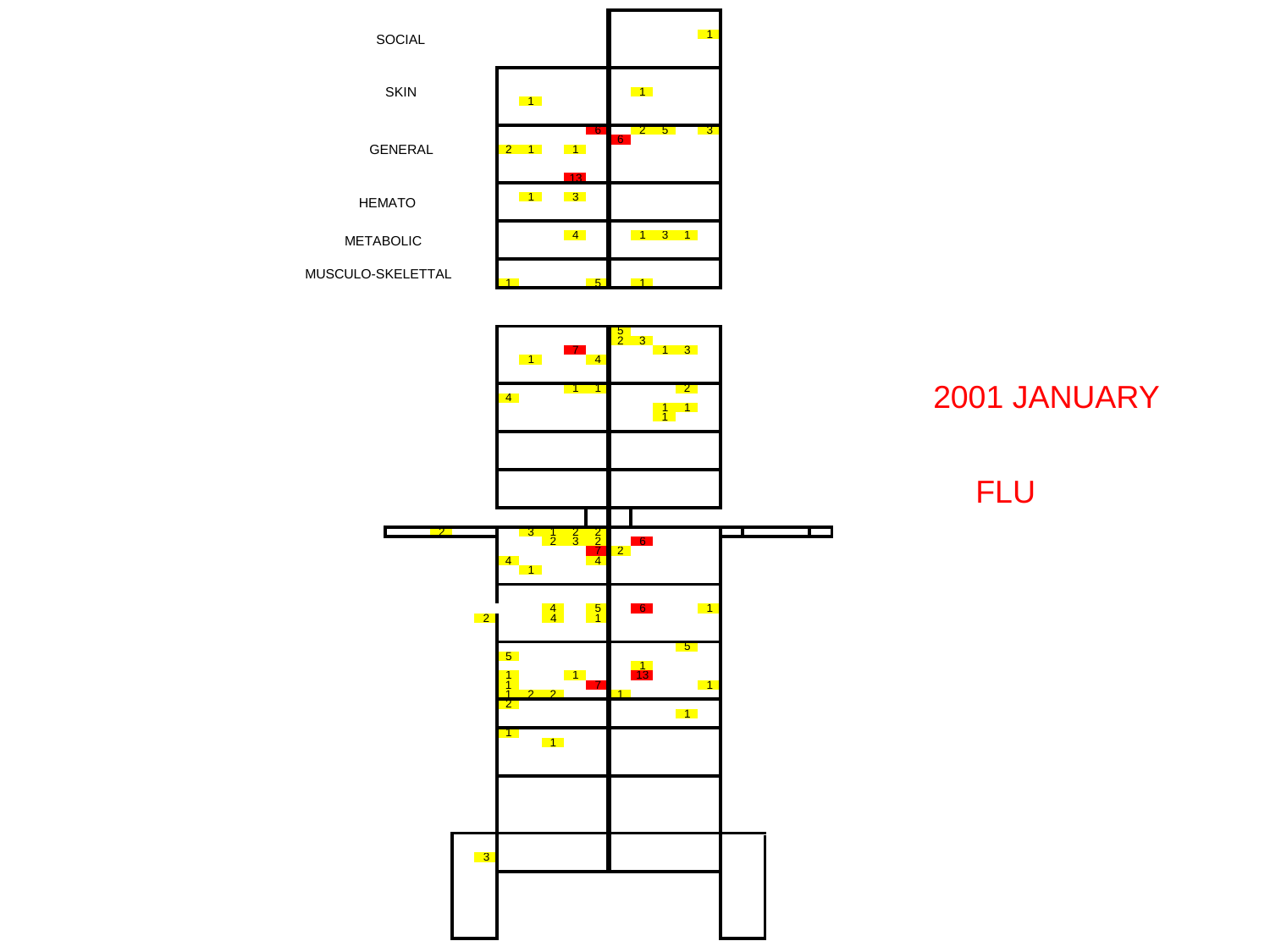

2001 JANUARY
FLU

## Slide 45
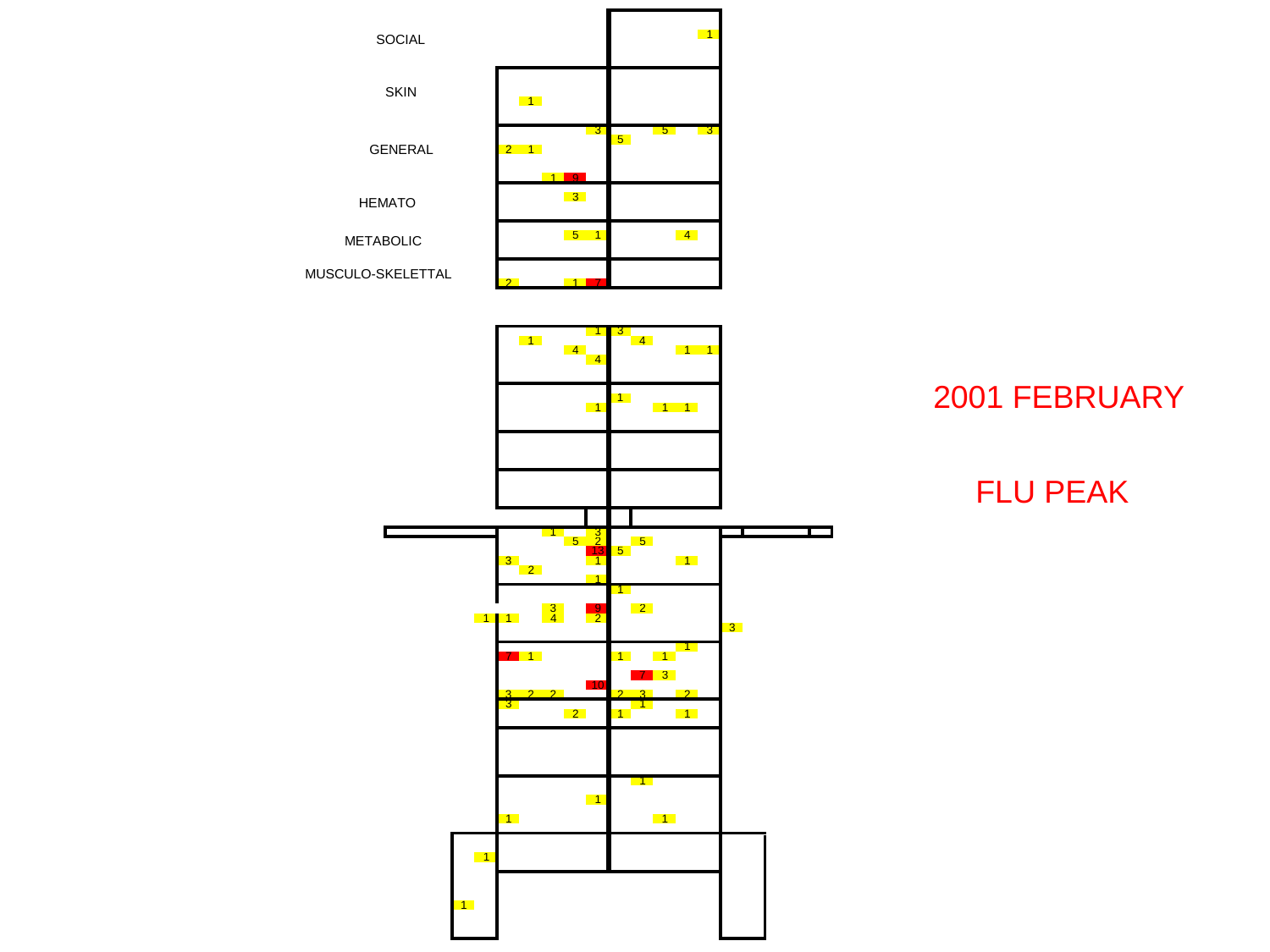

2001 FEBRUARY
FLU PEAK

## Slide 46
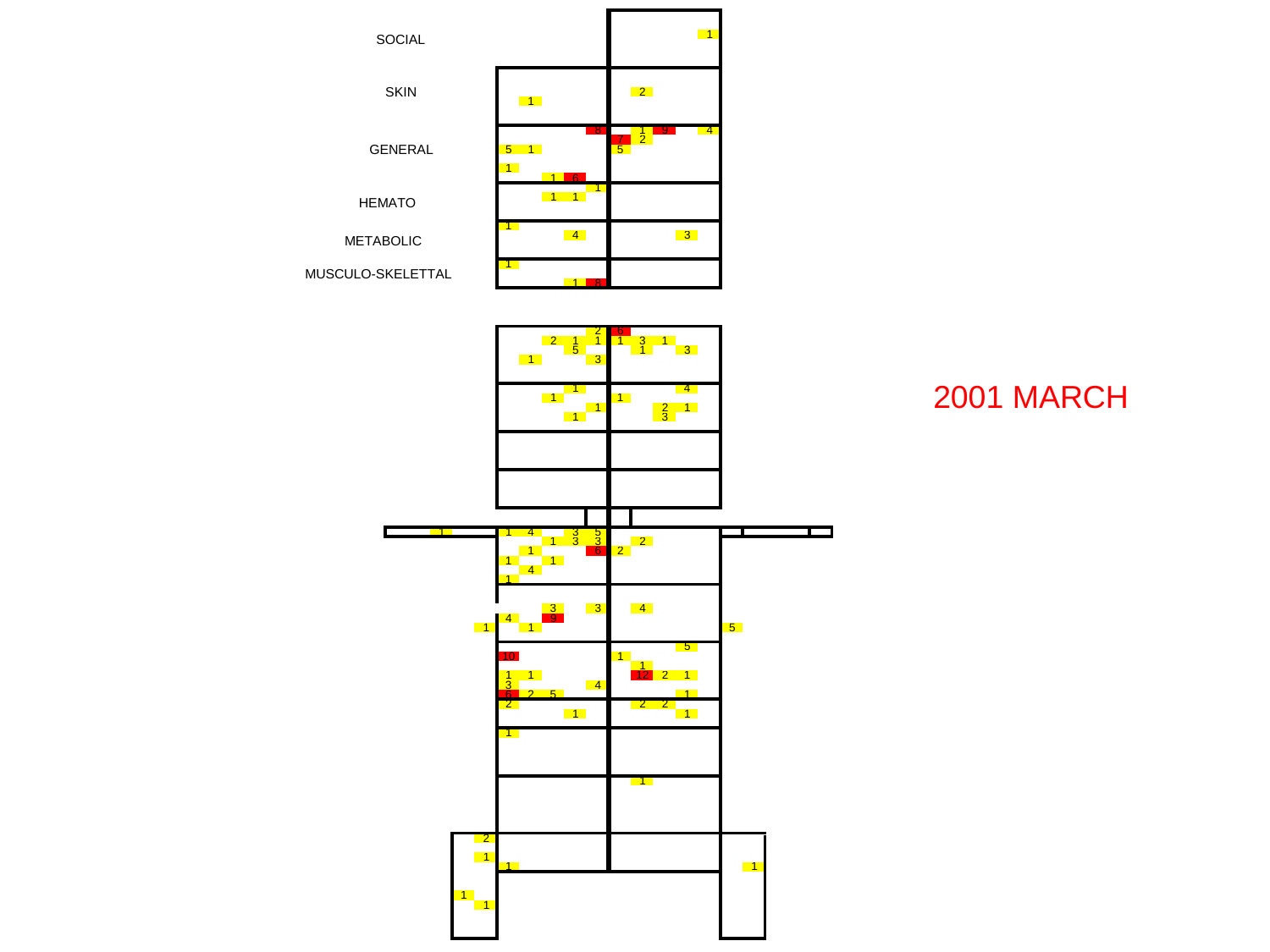

2001 MARCH

## Slide 47
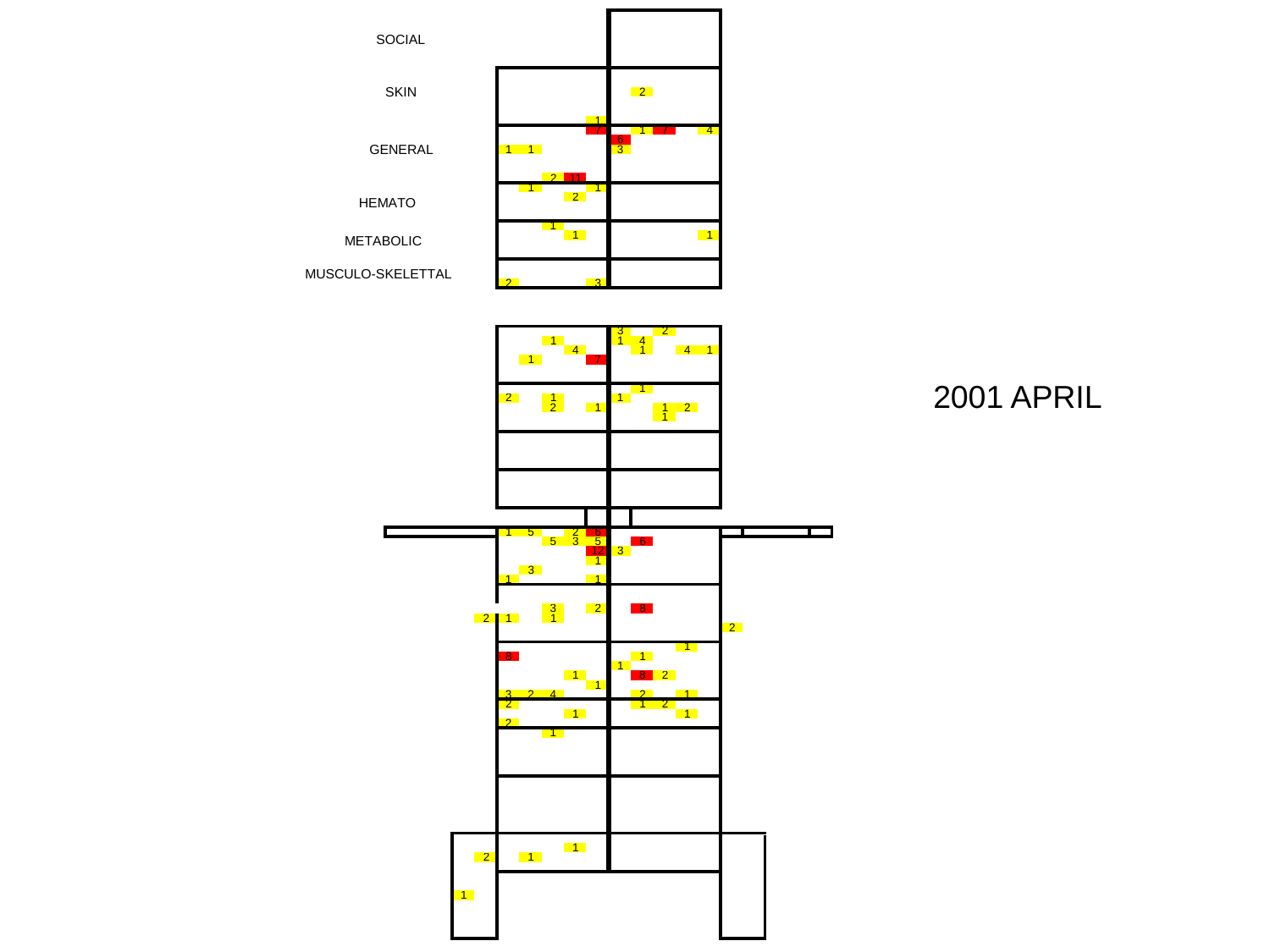

2001 APRIL

## Slide 48
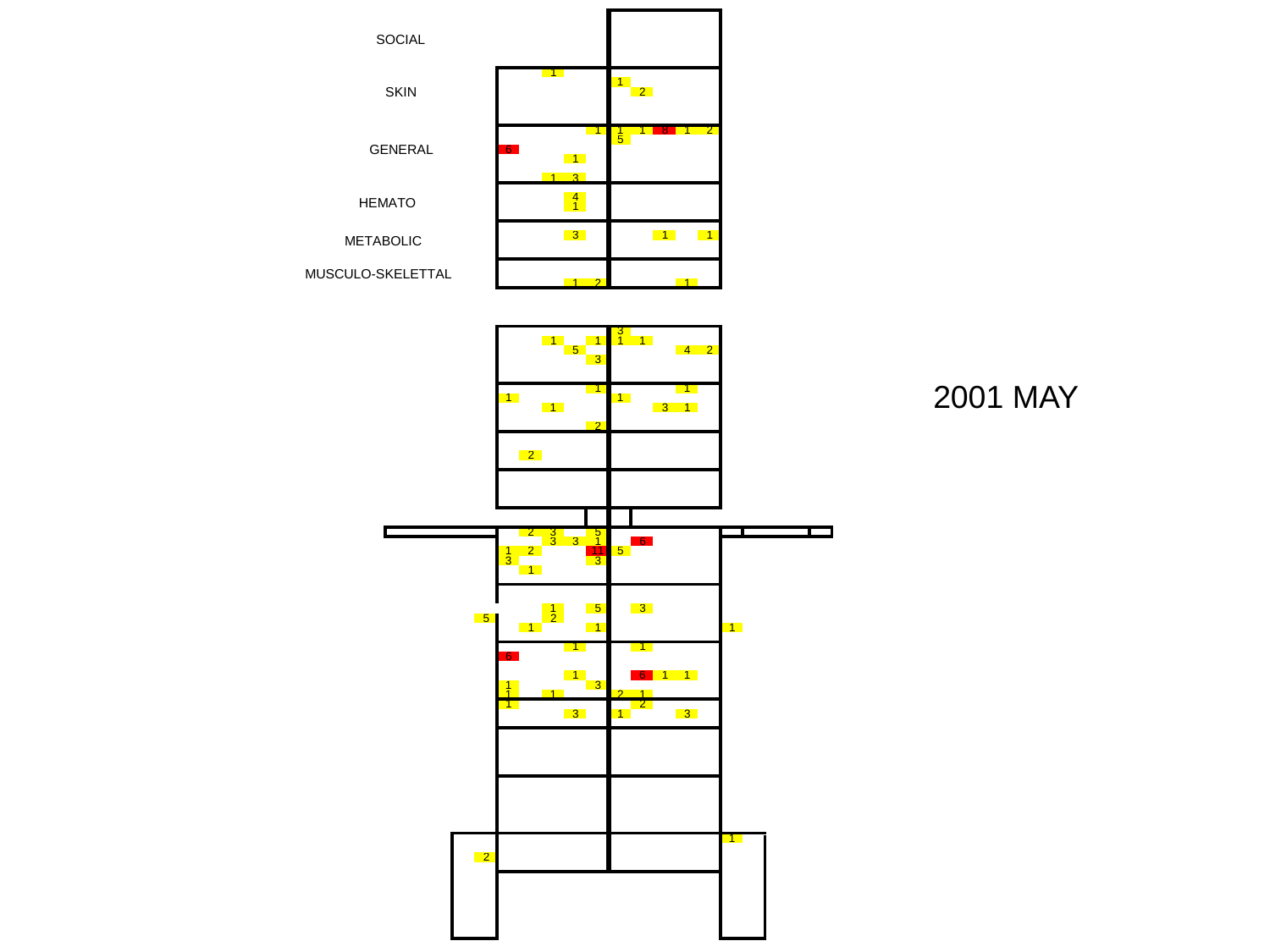

2001 MAY

## Slide 49
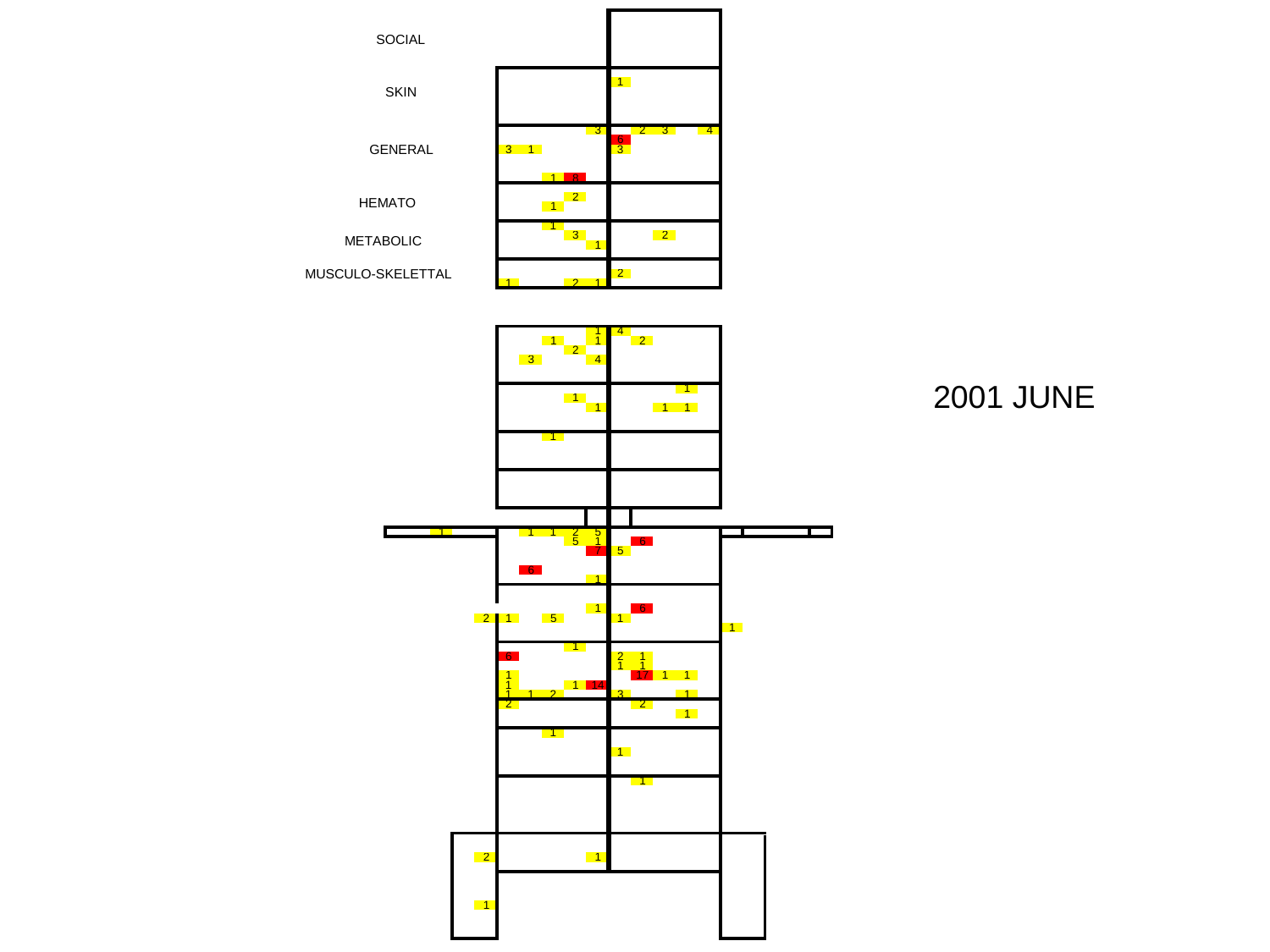

2001 JUNE

## Slide 50
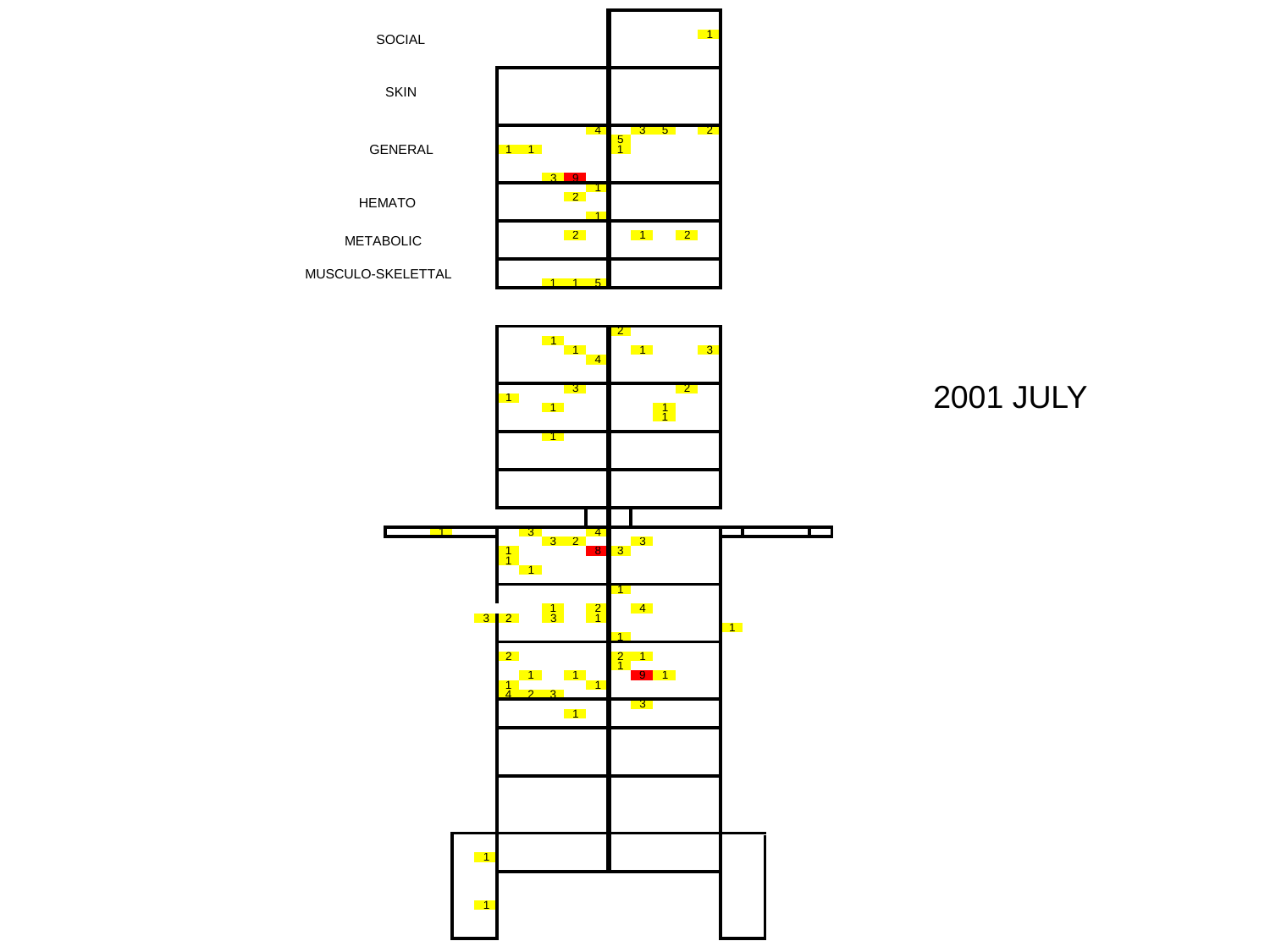

2001 JULY

## Slide 51
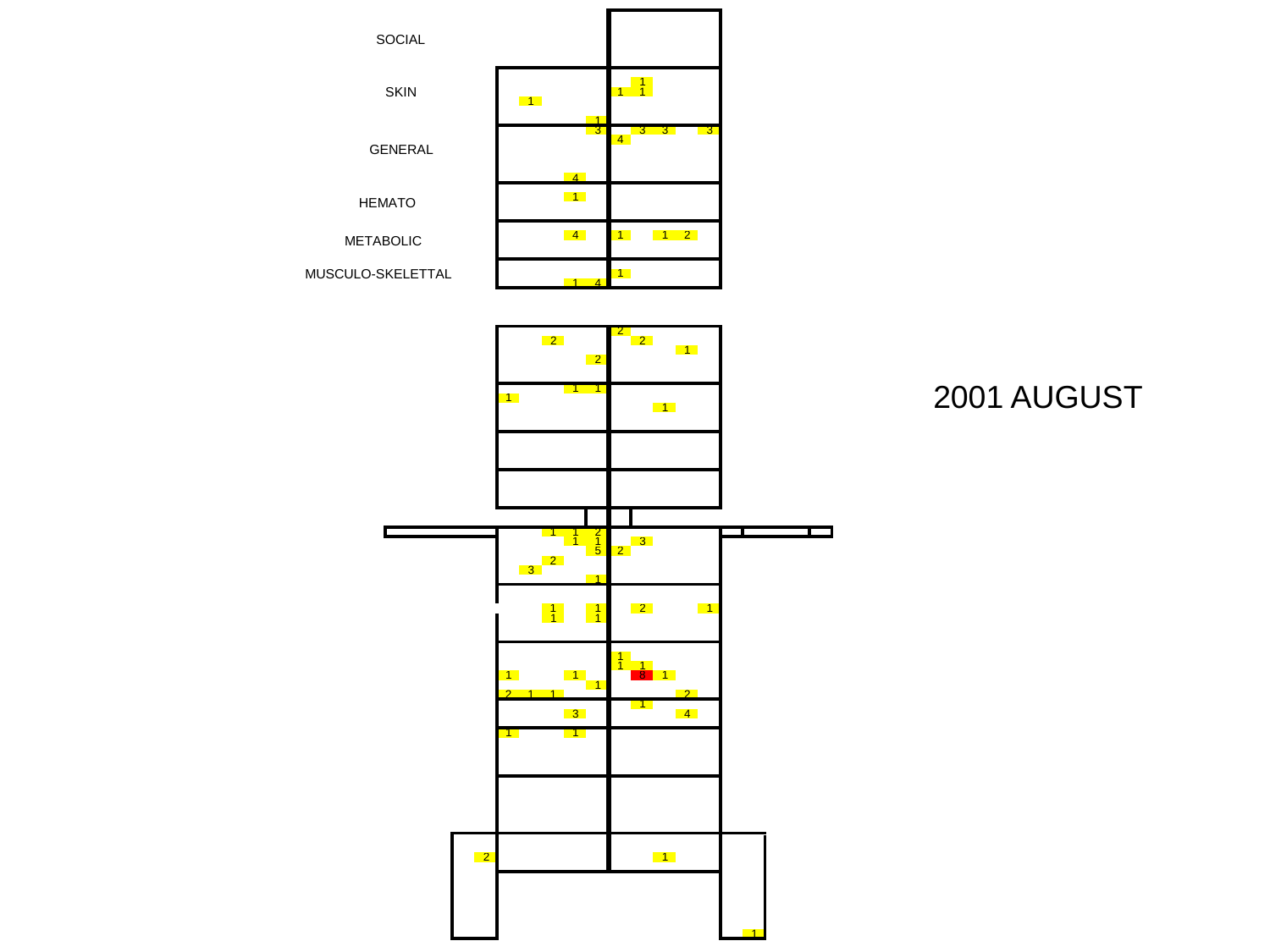

2001 AUGUST

## Slide 52
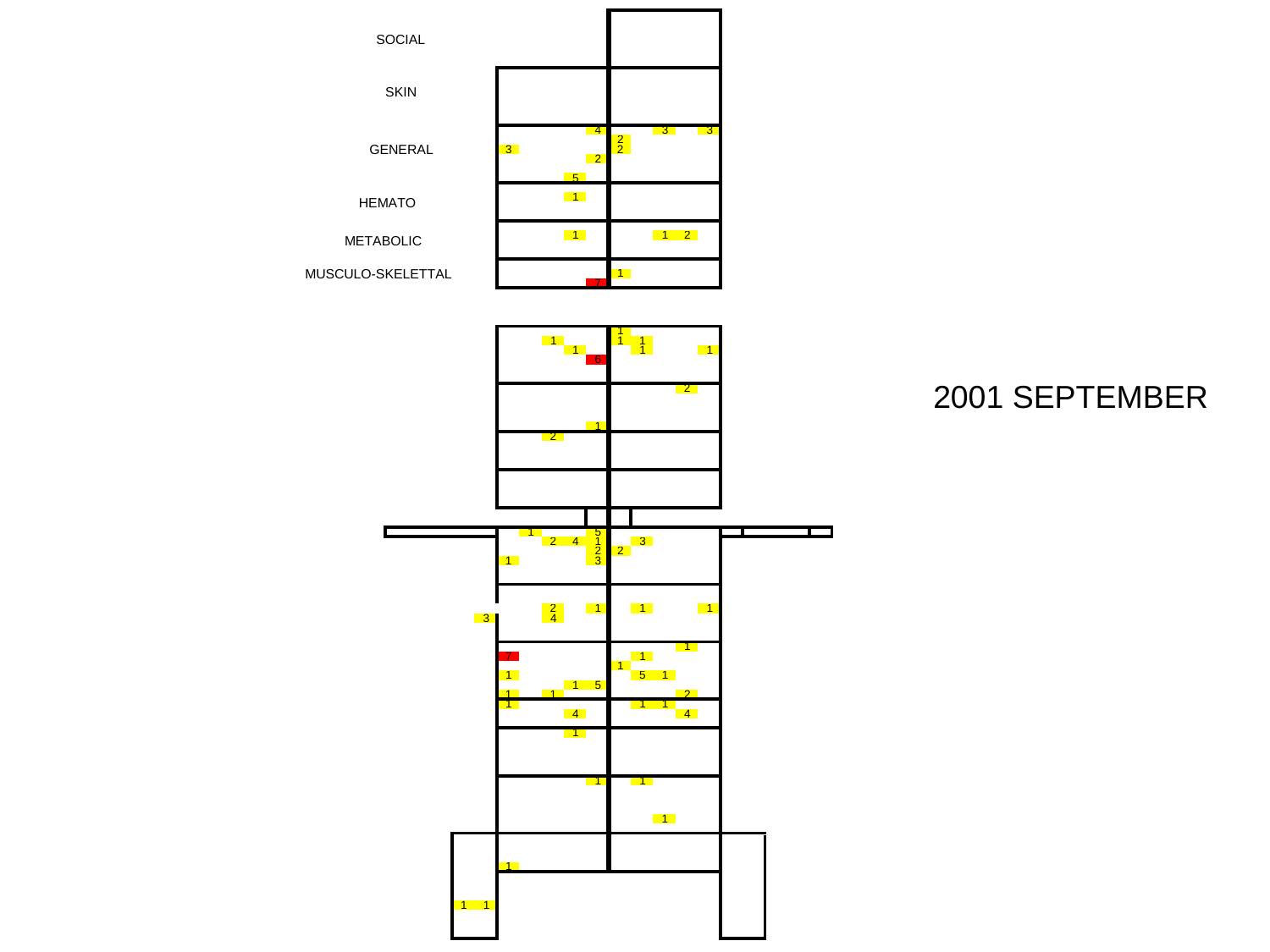

2001 SEPTEMBER

## Slide 53
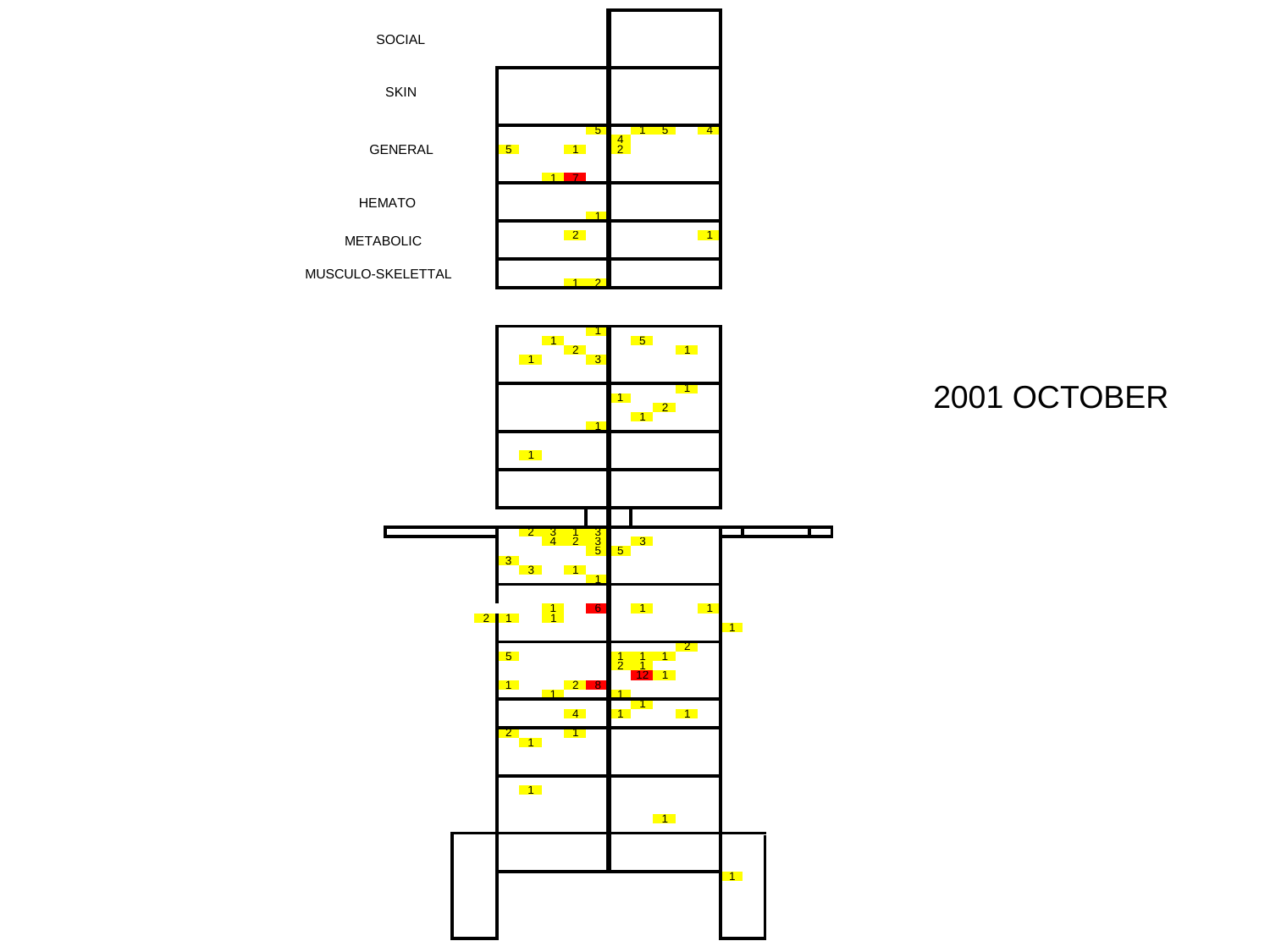

2001 OCTOBER

## Slide 54
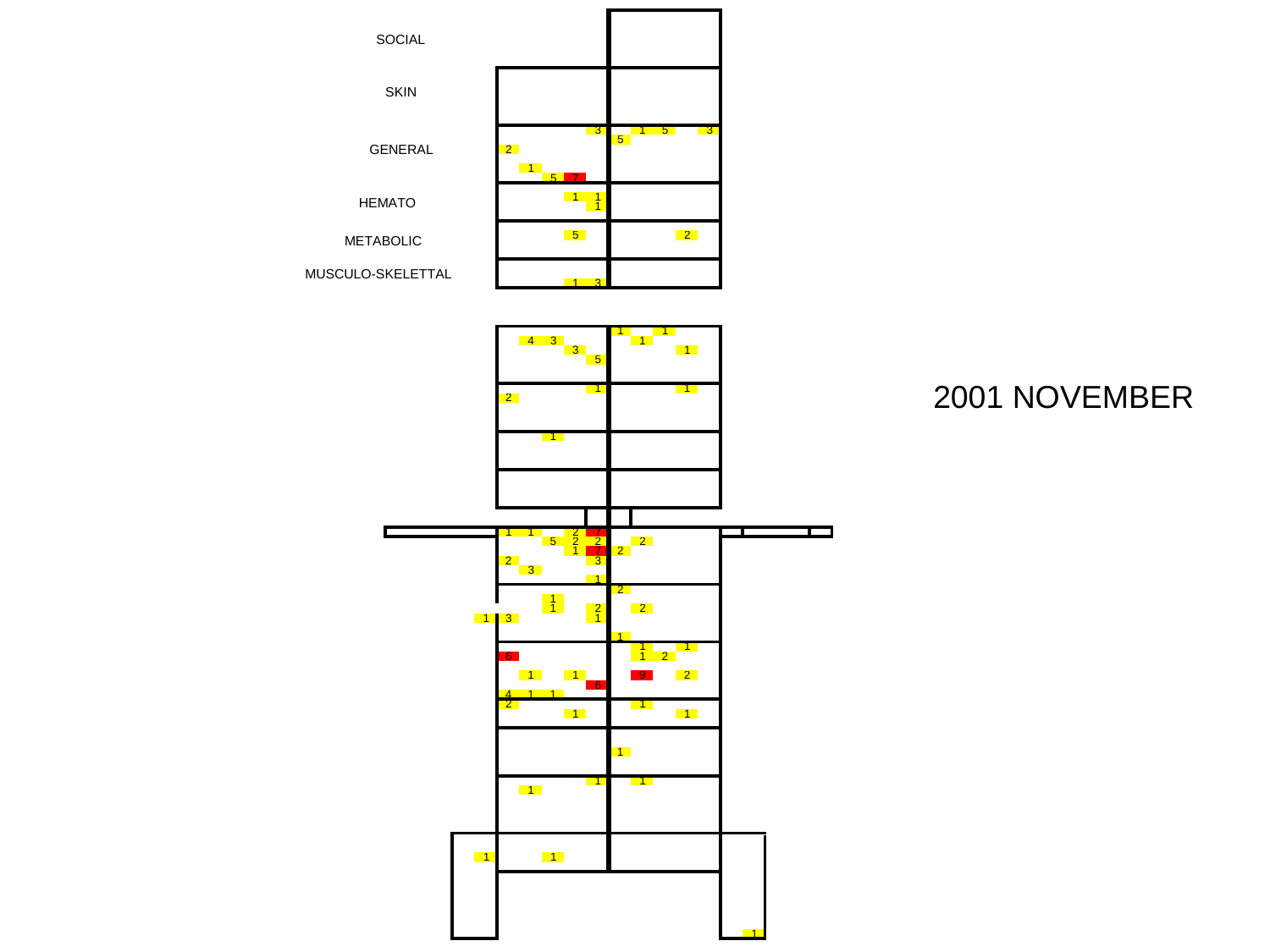

2001 NOVEMBER

## Slide 55
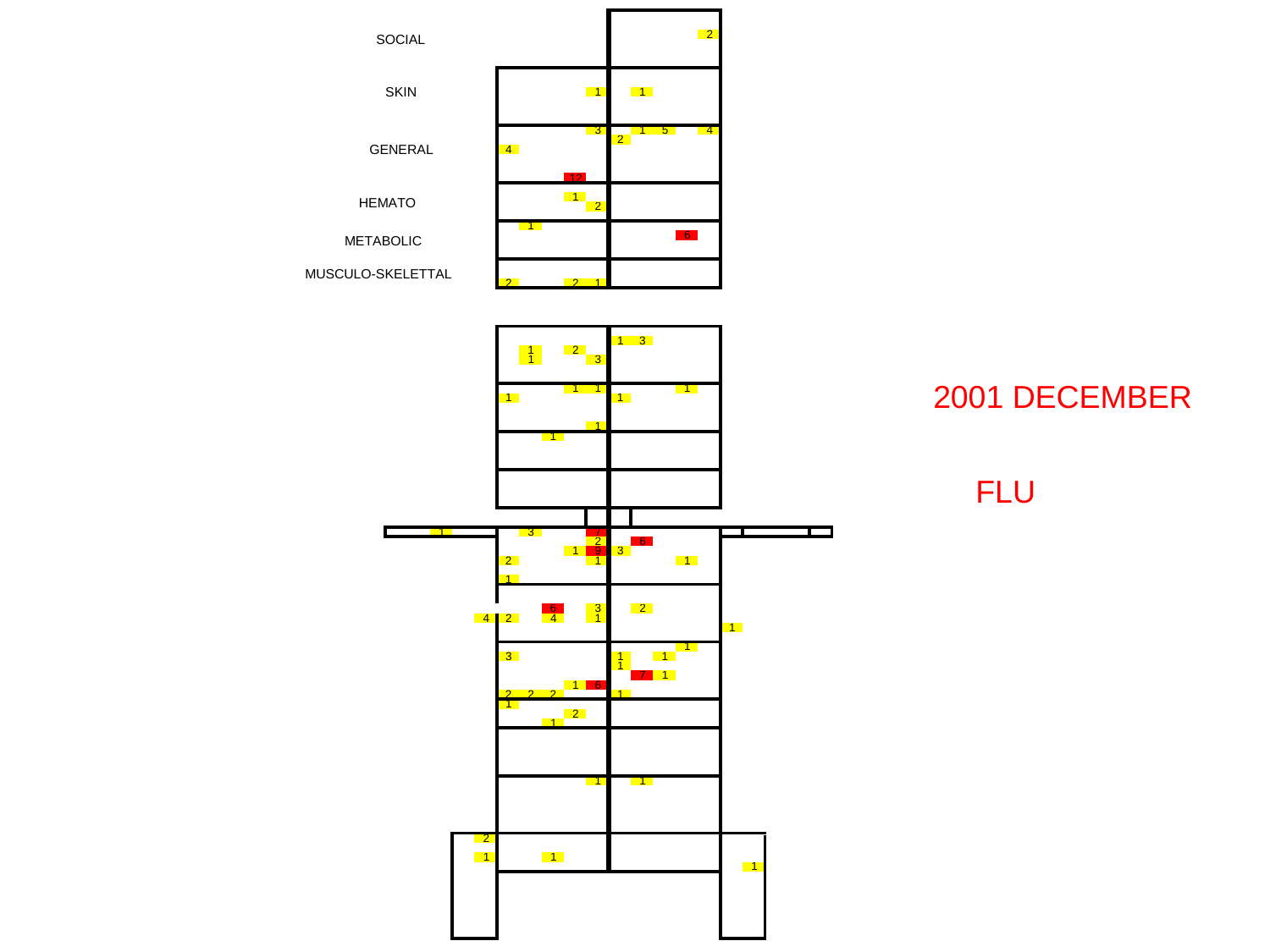

2001 DECEMBER
FLU

## Slide 56
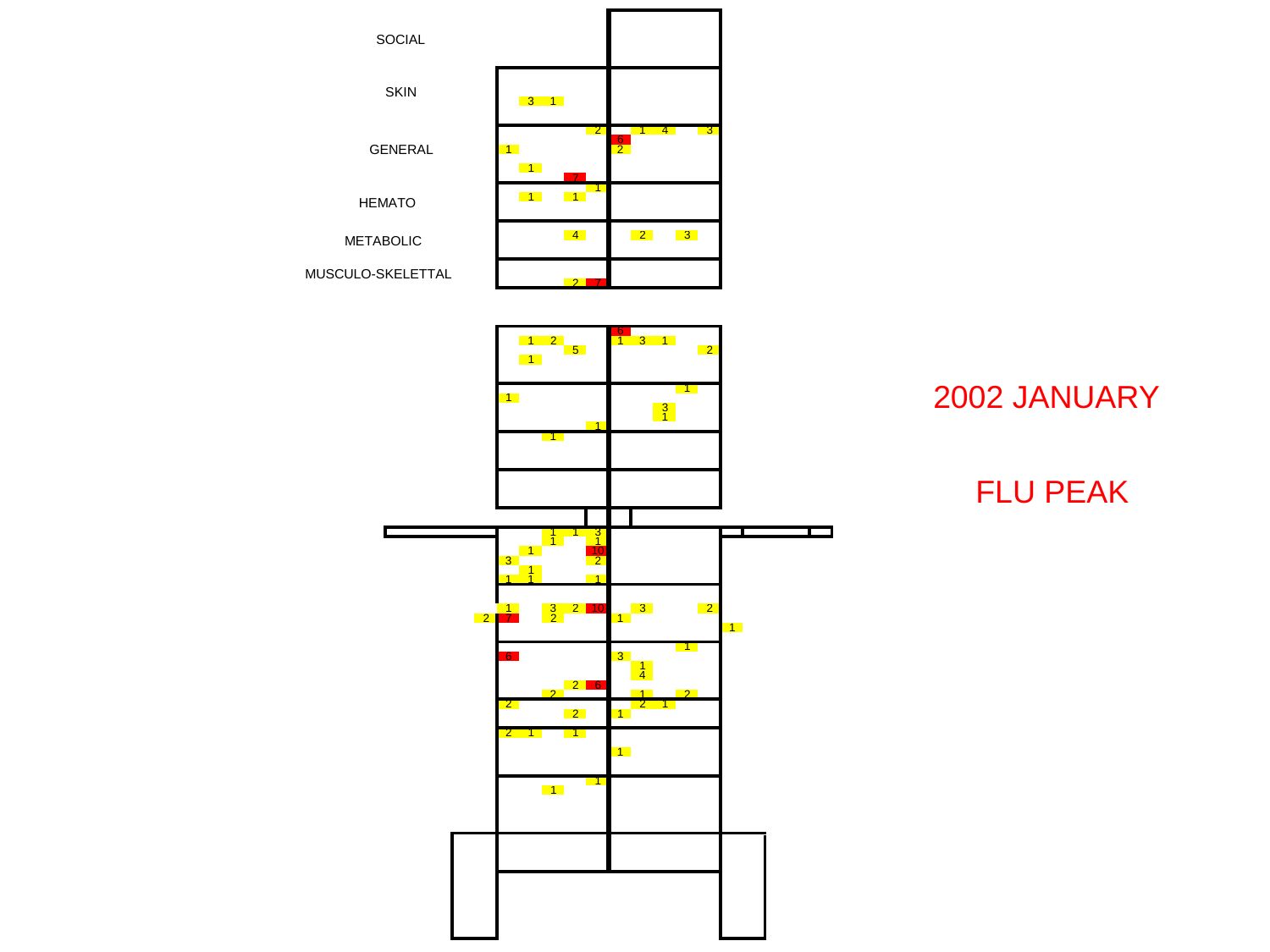

2002 JANUARY
FLU PEAK

## Slide 57
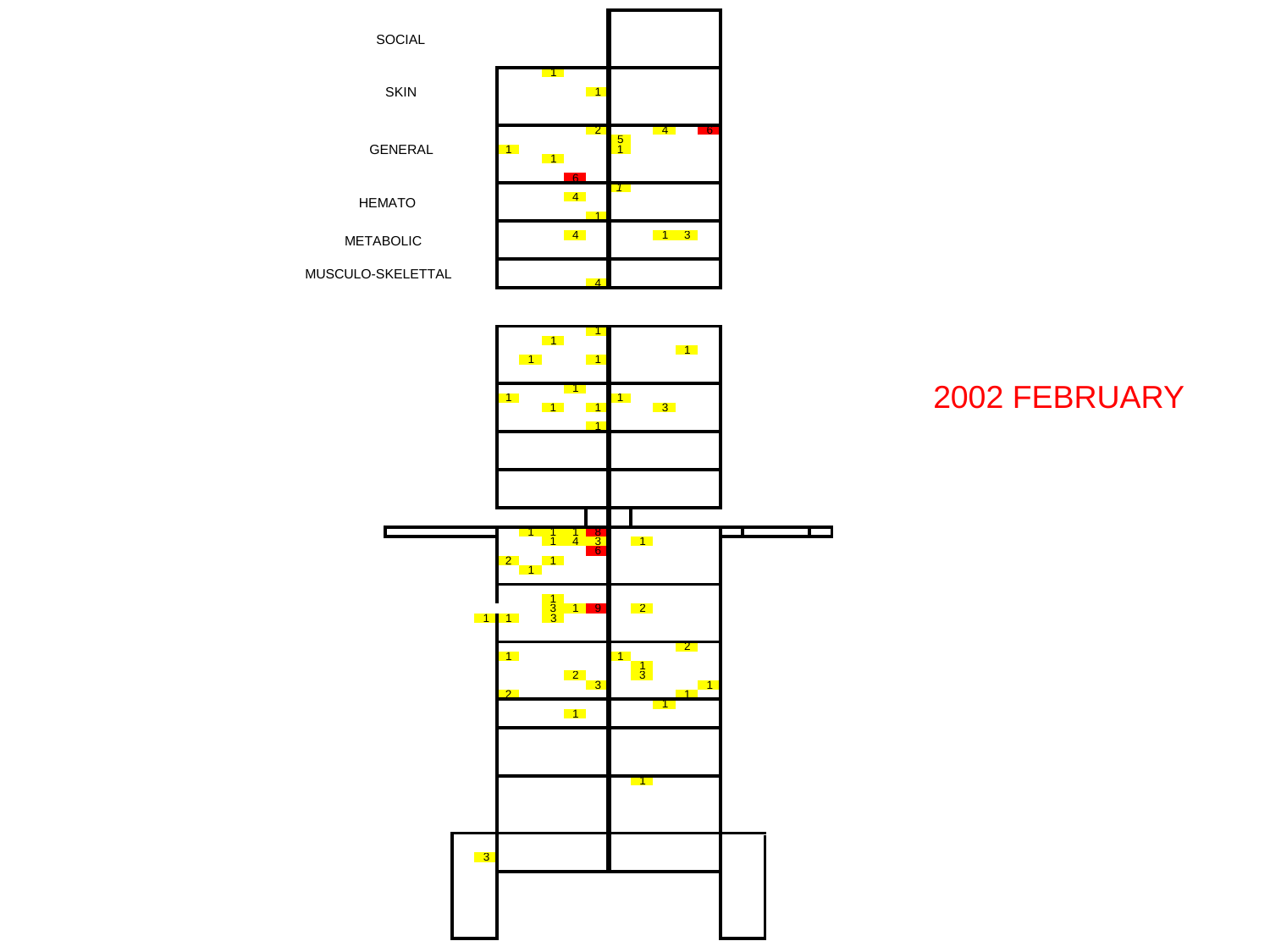

2002 FEBRUARY

## Slide 58
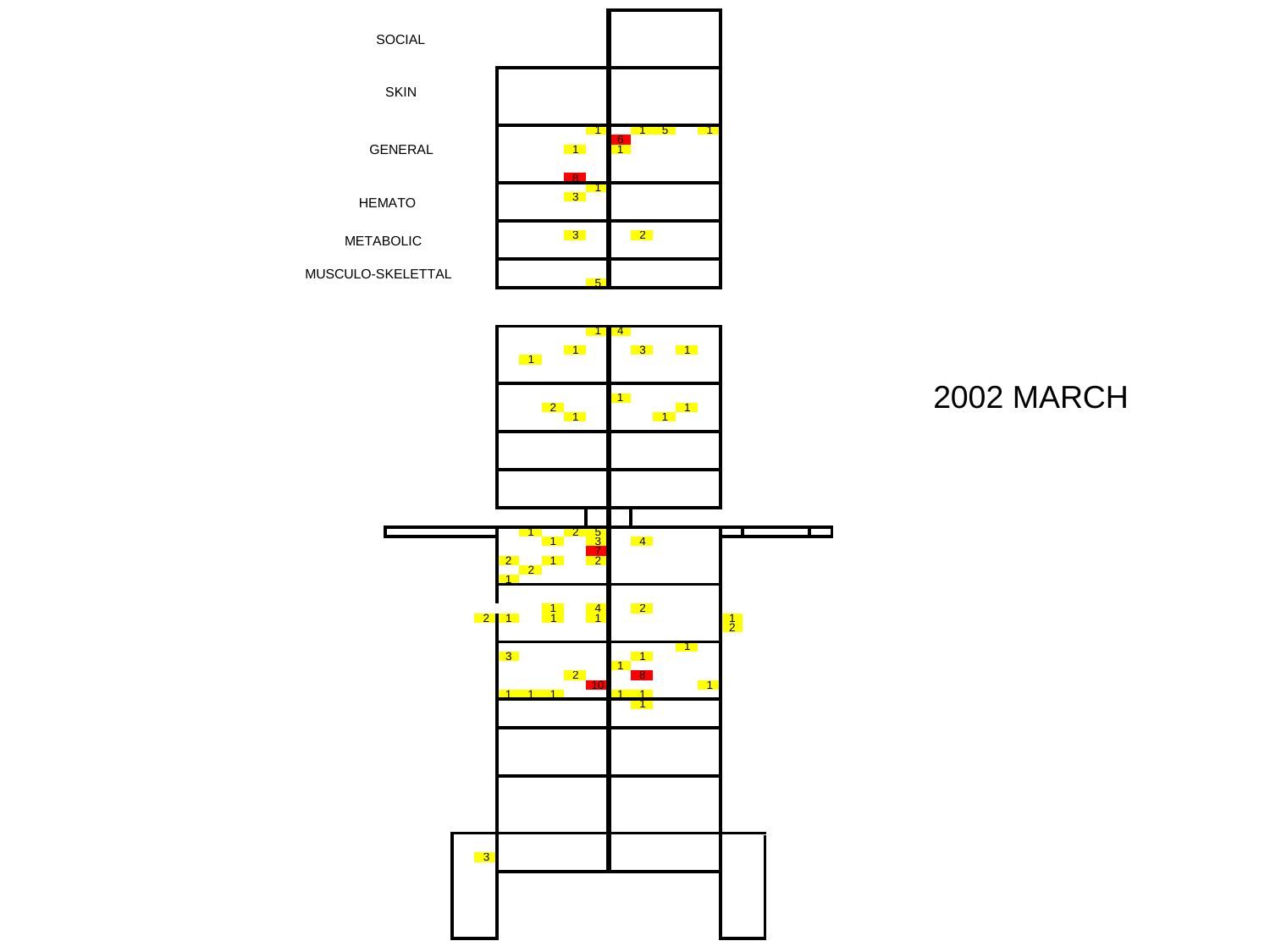

2002 MARCH

## Slide 59
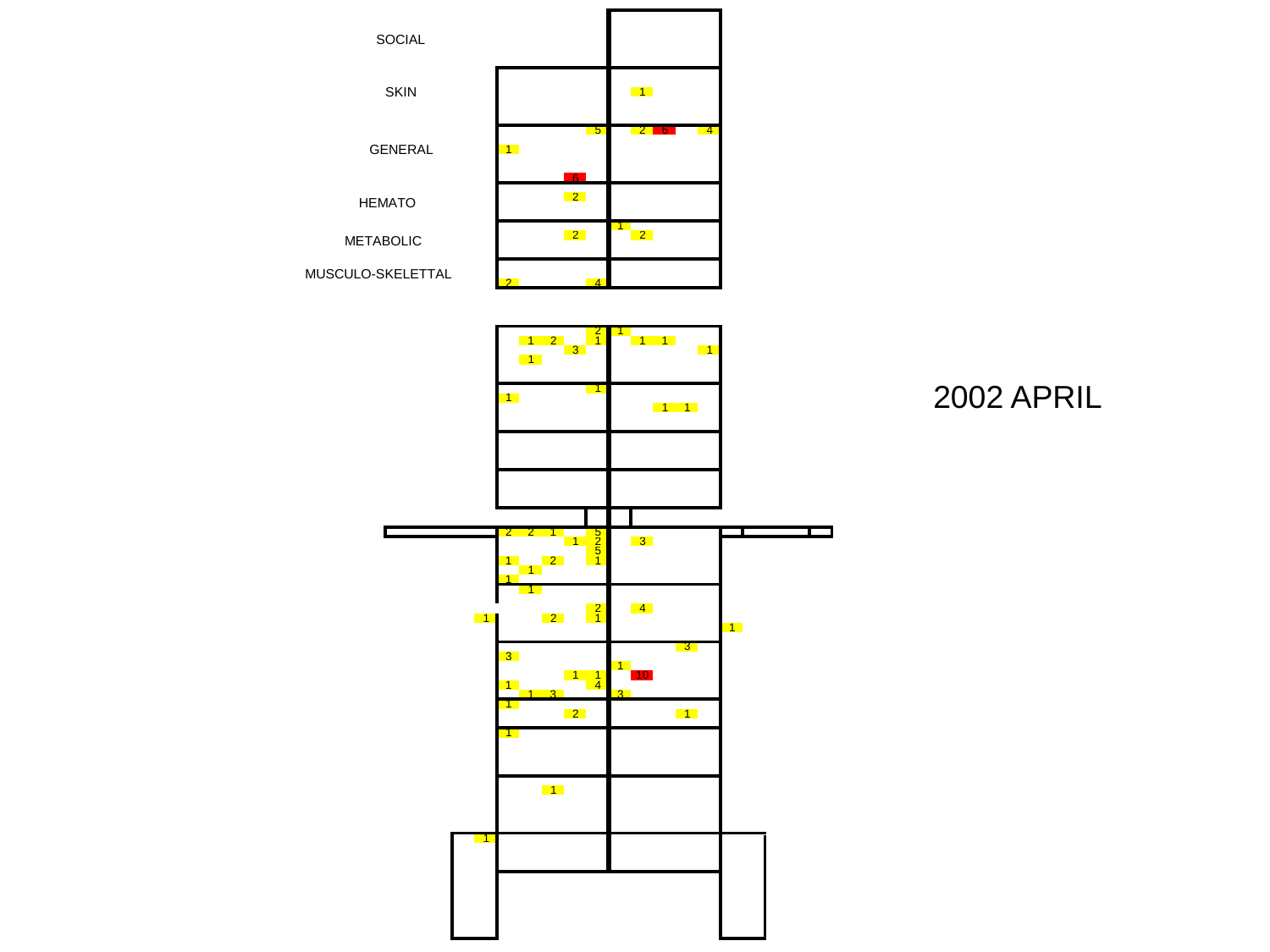

2002 APRIL

## Slide 60
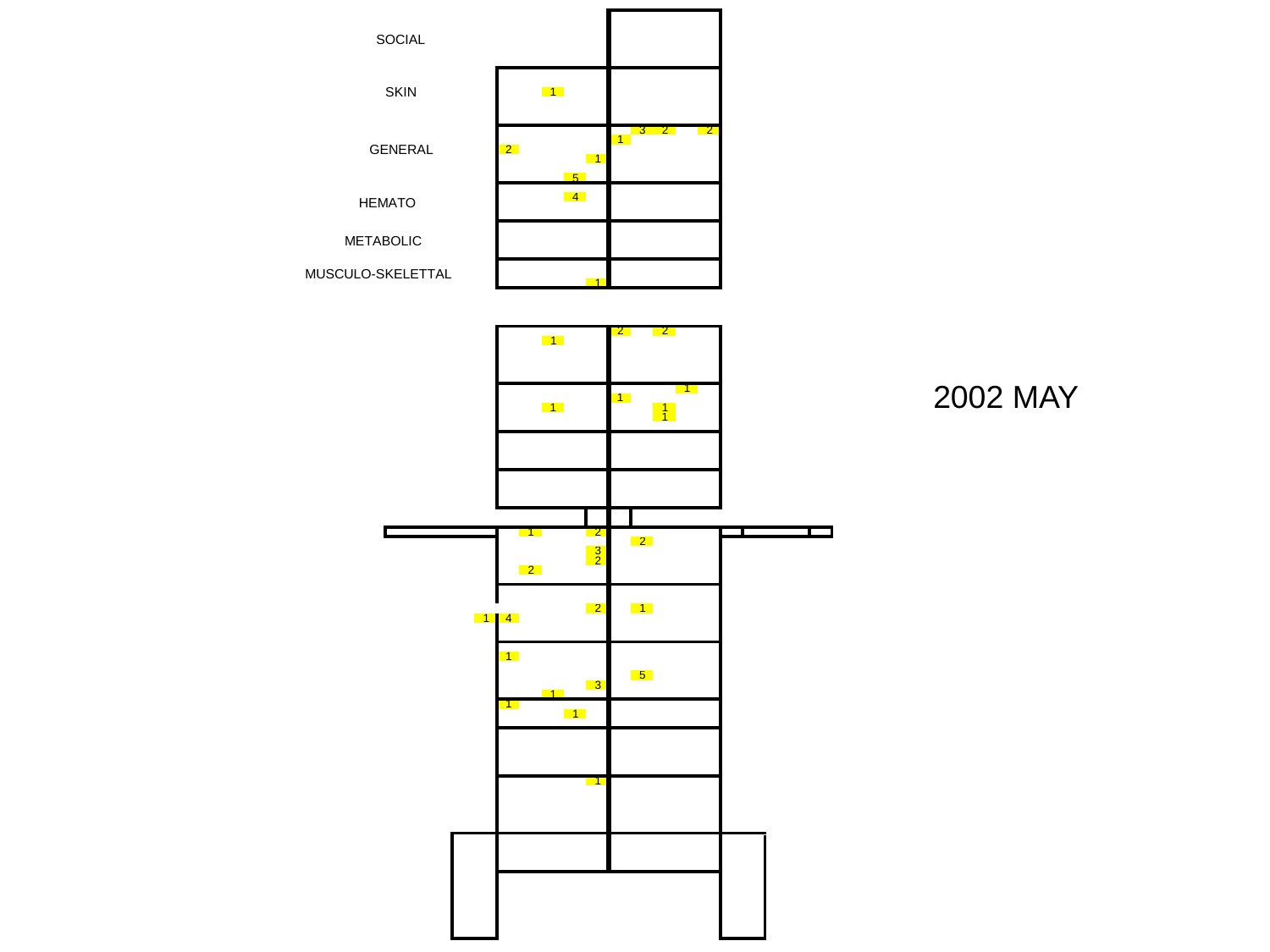

2002 MAY

## Slide 61
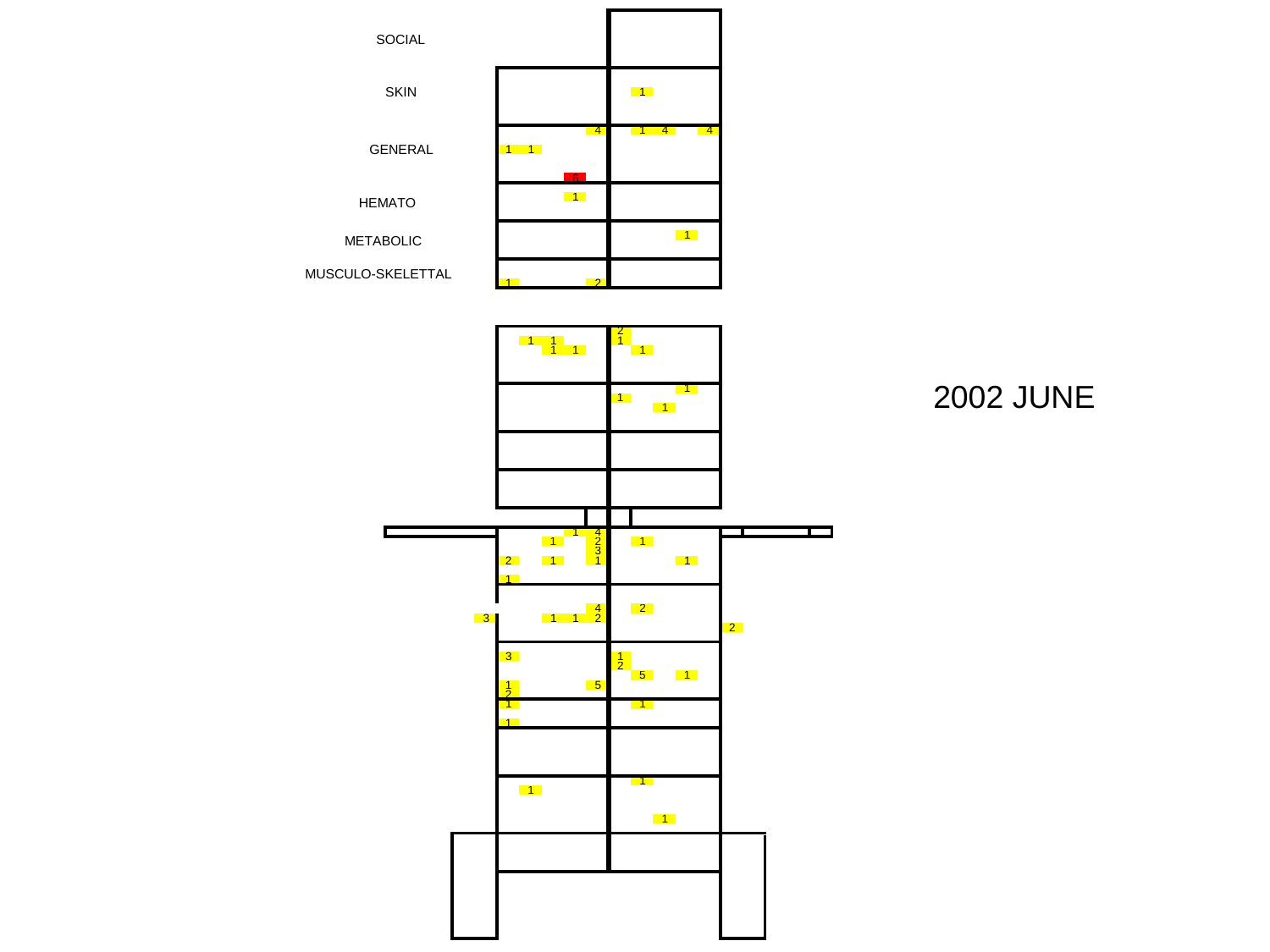

2002 JUNE

## Slide 62
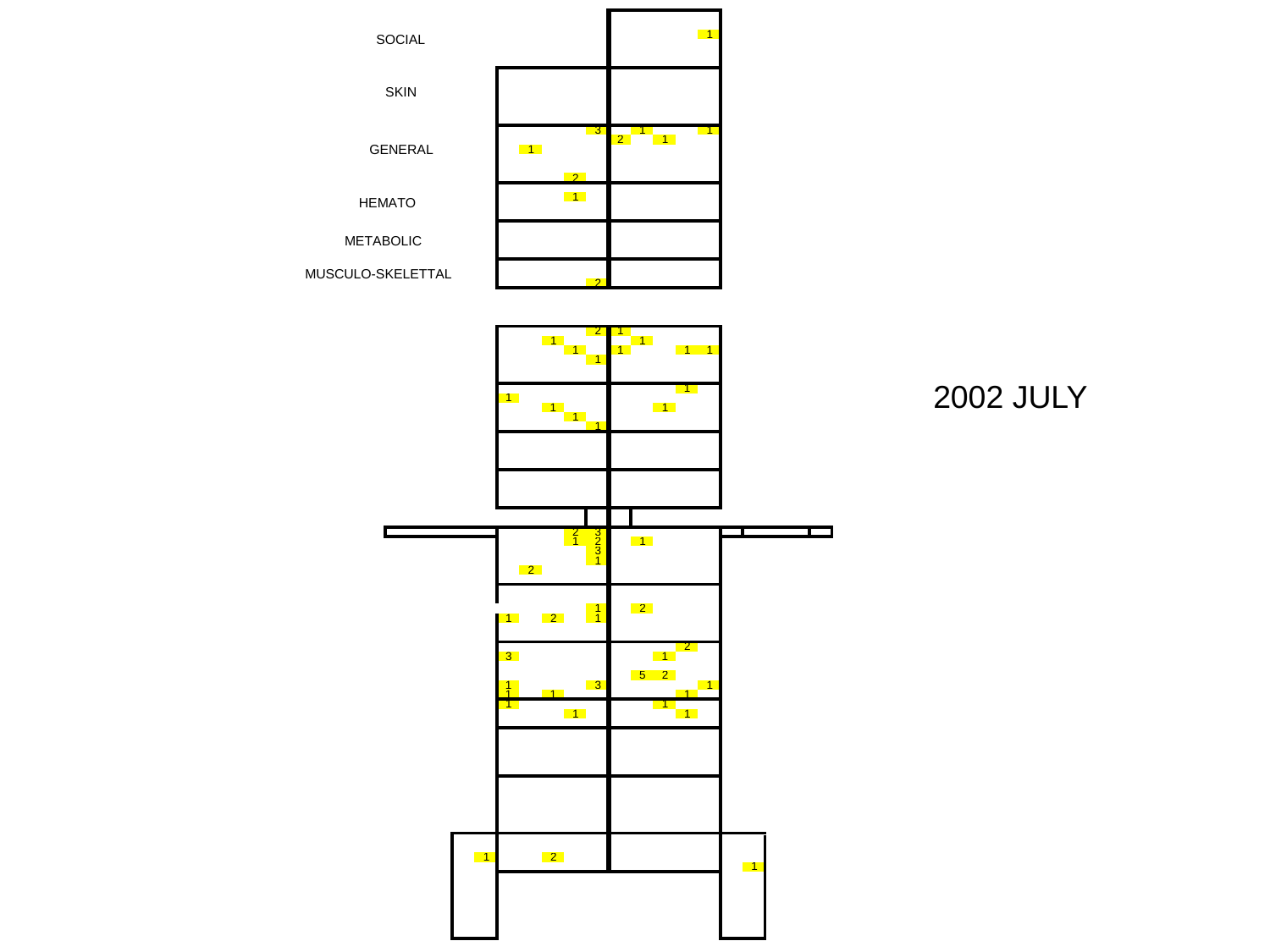

2002 JULY

## Slide 63
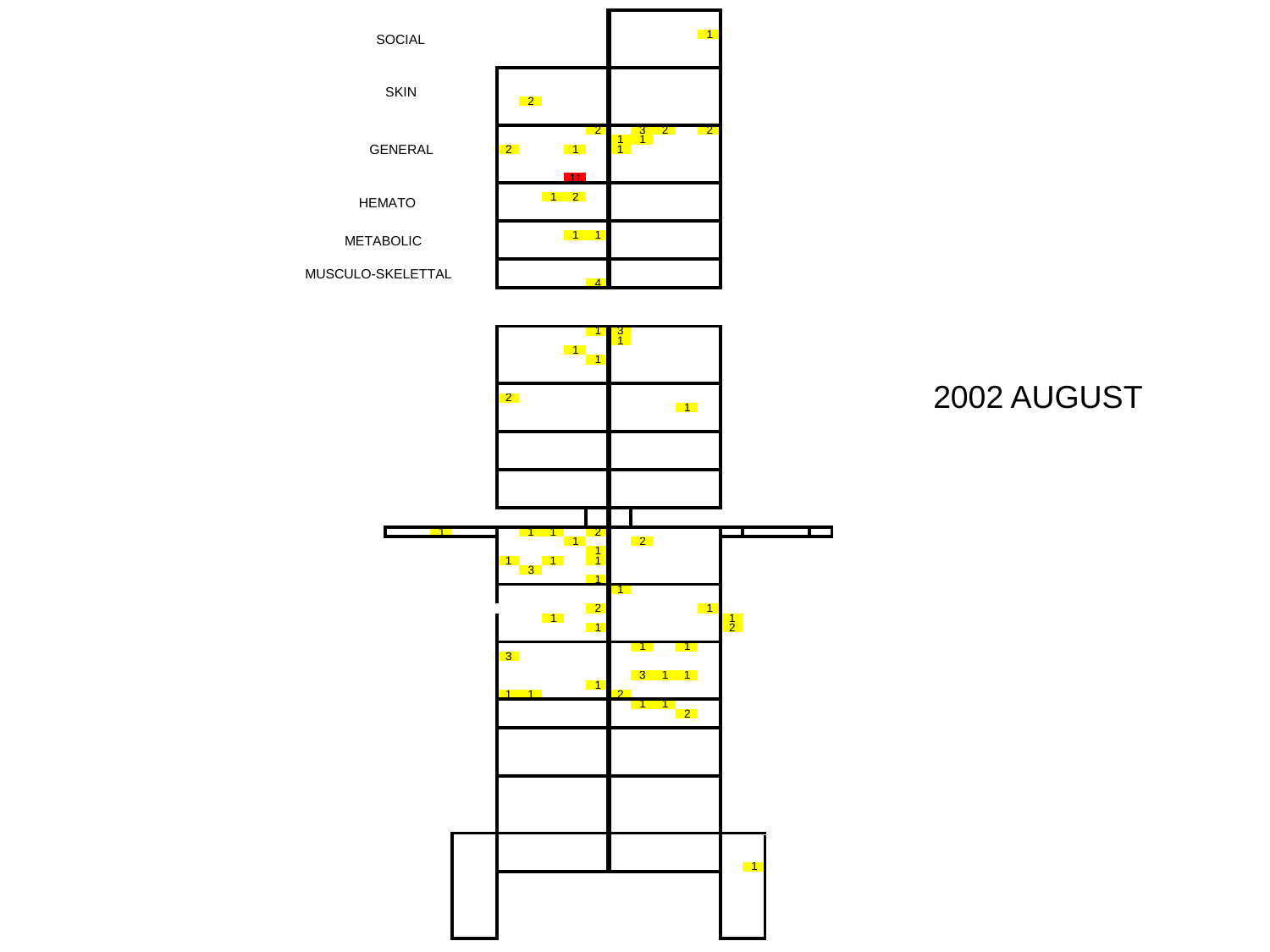

2002 AUGUST

## Slide 64
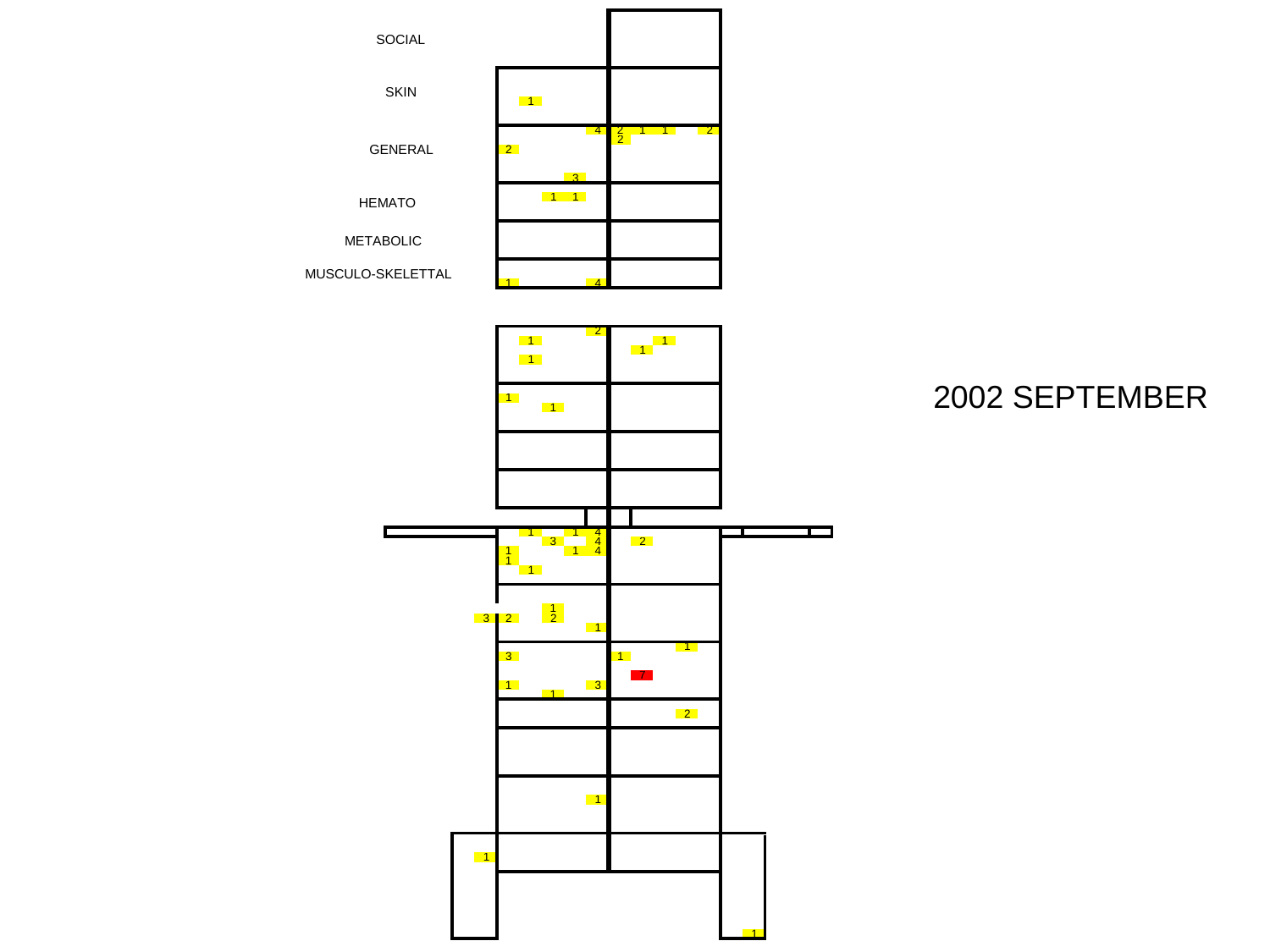

2002 SEPTEMBER

## Slide 65
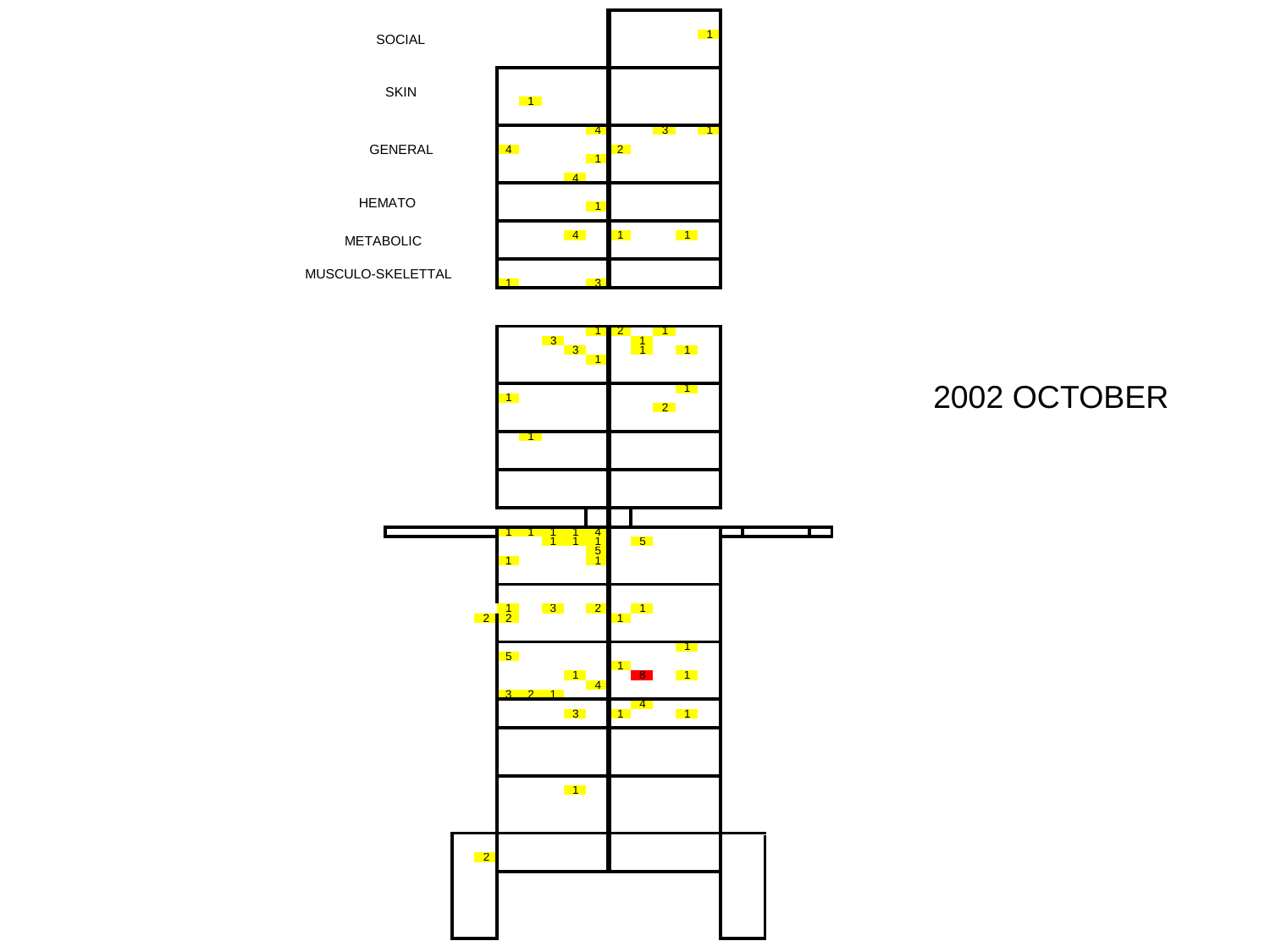

2002 OCTOBER

## Slide 66
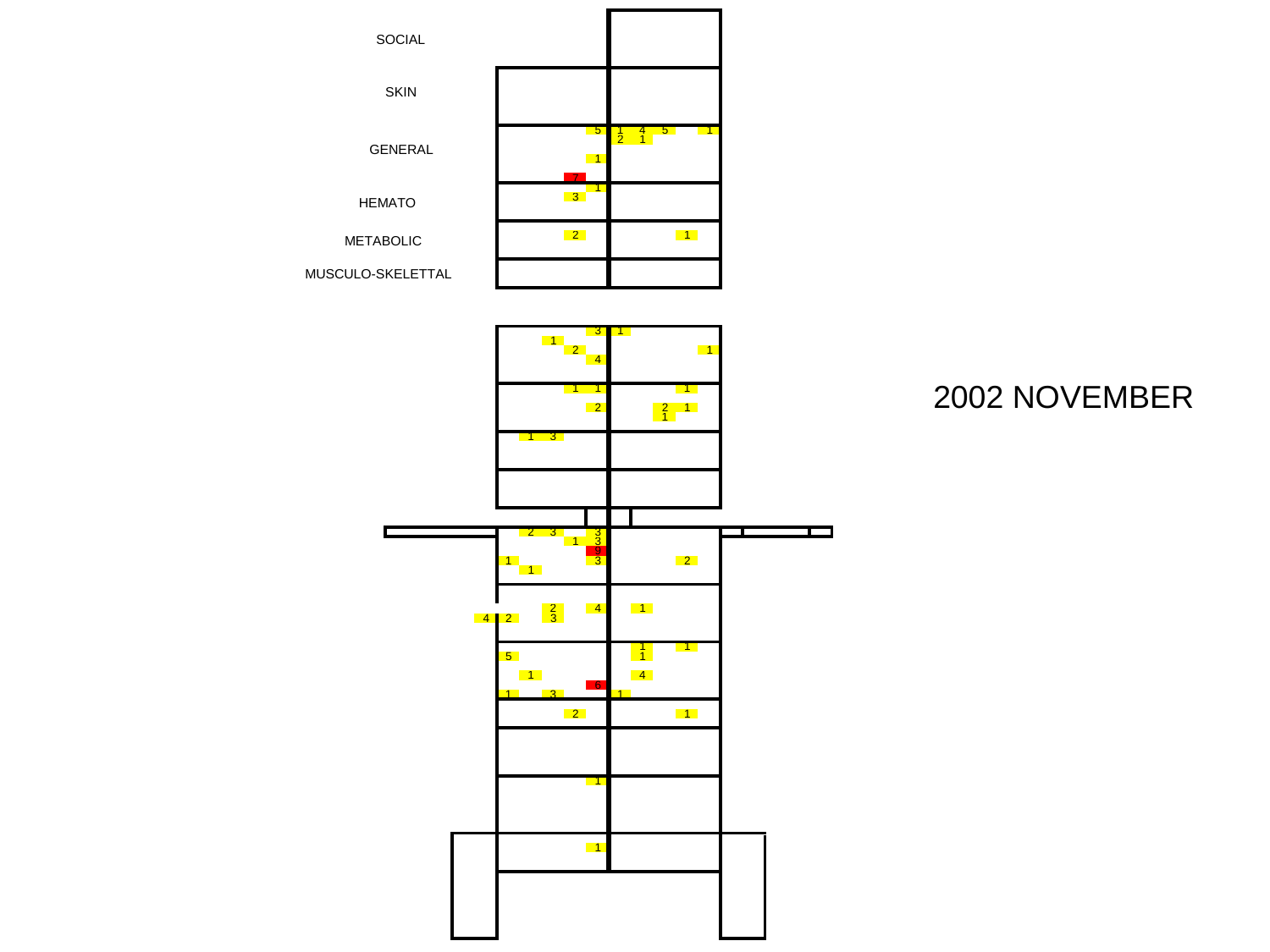

2002 NOVEMBER

## Slide 67
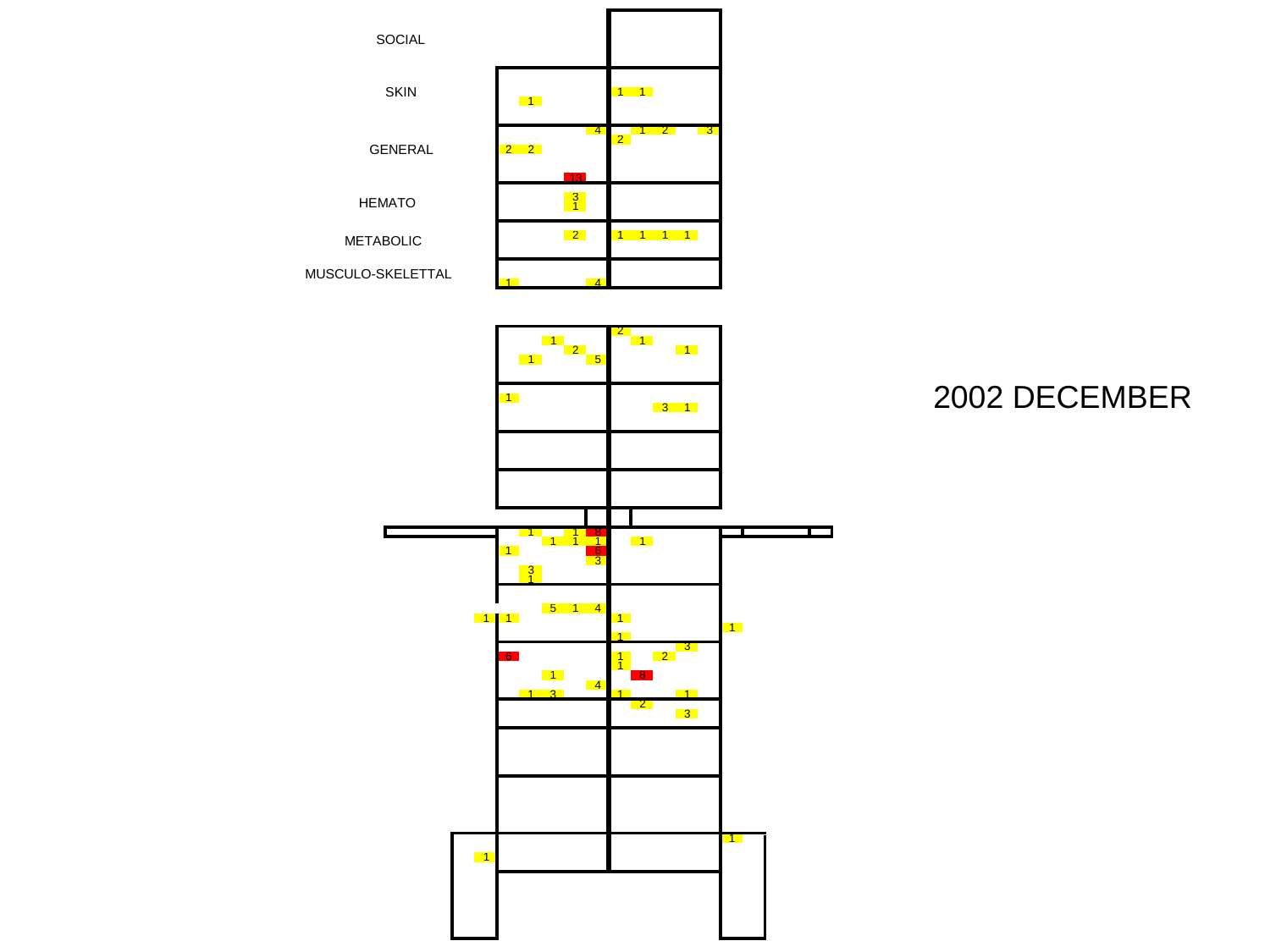

2002 DECEMBER

## Slide 68
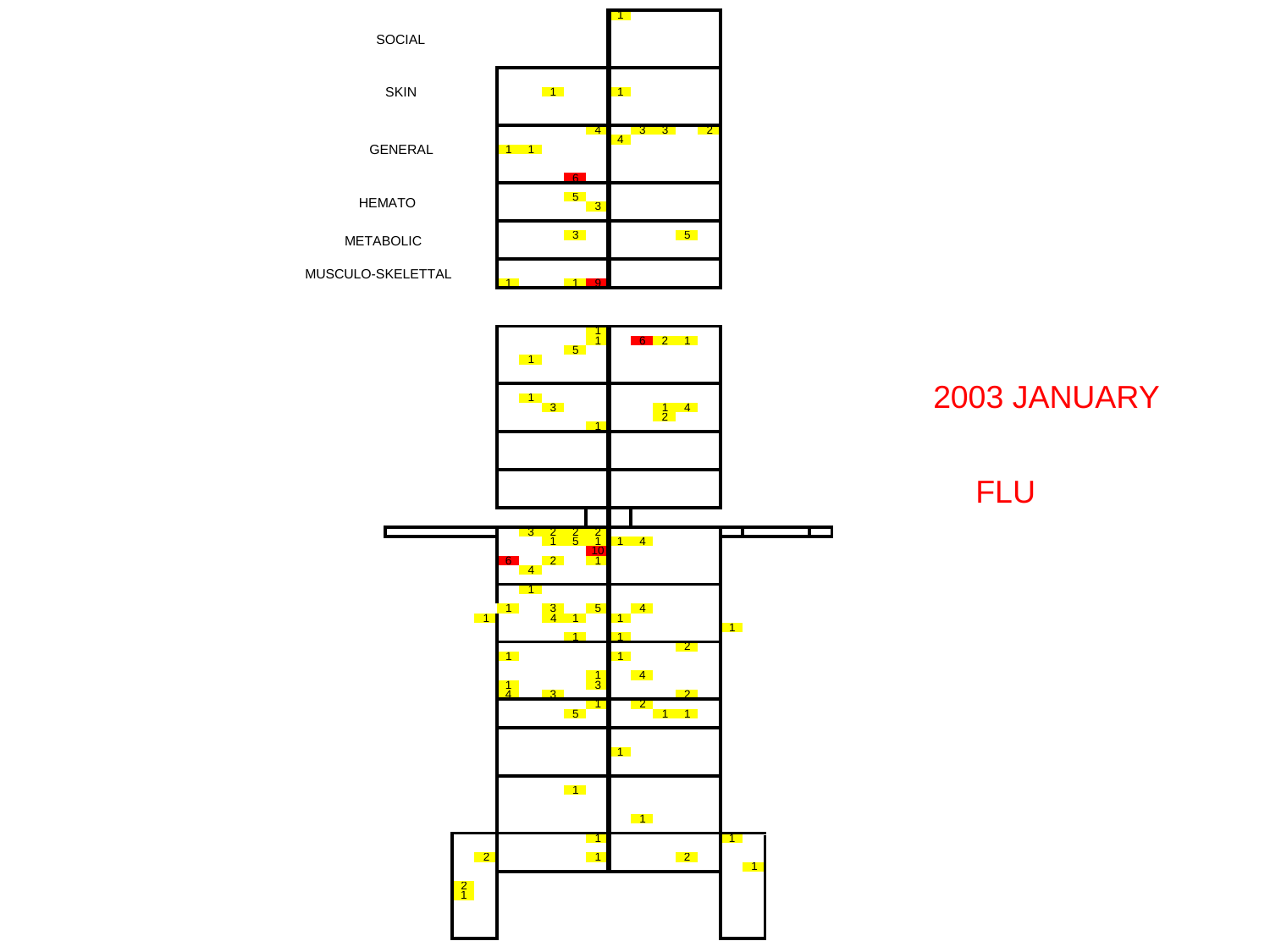

2003 JANUARY
FLU

## Slide 69
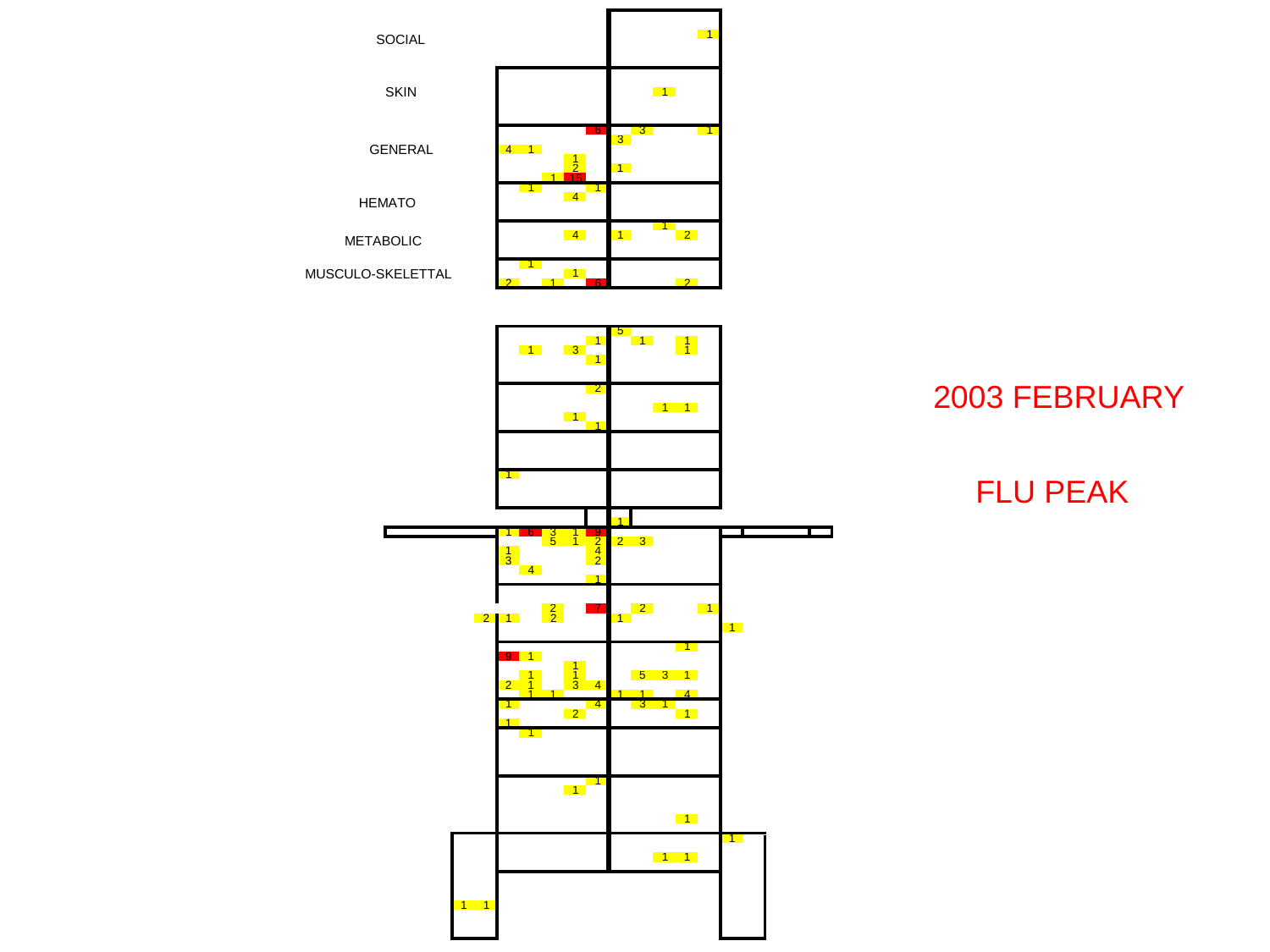

2003 FEBRUARY
FLU PEAK

## Slide 70
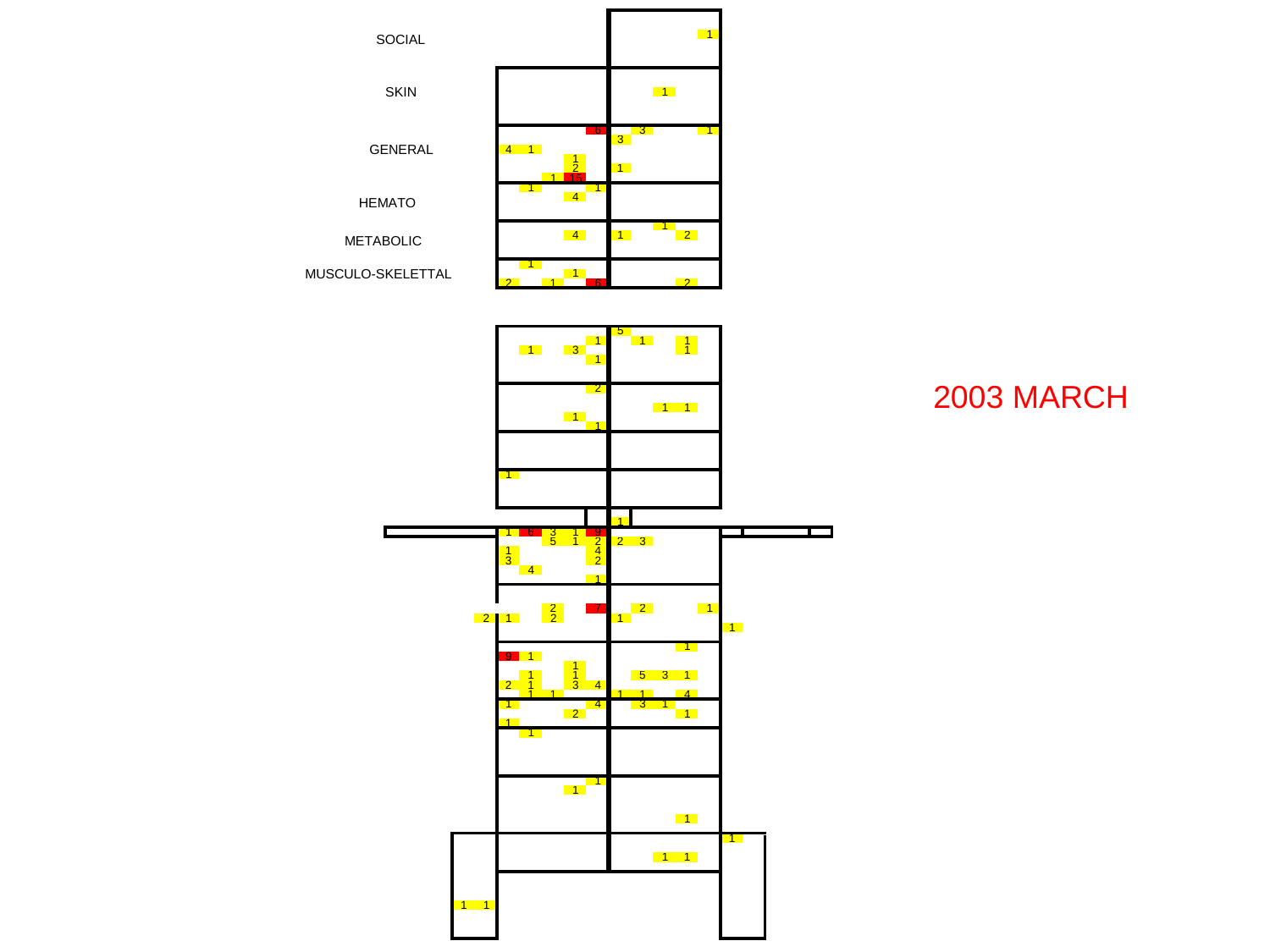

2003 MARCH

## Slide 71
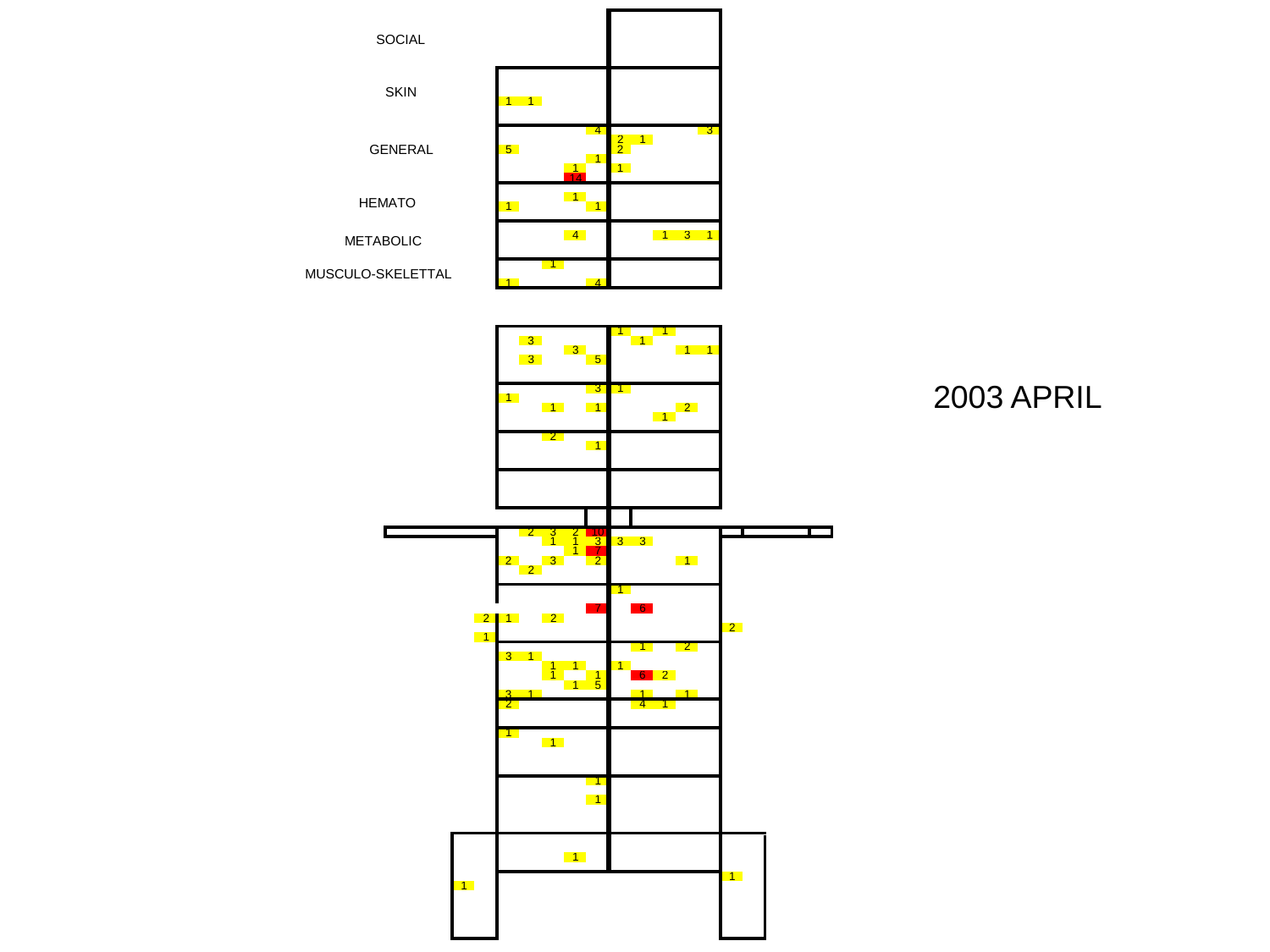

2003 APRIL

## Slide 72
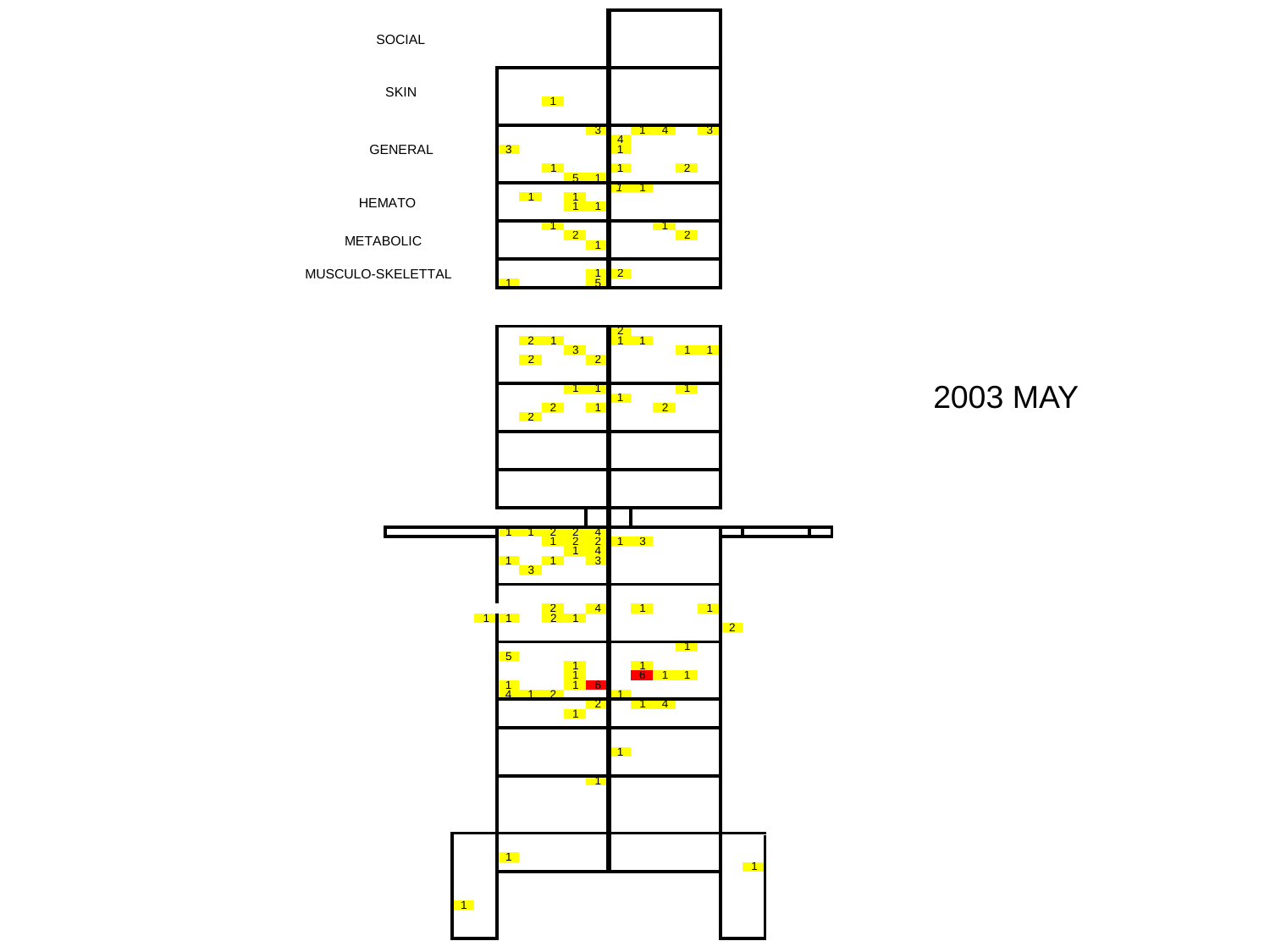

2003 MAY

## Slide 73
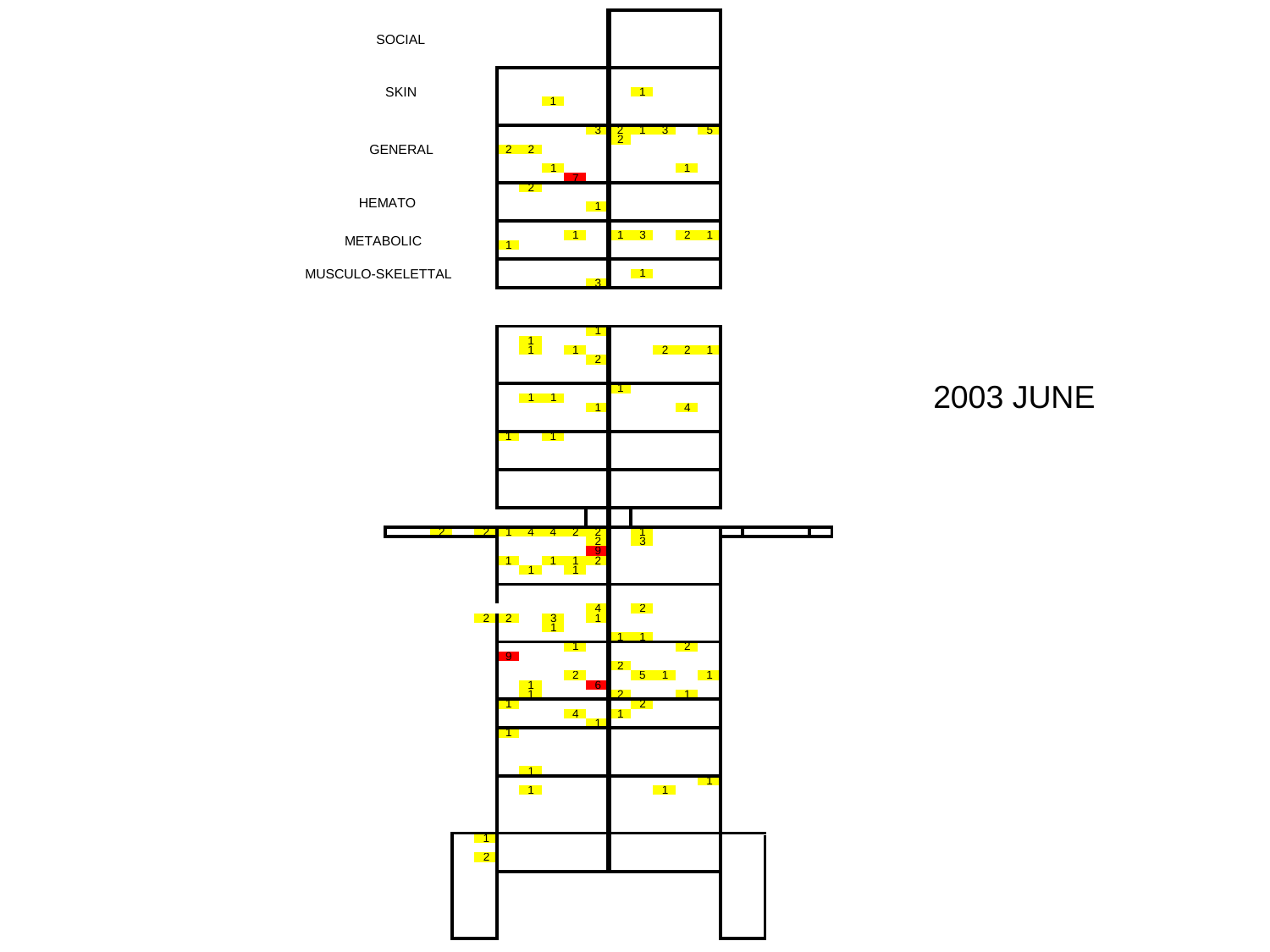

2003 JUNE

## Slide 74
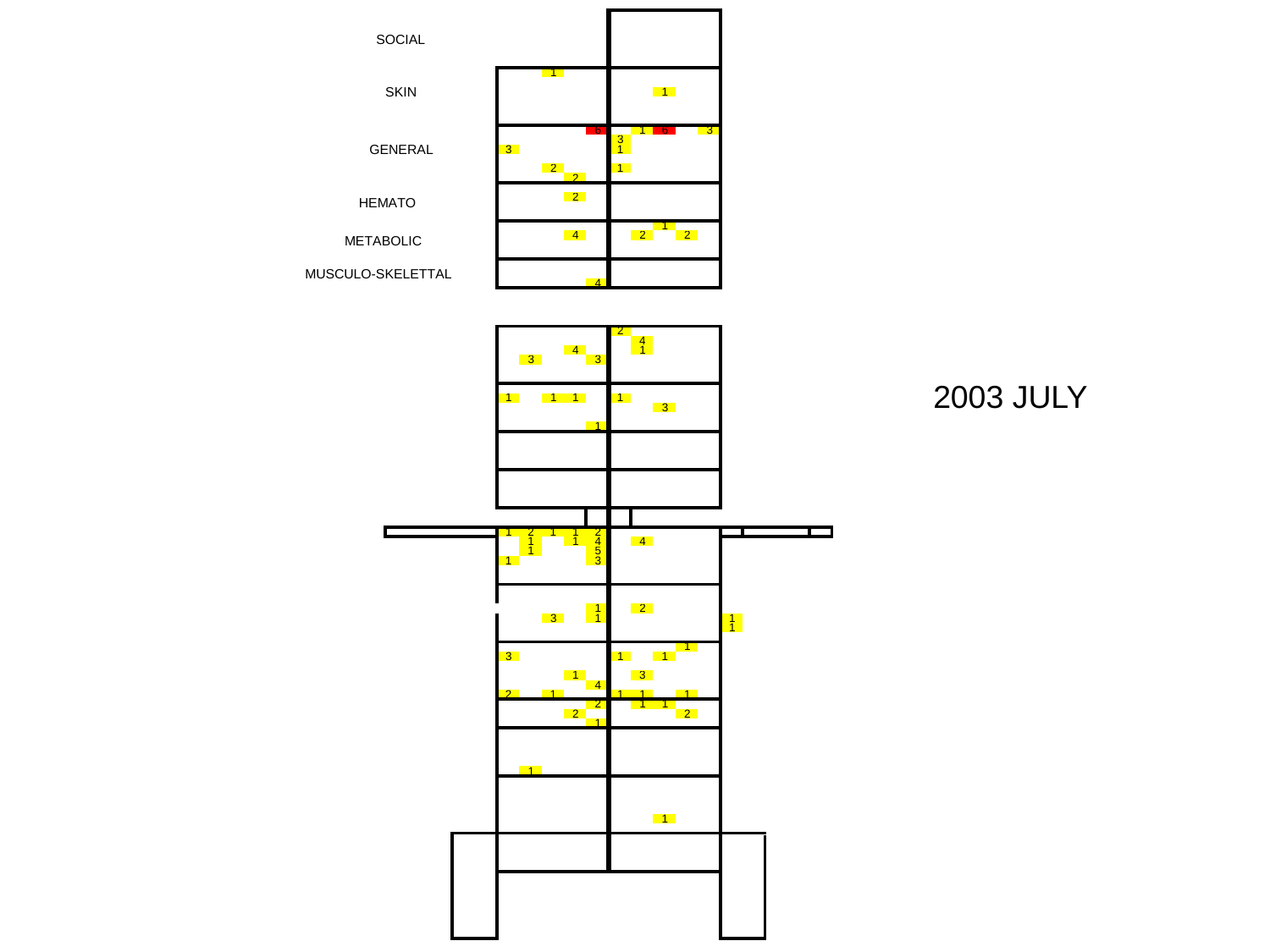

2003 JULY

## Slide 75
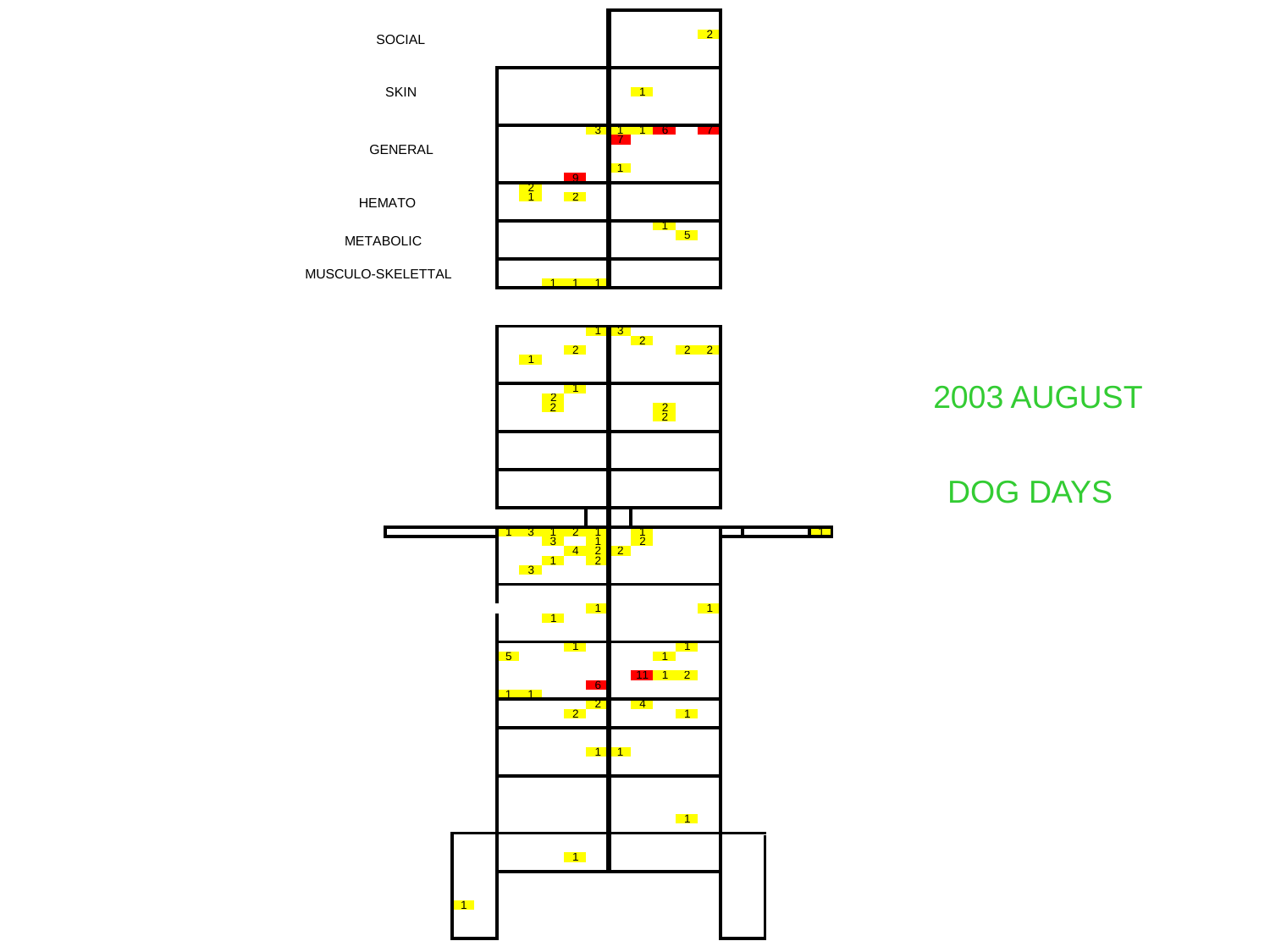

2003 AUGUST
DOG DAYS

## Slide 76
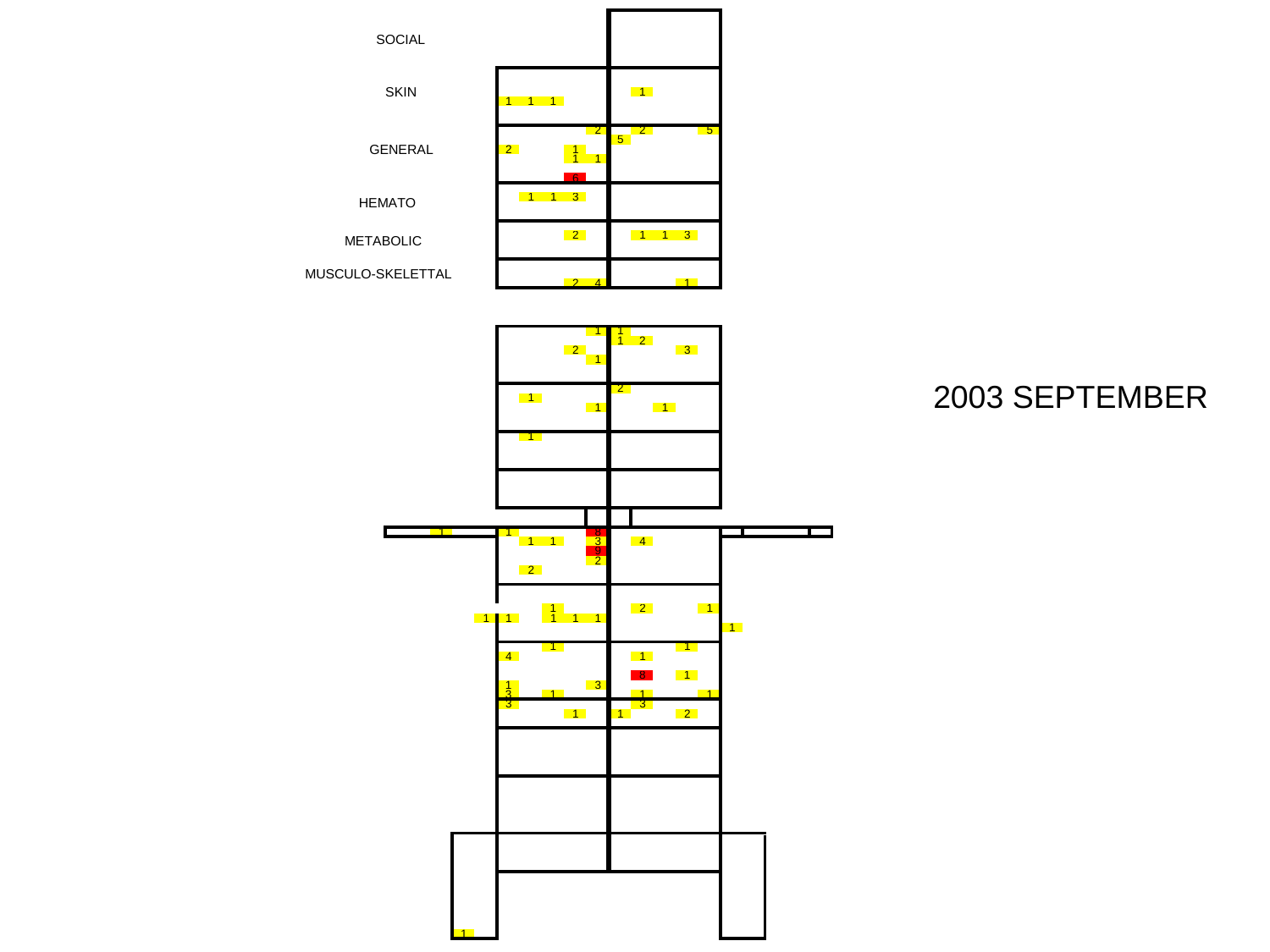

2003 SEPTEMBER

## Slide 77
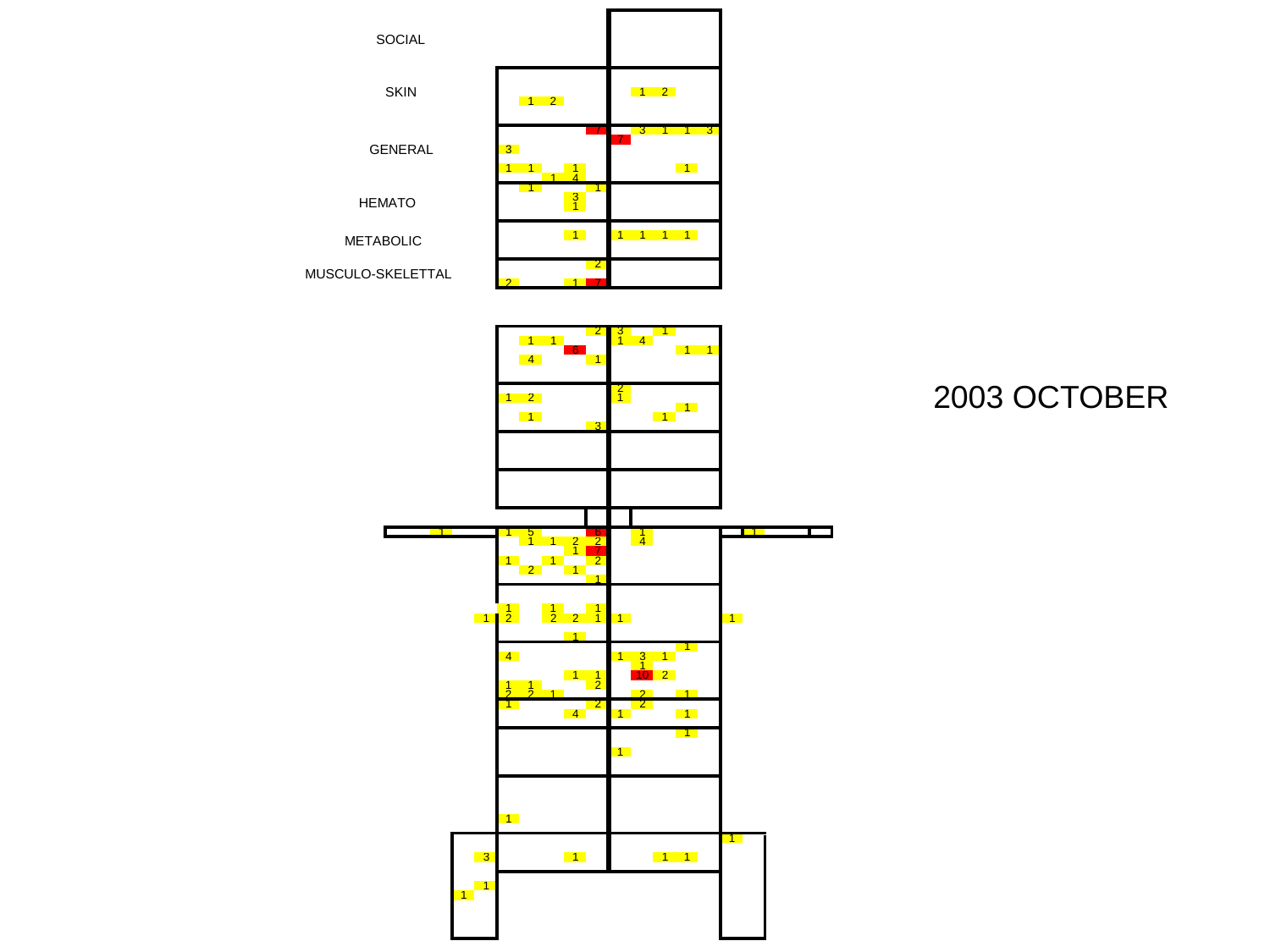

2003 OCTOBER

## Slide 78
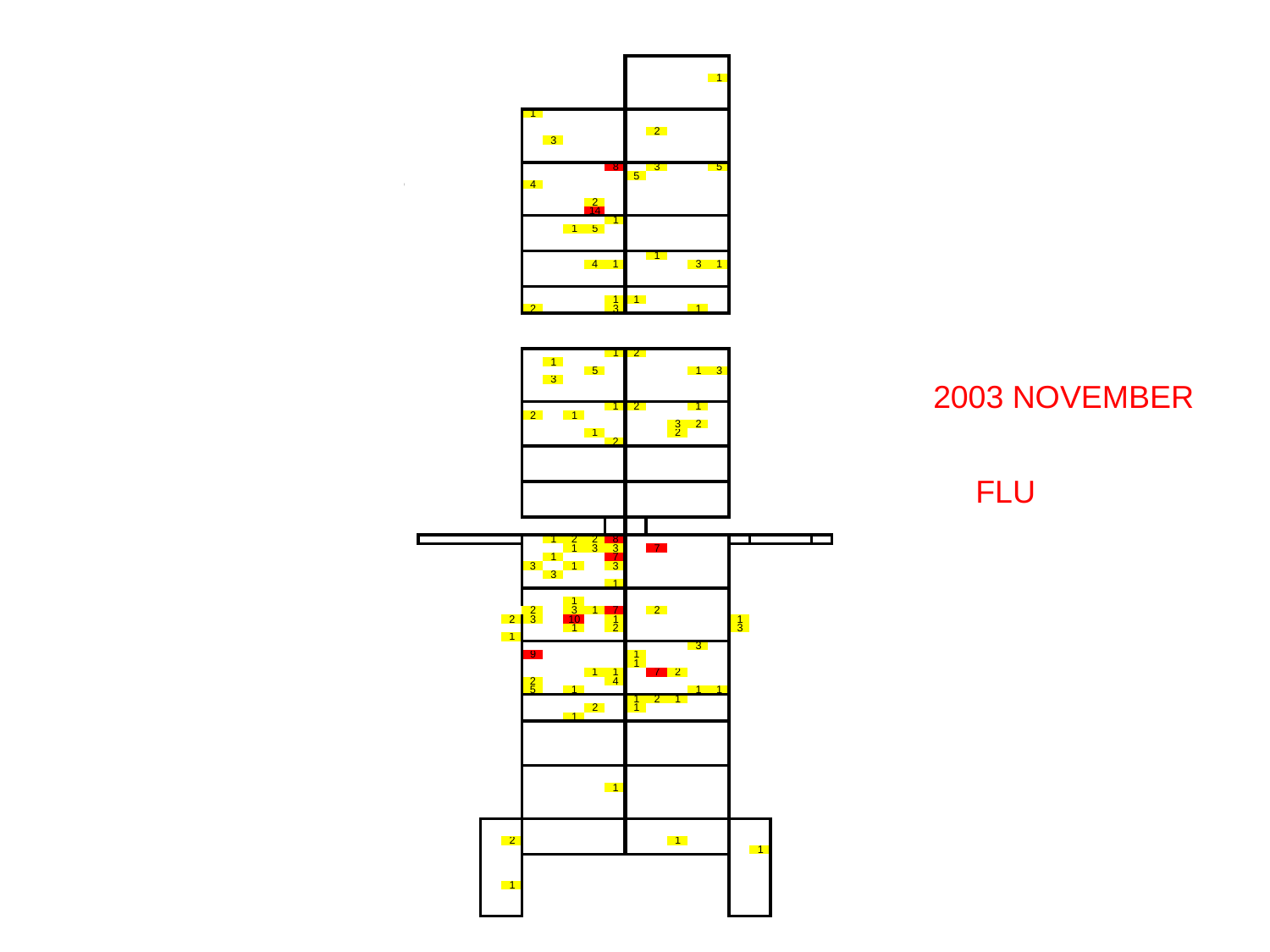

2003 NOVEMBER
FLU

## Slide 79
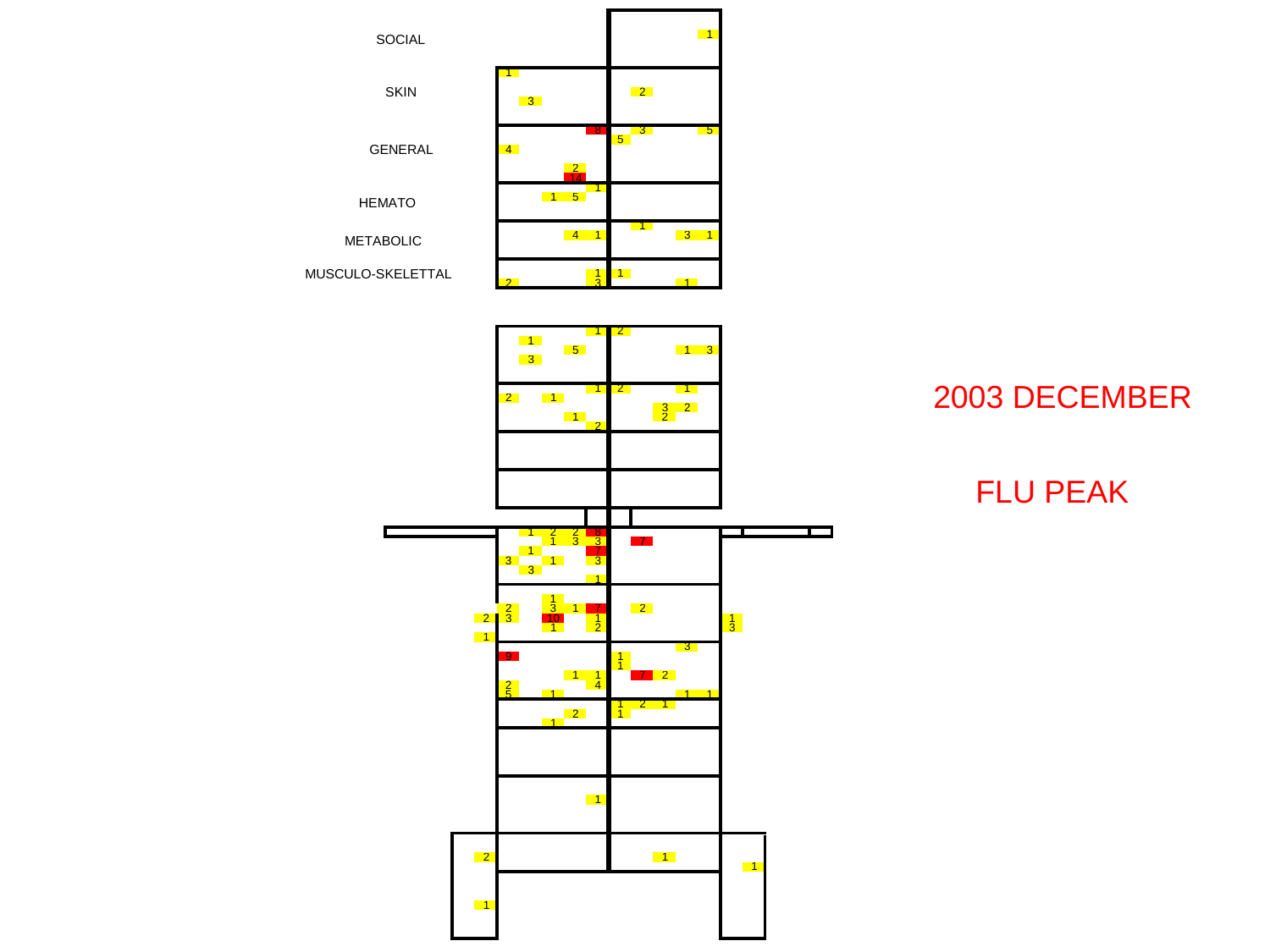

2003 DECEMBER
FLU PEAK

## Slide 80
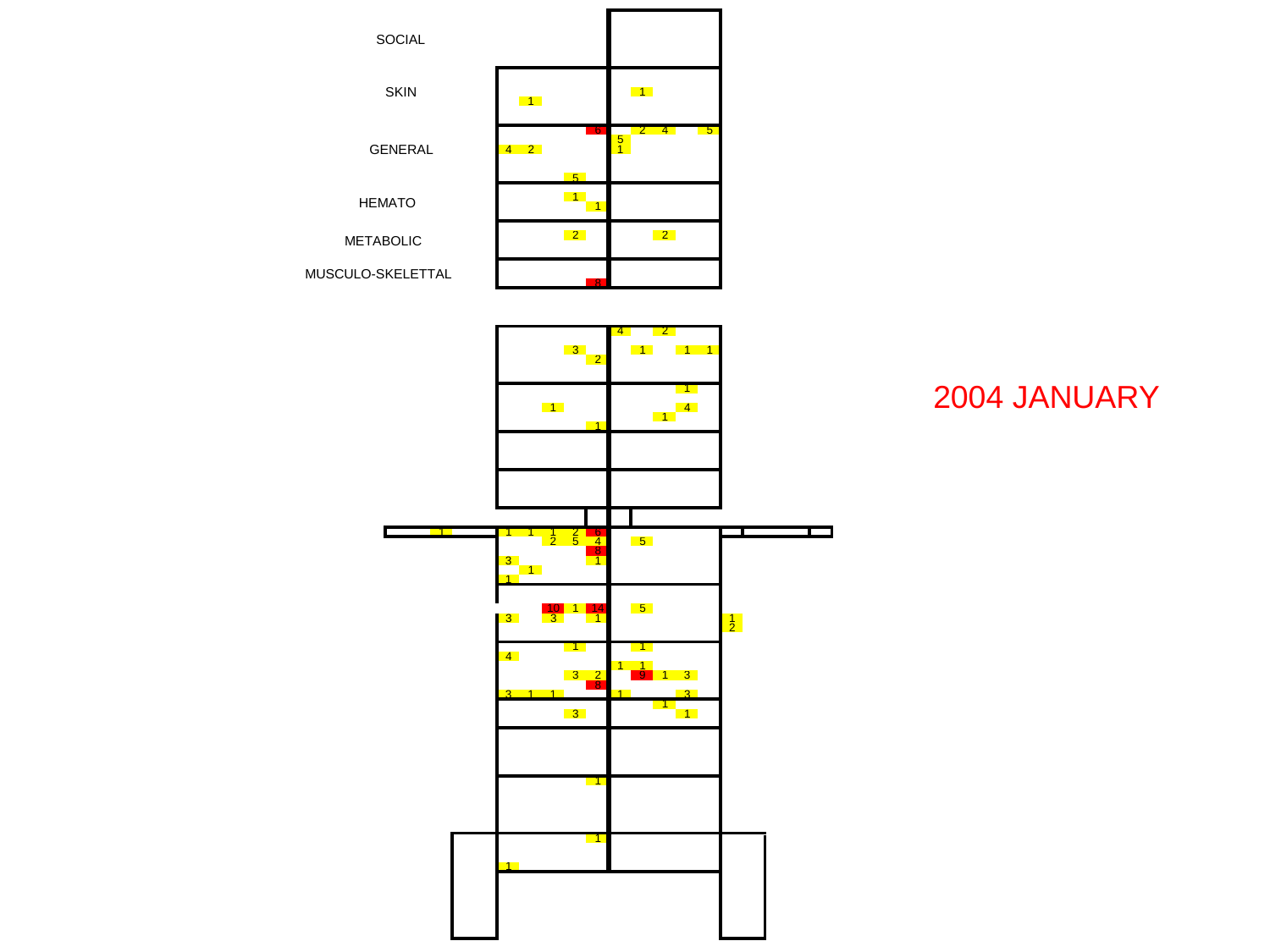

2004 JANUARY

## Slide 81
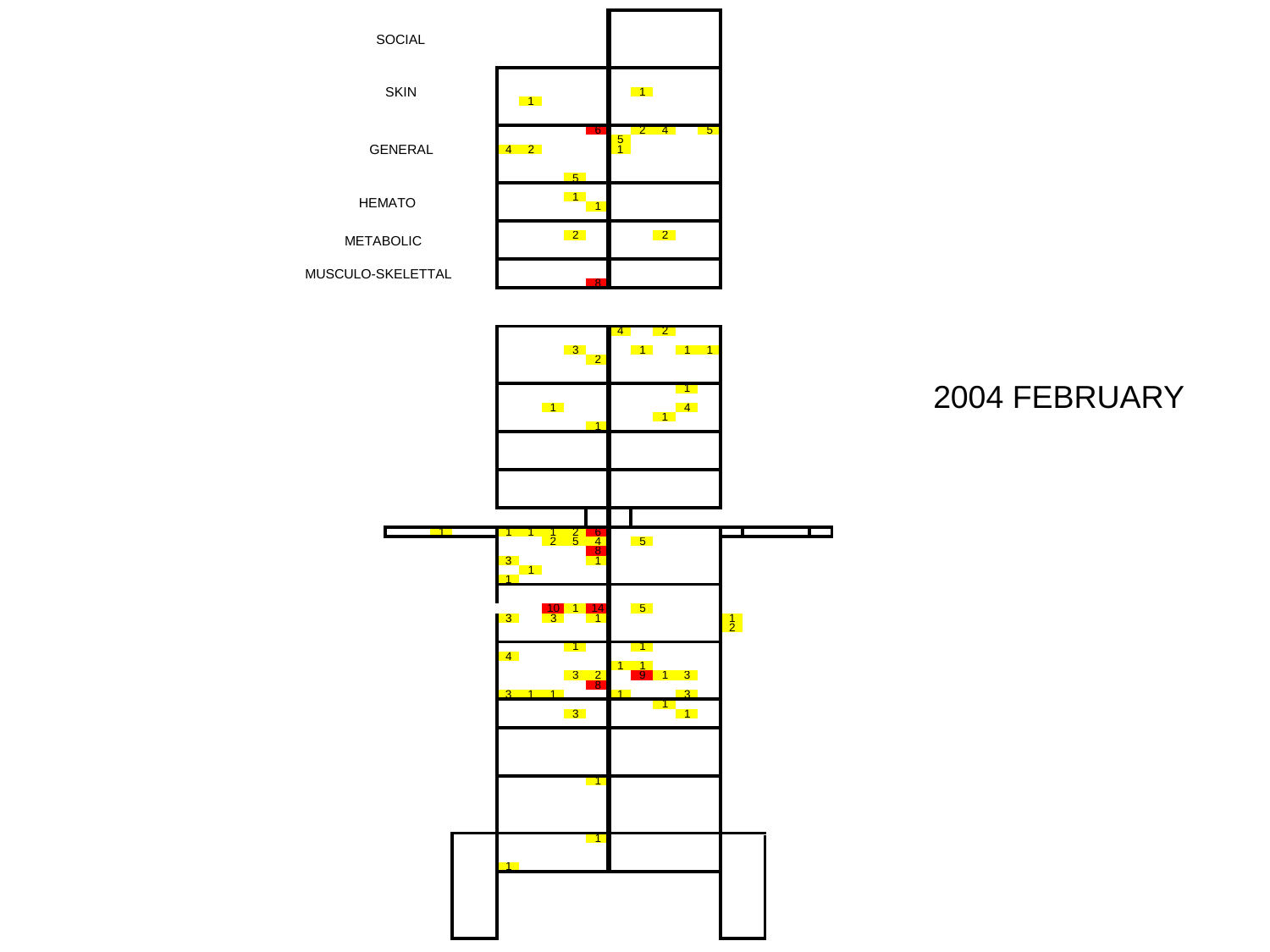

2004 FEBRUARY

## Slide 82
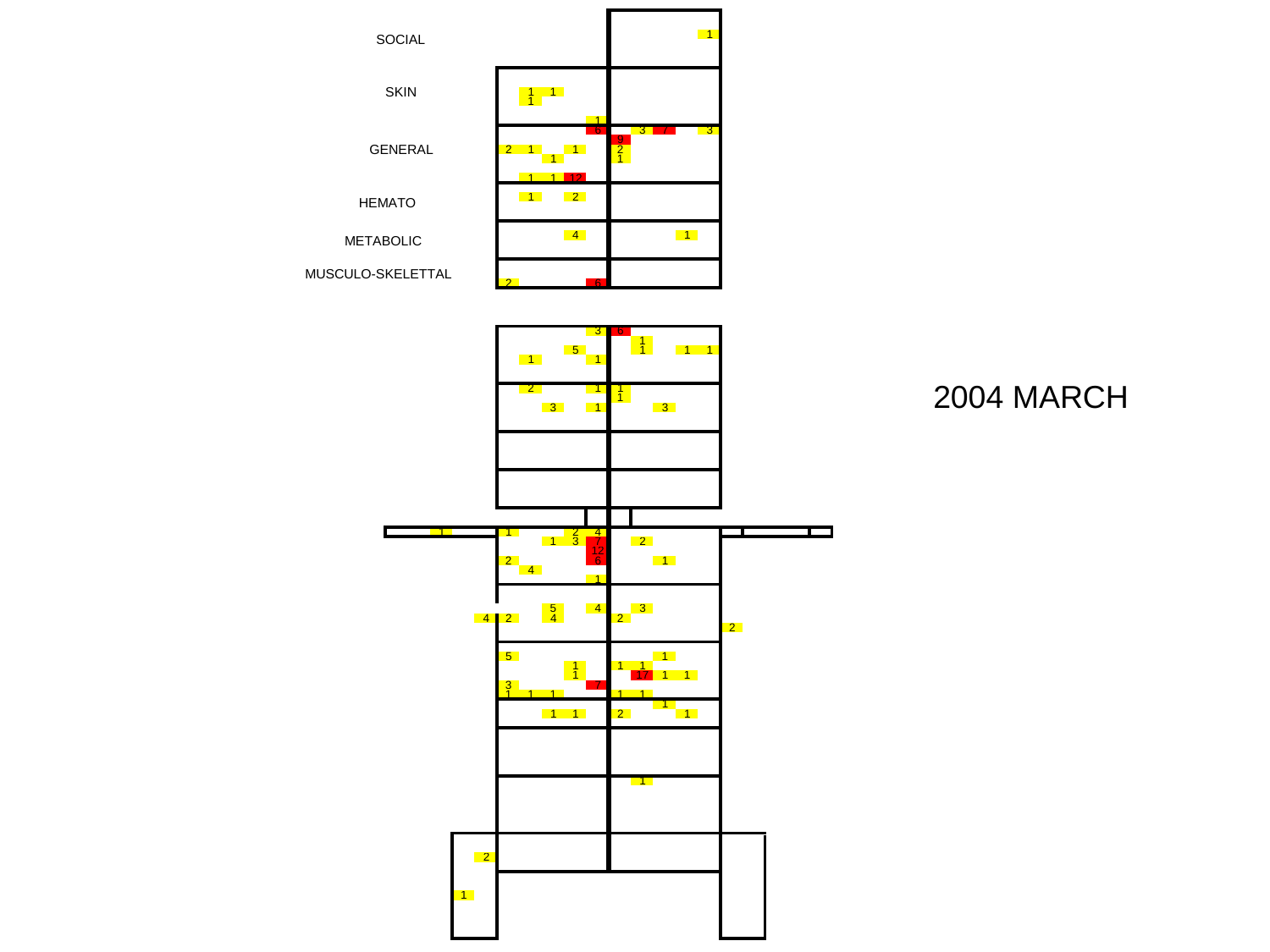

2004 MARCH

## Slide 83
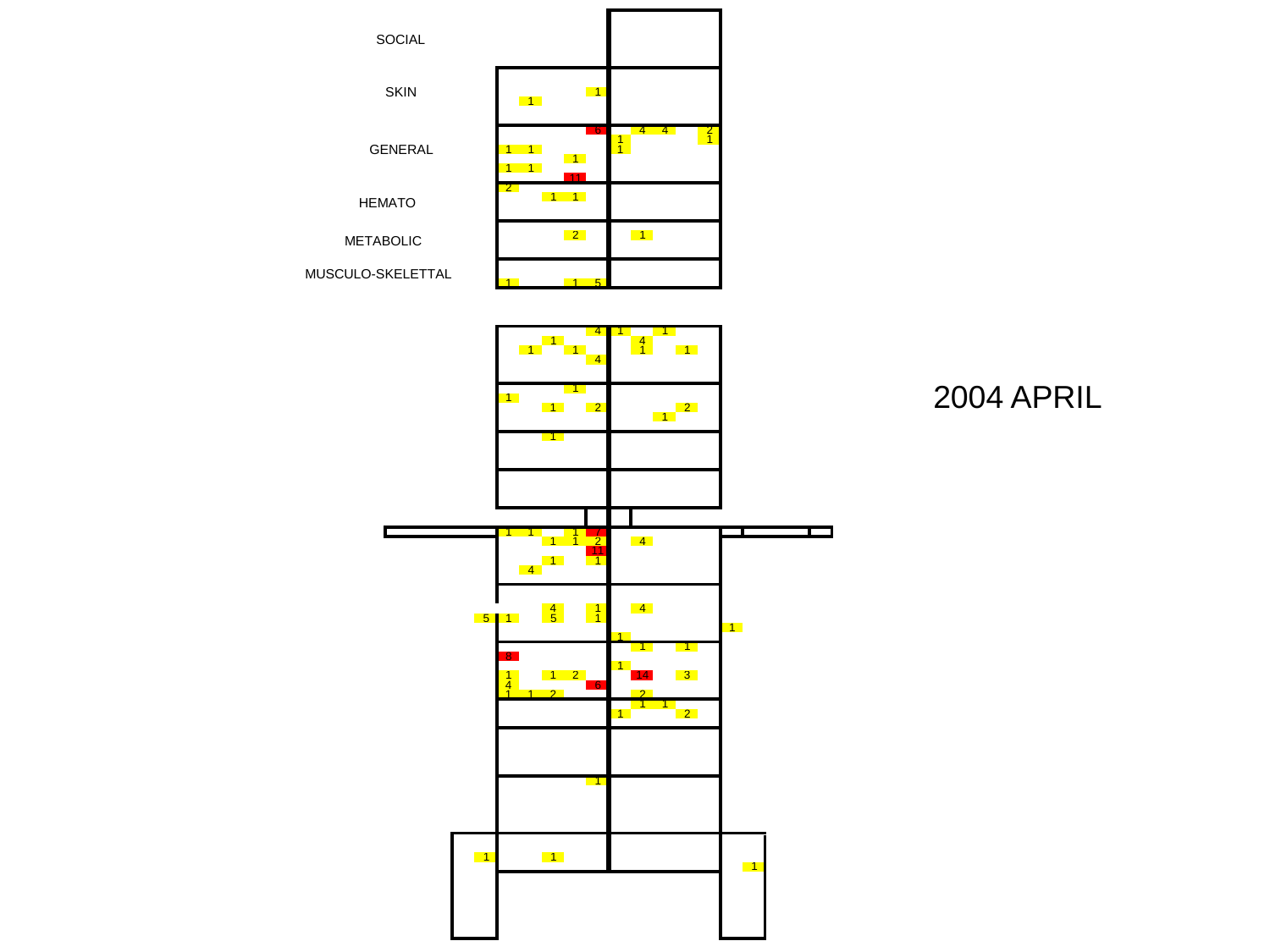

2004 APRIL
